# HIV-relevant policies in European countries: A comprehensive landscape of policies hindering or facilitating access to HIV prevention, testing, treatment, and care of key populations

**DOI:** 10.1371/journal.pone.0350099

**Published:** 2026-06-08

**Authors:** Katya A. Nogales Crespo, Joao V. Muniz Rocha, Patrick J. Campbell, Katherine L. Nelson, Allira Attwill

**Affiliations:** 1 Policy Wisdom LLC., Quebradillas, Puerto Rico‌‌; 2 Merck & Co., Inc., Rahway, New Jersey, United States of America‌‌; University of Arizona College of Medicine, UNITED STATES OF AMERICA

## Abstract

**Background:**

In Europe, there are variations in epidemic patterns, trends, and progress toward achieving the UNAIDS target of ending the AIDS epidemic. Complex social, economic, cultural, and behavioral dynamics make key population groups more vulnerable to HIV, and health and health-related policies play a significant role in ensuring progress towards eliminating HIV. This study aims to characterize and compare how HIV-relevant policies may directly or indirectly hinder or facilitate HIV prevention, testing, treatment, and care of key populations within selected European countries.

**Methods:**

This study consists of a targeted search of policies that may impact the HIV continuum of care for priority populations and a literature review to contextualize findings and explore policy implementation barriers. Analysis was conducted on a sample of 15 countries and six subregions. Data collection and analysis were done according to pre-defined policy areas (n = 57) identified through a review of international frameworks.

**Findings:**

A total of 445 policies and 69 studies met the eligibility criteria. In the broader analysis, 438 policies were identified across 57 policy areas, of which 248 policies across 27 policy areas were considered relevant to this manuscript. These policy areas address HIV prevention, testing, treatment, care, and structural support for key populations. In most countries, policies were identified covering 40 or more policy areas, highlighting substantial variation in policy scope across settings.

**Interpretation:**

Not all key and priority populations are equitably or adequately prioritized, with some policies leading to their needs and vulnerabilities being overlooked. Despite the presence of well-structured policies, misalignment between decision-makers’ attitudes and evidence-based recommendations, political constraints, and structural, cultural, and stigma-related factors persist. Health systems must address barriers faced by key and priority populations to achieve the UNAIDS targets.

## Introduction

As of 2024, over 2.6 million people are living with HIV in the World Health Organization European Region (WHO EURO), of which nearly 800,000 are in the European Union/European Economic Area (EU/EEA) [1,2]. The complex social, economic, cultural, and behavioral dynamics in Europe make key populations vulnerable to HIV and the structural barriers that prevent them from accessing HIV services. Coupled with an increasing rate of transmission in some countries [2] and the associated burden on health systems, addressing the HIV epidemic should be a health priority.‌‌

Variations across WHO EURO in epidemic patterns, trends [2], and progress towards achieving the UNAIDS targets [3] may be influenced by varying policy combinations, including those that directly and indirectly impact key HIV populations. Key populations, as defined by UNAIDS, are gay men and other men who have sex with men (MSM), sex workers, transgender people, people who inject drugs (PWID), and incarcerated people. These five groups are particularly vulnerable to HIV and often face inadequate access to essential prevention, treatment, and care services.

Gay men and other MSM remain heavily affected in the EU/EEA [2]; sex workers face a higher risk of HIV acquisition than the general population [4]; transgender people experience limited access to health services due to legal barriers, stigma, and discrimination [5]; PWID represent a substantial share of new diagnoses in Central and Eastern Europe [2], and prisons have a high HIV prevalence, with incarcerated individuals often facing limited access to the full care continuum [6]. Many HIV programs continue to neglect people from key populations and their sex partners, who account for an estimated 80% of new HIV transmissions outside sub-Saharan Africa [3].

Since 2008, the European Centre for Disease Prevention and Control (ECDC) and the WHO Regional Office for Europe (WHO/Europe) have undertaken an annual, coordinated HIV/AIDS Surveillance process. Their 2025 report highlights wide variations on HIV diagnosis and main transmission modes across European countries [2]. A study from 2009 measured and ranked the performance of HIV care provision from a patient viewpoint in Europe, identifying good practices and improvement areas such as restrictive legislation, social exclusion, and stigmatization [7]. Other recent studies reviewed HIV-related policies across some dimensions of the HIV continuum of care in European countries, such as HIV testing [8], or harm reduction programs for HIV prevention [9], or for some key populations (such as sex workers) [10]. Recent data regarding progress towards the 95–95–95 testing, treatment and viral load suppression targets indicates variations between regions and countries [3,11,12], which reflect the complex dynamics of social, economic, and country contexts in the region and how these conditions impact access to or uptake of services across the HIV continuum of care across countries [13]. These studies highlight priorities for improving HIV control, including expanding testing in primary care, emergency departments, and community settings, reducing late diagnosis through earlier detection and timely linkage to care, particularly for undocumented migrants, and addressing structural barriers such as punitive drug laws, resistance to harm reduction, inequalities in treatment access, HIV-related stigma and discrimination, and restrictive sex work policies [11].

Despite the attention given to policies related to HIV and key populations in Europe, no current studies concurrently examine the range of policies across the HIV continuum of care for these populations. The aim of this study is to characterize and compare HIV-relevant policies, including those directly and indirectly related, and to explore their potential impact on HIV prevention, testing, treatment, and care for key and priority populations across Europe and within select European countries.

## Methods

### Study design

This study is part of a larger project aiming to better understand how HIV-relevant policies relate to key and priority populations across the continuum of care, considering normative and structural characteristics and health system features, covering 57 policy areas (Table 1). The current paper analyses a subset of 27 policy areas that are of particular importance for key and priority populations.

**Table 1:** Inclusion and exclusion criteria.

Criteria for inclusion and exclusion of policies and peer-reviewed papers
• The policy must address one or more of the policy areas.
• The policy must be government-led and/or publicly funded and can be a policy, strategy, program, service, guideline, standard, or legislation.
• The policy must be issued by a national or subnational (for selected subnational regions) authority. The issuing authority must be a state actor or an organization that is commissioned by the government.
• The policy must directly or indirectly impact access to or uptake of services across the HIV continuum of care for key and/or priority populations.
• The policy must be valid at the time of search and not be superseded nor have an endorsement, launch, or publication date earlier than 2010 (except for laws or legislations). If the policy is a law or legislation enacted before 2010, it should be included if it continues to be in force.

### Study population and sample

UNAIDS considers gay men and other men who have sex with men, sex workers, transgender people, people who inject drugs, and incarcerated people as the HIV key populations [14]. With a larger proportion of new transmissions identified among these groups and their partners (54% of new HIV transmissions in 2018), these populations are also typically socially marginalized and targeted with punitive laws and practices [15]. Other priority populations were considered in this study, as identified in the national HIV plans/strategies (which sometimes consider other groups to be particularly vulnerable to HIV in their setting).

A sample of 15 European countries was selected using a structured, non-random approach designed to ensure diversity and representation across key contextual dimensions. Country selection followed a predefined seven-step process, in which countries were sequentially included based on epidemiological, geographic, socio-political, and health system criteria. Initial selection prioritized countries with the highest HIV burden, based on incidence and prevalence. Additional countries were then incorporated to ensure variation in progress towards UNAIDS targets, geographic representation across European subregions, and diversity in income levels. The process also accounted for differences in social and policy environments relevant to key populations, including levels of discrimination and regulatory approaches, as well as variation in health system models and service coverage. This iterative approach ensured that the final sample captured a broad range of policy and contextual settings relevant to the study objectives. Further details on the selection criteria and stepwise procedure are provided in the Supplementary Material to support transparency and replicability (Appendix I in S1 File). Since some European countries have implemented a decentralized system in which decision-making and healthcare management often occur at the regional or local levels, six subnational regions from decentralized countries were also selected.

The list of countries and subnational regions selected for this study was as follows: Belgium (+Flanders), Czech Republic, Estonia, France, Germany (+Berlin), Italy (+Lombardi), Latvia, the Netherlands, Poland, Portugal, Romania, Spain (+Catalonia), Sweden, Switzerland (+Vaud), and the United Kingdom (+England).

### Search strategy and selection criteria

To detect relevant policies and identify the barriers, challenges, and lessons learned from their implementation, the data search included a) targeted policy search based on pre-defined policy areas, and b) a peer-reviewed literature search using search queries.

### Targeted policy search

**Fig 1:**
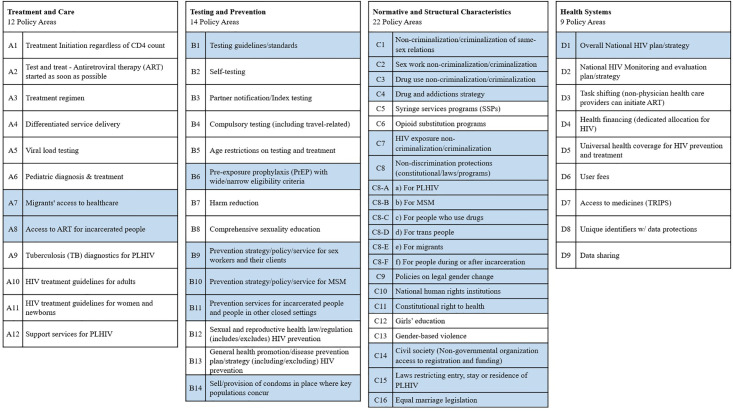
Policy areas. Note: Highlighted boxes indicate the policies included in this paper.

The targeted policy search was conducted to identify policies that may impact HIV efforts. The search was conducted using 57 policy areas distributed across treatment and care, testing and prevention, normative and structural characteristics, and health systems features. The list of policy areas was built based on a review of the HIV Policy Lab [16], international organizations frameworks (WHO, UNAIDS, United Nations), and a pilot run through an open search to verify and identify additional policy areas. For each one, a code (Policy ID) was defined. Codes were also used to organize and analyze the peer-reviewed papers. Fig 1 presents the complete list of policy areas, highlighting the 27 selected for the current study. These were selected as they are either designed to provide HIV prevention, testing, treatment, and/or care, or create normative or structural conditions that can resolve or perpetuate barriers to care for key populations. A thorough description of the policy areas can be found in the Supplementary Material (Appendix II in S1 File).

The template was developed as a pre-structured guide to systematically organize and standardize data extraction across countries and policy areas. Evidence collection was conducted between April and June 2023 by six trained researchers, who populated the template following predefined dimensions of analysis. Policies were identified through open web searches using combinations of keywords related to HIV and the predefined 27 policy areas and the four overarching dimensions, as well as terms such as policies, strategies, guidelines, standards, and legislation. Searches were conducted in both English and the official national languages of the selected countries, using government websites, institutional repositories, and relevant organizational sources. Where necessary, translation into English was performed using a forward-backward method supported by Google Translate, with cross-checking by researchers to ensure accuracy and preserve the meaning of key concepts. Data search, collection, and initial interpretation were carried out in the original language of each country. Completed templates underwent two rounds of revision by two co-authors (KANC and JVMR) to ensure consistency, completeness, and alignment across countries.

For the 57 policy areas, there were identified 438 policies. In this current study, the 27 policy areas are addressed by 248 policies.

### Peer-reviewed literature search

Two search queries were developed using country names and policy types (e.g., policies, strategies, guidelines, standards, legislation); one query was linked to HIV and the other to HIV key populations (see Supplemental Material for search terms- Appendix III in S1 File). The search was conducted throughout January 2023 on PubMed, Web of Science, and Google Scholar. To be considered, articles had to be published in a peer-reviewed journal no earlier than 2015, analyze one or more of the sampled countries and/or subregions, and be in English or other national languages. A snowball technique was used to identify additional articles based on the reference list in the included articles. A flowchart was developed to represent the outcomes of this process, according to the PRISMA 2020 flow diagram template for systematic reviews [17]. The following information was extracted from the included papers: title, year, country, policy area (according to the Policy ID), and findings on policy implementation and barriers/challenges.

**Fig 2:**
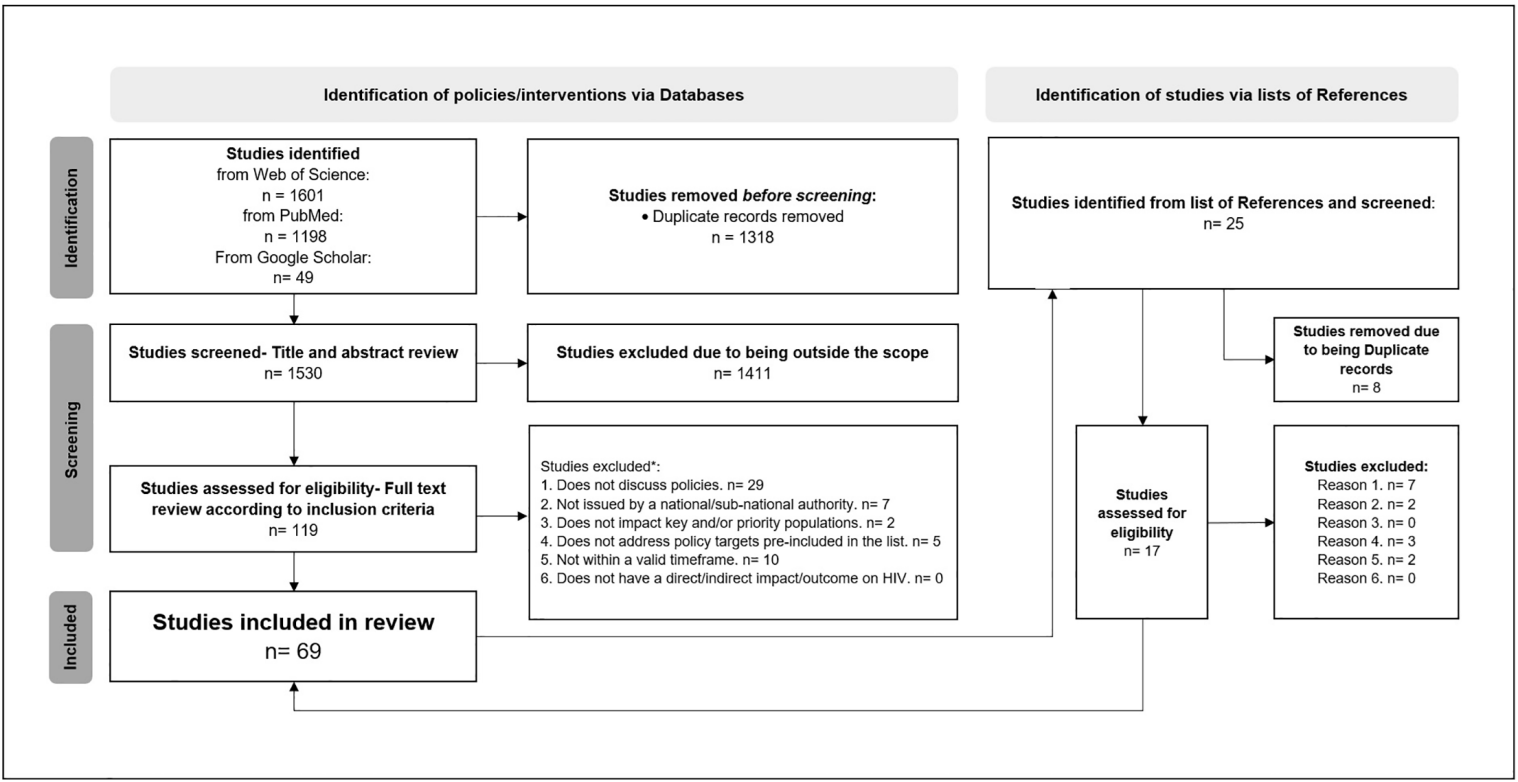
Flowchart of peer-reviewed literature selection process.

A total of 2,848 articles were identified. After screening, a total of 69 studies were included in the study (Fig 2). The final list of studies and an overview of findings are provided in the Supplementary Material (Appendix IV in S1 File). For this analysis, 50 of the 69 studies were addressing the 27 policy areas and used in the analysis.

Inclusionary and exclusionary criteria for policies both for the Targeted policy search and the Peer-reviewed literature search are listed in Table 1.

### Data analysis

Policies were analyzed to determine whether they directly or indirectly hinder or facilitate prevention, testing, treatment, and linkage and retention to care of key and priority populations according to criteria. Policies with direct impact were defined as those that identified HIV prevention, testing, treatment, or care as a policy objective. On the other hand, policies with an indirect impact defined an objective other than HIV prevention, testing, treatment, or care, but that may nonetheless influence access to or uptake of services across the continuum of care of key and priority populations. An analysis of the measures and activities stipulated by policies was also conducted to assess whether they might hinder or facilitate access to or uptake of services across the HIV continuum of care. Assessment was done according to whether the policy or its implementation requirements prohibited, limited, or eased access of key and priority populations to services and/or interventions. This analysis was contextualized with findings from peer-reviewed studies. The discussion of results was conducted for each key population, as well as an overview of priority populations identified

### Ethics statement

Ethical approval was not required for this study. The work consisted of a literature review and an analysis of policy documents, and it did not involve human participants. No interviews were conducted and no human perspectives or data were collected by the authors.

## Results

In the broader study we identified 438 policies across 57 policy areas. Those deemed relevant to this manuscript include 248 policies across 27 policy areas (Fig 1). In most countries, policies were identified addressing 40 or more policy areas. Table 2 provides a summary of the HIV-relevant policies found for countries and subnational regions. The complete dataset of policies is provided in the Supplementary Material (Appendix V in S1 File).

**Table 2:** Summary of overall national/subnational policies in selected European countries.

Country or subnational region	Total number of policy areas addressed *(out of 57)*		Summary of policies identified by category							
			*Treatment and Care*		*Testing and Prevention*		*Normative and Structural*		*Health Systems*	
	Total (N = 57)	This analysis (N = 27)	Total (N = 12)	This analysis (N = 2)	Total (N = 14)	This analysis (N = 6)	Total (N = 22)	This analysis (N = 18)	Total (N = 9)	This analysis (N = 1)
Belgium	39	22	6	2	8	4	18	15	7	1
*Belgium (Flanders)*	10	7	0	0	3	1	7	6	0	0
Czech Republic	47	23	11	2	12	6	18	14	6	1
Estonia	42	20	8	1	11	5	16	13	7	1
France	42	20	9	1	11	5	15	13	7	1
Germany	34	21	3	2	6	3	18	15	7	1
*Germany (Berlin)*	6	2	0	0	2	0	3	1	1	1
Italy	42	19	12	2	10	5	13	11	5	1
*Italy (Lombardy)*	4	0	3	0	1	0	0	0	0	0
Latvia	36	12	10	1	6	1	13	9	7	1
Netherlands	43	20	10	1	9	3	16	15	8	1
Poland	43	18	11	2	9	2	16	13	7	1
Portugal	49	22	10	2	11	4	19	15	9	1
Romania	42	23	6	1	10	4	19	17	7	1
Spain	46	23	9	1	10	5	18	17	9	1
*Spain (Catalonia)*	17	9	2	0	4	1	10	7	1	1
Sweden	40	18	8	0	10	4	16	13	6	1
Switzerland	39	24	6	2	8	4	19	17	6	1
*Switzerland (Vaud)*	9	3	2	1	2	0	5	2	0	0
United Kingdom	35	17	9	2	6	2	15	13	5	0
*United Kingdom (England)*	13	6	1	0	6	4	3	1	3	1

The rows in italic indicate the subregions selected, with their respective countries indicated in parentheses

Table 3 provides a summary of key and priority populations referenced in national/subnational policies in selected European countries. All the countries analyzed had an overarching national HIV plan or strategy. Notably, in the Netherlands and England, these policies do not identify key populations as their targets. In all countries except Latvia, Poland, and United Kingdom, prevention strategies, policies, or services targeting MSM, sex workers, or incarcerated people were identified, either within the national HIV plan or as stand-alone policies. Twelve countries have PrEP and testing guidelines that clearly identify eligible key populations or discuss the importance of increasing testing among them.

**Table 3:** Summary of key and priority populations referenced in national/subnational policies in selected European countries.

Country or subnational region	HIV plan/strategy	Key and priority populations identified in national HIV plan/strategy	Specific prevention strategy/policy/service for key populations within national HIV plan/strategy or standalone policy			Key populations included in PrEP eligibility criteria policies					Key populations included in testing guidelines policies				
			Gay men and MSM	Sex workers	Incarcerated people	Gay men and MSM	Sex workers	Transgender people	PWID	Incarcerated people	Gay men and MSM	Sex workers	Transgender people	PWID	Incarcerated people
Belgium	National HIV Plan 2020–2026	Key populations: Gay men and other MSM; Sex workers; Transgender people; PWID; Incarcerated people Priority populations: Migrants from high HIV prevalence countries	Yes	No	No	Yes	Yes	Yes	Yes	No	Yes	Yes	No	Yes	No
*Belgium (Flanders)*	*NA*	*NA*	*No*	*Yes*	*No*	*NA*	*NA*	*NA*	*NA*	*NA*	*NA*	*NA*	*NA*	*NA*	*NA*
Czech Republic	National program for solving the problem of HIV/AIDS 2023–2027	Key populations: Gay men and other MSM; Sex workers; Transgender people; PWID; Incarcerated people Priority populations: Migrants from high HIV prevalence countries	Yes	Yes	Yes	Yes	Yes	Yes	Yes	Yes	Yes	Yes	Yes	Yes	Yes
Estonia	National HIV Action Plan 2017–2025	Key populations: Gay men and other MSM; Sex workers; PWID; Incarcerated people	Yes	Yes	Yes	Yes	Yes	No	Yes	No	Yes	Yes	No	Yes	No
France	National Strategy for Sexual Health 2017–2030 National Plan to Combat HIV/AIDS and STIs 2010–2014	Key populations: Gay men and other MSM; Sex workers; Transgender people; PWID; Incarcerated people Priority populations: Migrants from high HIV prevalence countries; Lesbian women	Yes	Yes	Yes	Yes	Yes	Yes	Yes	No	Yes	No	No	No	No
Germany	Integrated Strategy for HIV, Hepatitis B and C and Other STIs 2016-Undefined	Key populations: Gay men and other MSM; Sex workers; Transgender people; PWID; Incarcerated people Priority populations: Migrants from high HIV prevalence countries; Youth; Pregnant women	No	Yes	Yes	Yes	No	Yes	Yes	No	NA	NA	NA	NA	NA
*Germany (Berlin)*	*Framework concept for the prevention of HIV/AIDS, hepatitis and STIs 2009-Undefined*	*Key populations: Gay men and other MSM; Sex workers; PWID; Incarcerated people* *Priority populations: Migrants from high HIV prevalence countries*	*No*	*No*	*No*	*NA*	*NA*	*NA*	*NA*	*NA*	*NA*	*NA*	*NA*	*NA*	*NA*
Italy	National Plan of Interventions Against HIV and AIDS 2017–2019	Key populations: Gay men and other MSM; Sex workers; Transgender people; PWID; Incarcerated people Priority populations: Migrants from high HIV prevalence countries; PLHIV; Partners of PLHIV	Yes	Yes	Yes	Yes	No	Yes	Yes	No	No	No	No	Yes	Yes
*Italy (Lombardy)*	*NA*	*NA*	*No*	*No*	*No*	*NA*	*NA*	*NA*	*NA*	*NA*	*NA*	*NA*	*NA*	*NA*	*NA*
Latvia	Action plan for limiting the spread of HIV infection, STIs, hepatitis B and C 2018–2020	Key populations: Gay men and other MSM; Sex workers; PWID; Incarcerated people Priority populations: Pregnant women	No	No	No	*NA*	*NA*	*NA*	*NA*	*NA*	No	No	No	No	No
Netherlands	National Action Plan for STI, HIV and sexual health 2017–2022	Priority populations: General population; PLHIV	No	No	Yes	Yes	No	Yes	No	No	Yes	No	No	Yes	No
Poland	National Program of Prevention of HIV Infection and Combating AIDS 2022–2026	Key populations: Gay men and other MSM; Sex workers; Transgender people; PWID; Incarcerated people	No	No	No	No	No	No	Yes	No	Yes	Yes	No	Yes	No
Portugal	National Health Program in the areas of HIV infection and AIDS 2012–2016	Key populations: Gay men and other MSM; Sex workers; PWID; Incarcerated people Priority populations: Migrants	No	No	Yes	No	No	No	Yes	No	Yes	Yes	No	Yes	Yes
Romania	National strategy for the surveillance, control, and prevention of cases of HIV/AIDS infection 2022−2030	Key populations: Gay men and other MSM; Sex workers; Transgender people; PWID; Incarcerated people	Yes	No	Yes	NA	NA	NA	NA	NA	NA	NA	NA	NA	NA
Spain	Plan for the prevention and control of infection by HIV and STIs 2021–2030	Key populations: Gay men and other MSM; Sex workers; Transgender people; PWID; Incarcerated people Priority populations: Migrants; People who use drugs during sex	Yes	Yes	Yes	Yes	Yes	Yes	Yes	No	Yes	Yes	Yes	Yes	No
Spain (Catalonia)	Action plan for the fight against HIV and other STIs 2021–2030	Key populations: Gay men and other MSM; Sex workers; Transgender people; PWID; Incarcerated people Priority populations: Migrants from high HIV prevalence countries	No	No	No	Yes	Yes	Yes	Yes	No	NA	NA	NA	NA	NA
Sweden	National strategy against HIV/AIDS and certain other infectious diseases 2017-Undefined	Key populations: Gay men and other MSM; Sex workers; Transgender people; PWID; Incarcerated people Priority populations: Migrants from high HIV prevalence countries; Youth and young adults at risk	Yes	Yes	Yes	Yes	Yes	Yes	Yes	No	No	No	No	No	No
Switzerland	National Programme on HIV and other STIs 2011-2017 (extended to 2023)	Key populations: Gay men and other MSM; PWID; Incarcerated people	Yes	No	Yes	No	No	Yes	No	No	Yes	Yes	No	Yes	Yes
*Switzerland (Vaud)*	*NA*	*NA*	*No*	*No*	*No*	*NA*	*NA*	*NA*	*NA*	*NA*	*NA*	*NA*	*NA*	*NA*	*NA*
United Kingdom	Each of the 4 nations that make up the UK (England, Ireland, Scotland, and Wales) have standalone HIV plans	NA	No	No	No	Yes	No	No	No	No	Yes	No	Yes	Yes	No
*United Kingdom (England)*	*An action plan towards ending HIV transmission, AIDS, and HIV-related deaths 2022–2025*	*Key populations: Gay men and other MSM* *Priority populations: Black Africans; Younger adults*	*Yes*	*Yes*	*Yes*	*NA*	*NA*	*NA*	*NA*	*NA*	*NA*	*NA*	*NA*	*NA*	*NA*

NA: Not applicable, as no policy was found. MSM: Men who have sex with men. PLHIV: People living with HIV. PWID: People who inject drugs. STI: Sexually transmitted infections.

### Key and priority populations analysis

Across countries, policies related to stigma, discrimination, and criminalization of HIV vary. Latvia, Poland, and Romania have specific laws that criminalize HIV exposure and/or transmission, while Belgium, Czech Republic, and France, have seen non-disclosure of HIV status used as grounds for prosecution cases, despite it not being criminalized [Policy C7] [18]. There have also been cases in Belgium, France, Germany, Italy, and England where scientific evidence regarding Undetectable = Untransmittable has been used to acquit PLHIV and dismiss cases [18]. Besides variation in policies and inconsistent enforcement, peer-reviewed studies show that PLHIV receive inconsistent information from healthcare providers, causing them to individually interpret the rules of conduct regarding disclosure of HIV status to potential sexual partners [19], and that their understanding of legal obligations in this respect is weak [20]. While policies addressing key populations are present across countries, differences exist in legal frameworks, enforcement, and the extent to which specific population needs are reflected. Country policy comparisons and insights for each key population are listed in the sections below.

### Gay men and MSM

Nearly all the analyzed countries include gay men and MSM in national HIV plans (except the Netherlands), testing guidelines, and PrEP policies. Nine countries have specific prevention policies and/or services targeting this group, with comprehensive approaches seen in Italy, Czech Republic, France, Spain, and Switzerland, that integrate PrEP with other prevention strategies, such as stigma reduction programs, testing, condom and lubricant distribution, PEP, and treatment as prevention to achieve viral suppression (TasP) [Policy B10]. In Belgium, Czech Republic, and Portugal, the national HIV plan explicitly mentions the provision of condoms in saunas targeting this group [Policy B15].

Structural factors such as stigma and discrimination based on sexual orientation hinder the availability, access, and uptake of HIV interventions. Same-sex relations have been decriminalized in all countries analyzed [Policy C1], and nearly all countries analyzed have national laws that protect people from discrimination based on sexual orientation, including discrimination in healthcare settings. However, in Czech Republic, the Netherlands, and Switzerland, discrimination against gay men and MSM is diluted, being included in a broad antidiscrimination policy protecting all people [Policy C8-B], and in Latvia and Italy, no anti-discrimination policies were identified.

Results from the peer-reviewed search indicate how gay men and MSM are subjected to stigma and discrimination. In Italy, legalization of same-sex marriage was opposed by the country’s Constitutional Court, political establishment, and the Catholic Church [21]. In Poland, local councils adopted non-legally binding resolutions against “LBGT ideology” in 2019 [22]. A French study found that individuals with low levels of outness (i.e., few or no acquaintances know about their sexual orientation) experienced higher PrEP access barriers than those with medium or high levels of outness, due to limited access to information and services and a reluctance to discuss their sexuality with medical providers [23].

### Sex workers

Six countries (Czech Republic, Estonia, France, Italy, Spain, and Sweden) include prevention services for sex workers in their national health plans [Policy D1]. However, nuances within these policies make accessing and uptaking HIV prevention among sex workers more complicated than it should be. In Estonia, key services like free diagnostics and treatment of STIs are only available in the capital city [Policy D1], resulting in geographic inequities. Sweden’s HIV-related policies only describe high-level measures and lack monitoring indicators [Policy D1]. Italy includes PEP and PrEP for sex workers in the 2017–2019 prevention plan, but they are not yet listed as a beneficiary group in PrEP guidelines [Policy B6].

None of the countries in this study fully criminalize sex work, with Germany and the Netherlands exhibiting the highest level of decriminalization where sex work is legal and regulated [Policy C2]. Most countries allow sex work if not part of an organized activity in an effort to combat the sexual exploitation and trafficking of individuals. Running brothels, procuring sex workers, and exploiting the prostitution of others are considered criminal offenses. In Sweden and France, selling sex is not criminalized, while purchasing sex is [Policy C2] [4]. Even in countries that decriminalized aspects of sex work, studies show persistent stigma and vulnerabilities. In the Netherlands (a country widely considered to be very liberal) sex workers still avoid health check services and refuse to assert worker rights, indicating that stigma associated with sex work continues [24]. In Latvia, PLHIV or AIDS are prohibited from engaging in prostitution [Policy C2]. In Germany, there are no health examination requirements for sex workers and no mandatory condom use for most of the country, with clients and brothel managers often pressuring sex workers to decline using condoms to increase profits [24].

### Transgender people

Transgender people are not always identified as a priority population in national HIV plans, HIV testing guidelines, and/or PrEP policies. There were found constraints regarding gender change in policies of four countries [Policy C9]. Sex change policy in Czech Republic focuses on gender-affirming surgery, rather than a person’s preferred pronoun, while Poland requires a civil suit against one’s parents. In Romania, it is unclear if surgery is required to change the sex listed on a birth certificate, and Latvia does not have legal dispositions for sex change altogether. Although policies in Western and Southern European countries are more inclusive of transgender people, progress is still recent, as exemplified by Italy [25] and France [Policy C9], where surgery was a mandatory requirement to legally change sex until 2015 and 2018, respectively.

Eight countries (Belgium, Czech Republic, France, Germany, Portugal, Spain, Sweden, and United Kingdom) currently have policies to combat discrimination based on gender and gender identity [Policy C8-D]. Three countries (the Netherlands, Romania, and Switzerland) have general anti-discrimination policies based on gender. There was no evidence found of anti-discrimination policies for transgender people in Italy, Estonia, Latvia, or Poland.

### People who inject drugs

Most national HIV plans, testing guidelines, and PrEP policies consider PWID a priority population. All countries have policies criminalizing drug possession, except Czech Republic and Portugal [Policy C3], and seven countries consider drug use itself as an offence (Estonia, France, Latvia, Romania, Spain, Sweden, and Switzerland). Strategies addressing drug and addiction were found for all countries except Sweden and the Netherlands and included stated aims to decrease the risk of associated HIV/AIDS infection and vulnerability in most countries, excluding Spain, Switzerland, and the United Kingdom. Results from the peer-reviewed literature search show that the human rights of drug users are violated in some instances. For example, in Estonia, private medical information of women living with HIV and/or drug-dependency can be disclosed to the police, employers, and family members, contributing to stigmatization and deterring uptake of HIV services [26]. In Poland, drug use is positioned as a criminal issue as opposed to a healthcare issue [27].

### Incarcerated people

Prisons are acknowledged in the national HIV plans of all countries [Policy D1], but incarcerated people are not commonly identified as a key population in testing guidelines or PrEP policies (except for Czech Republic [Policy B6]). A study in Germany discusses that prison conditions, administrative procedures, political constraints, and social stigmatization limit incarcerated people’s access to rights and services, including healthcare and disease prevention [28]. Additionally, some prisons deviate from national guidelines when providing HIV treatment prescriptions. For example, antiretroviral medications are not accessible in 10.8% of Italian prisons, or prison doctors provide prescriptions which do not follow the corresponding national guidelines (17.6% of prisons in Belgium, 5.7% in Italy and 3.6% in Austria) [29].

Policies regarding access to antiretroviral therapy (ART) for incarcerated people were identified in 12 countries [Policy A8]. While specific mention of ART is made in the Czech Republic, Estonia, and Italy, in most cases, ART is provided without specific targeting. In France, the Netherlands, and Sweden, constitutional rights to health may allow incarcerated people access to ART, although its implementation has been questioned [30]. Implementing harm reduction programs in prisons is also challenging, as studies in Germany and Portugal have reported the reluctance of incarcerated people to participate due to the lack of confidentiality, fear of discrimination, and fear of being subjected to reprisals by prison authorities [31,32].

### Priority populations

Six groups were identified as HIV priority populations in focus countries: Migrants from high HIV prevalence countries, lesbians, pregnant women, people who use drugs during sex (‘chem sex’), youth and young adults, and Black Africans [Policy D1]. Migrants from countries with a high HIV prevalence are recognized as priority populations in the national HIV plans of Belgium, Czech Republic, France, Germany, Italy, Portugal, Spain, and Sweden. Migrants, although not formally classified as a key population by UNAIDS [14], remain particularly vulnerable due to barriers in healthcare access, social exclusion, and risk of exploitation [33], with a significant proportion of HIV cases in the EU/EEA occurring among migrants who acquired HIV after arrival [2,34].

In Spain and Italy, changes in the universal healthcare system due to financial crisis [35] and gaps between official policies and the real provision of non-urgent medical care [36] impacted migrants’ access to free healthcare services, with civil society actors and non-governmental organizations (NGOs) playing a crucial role as a safety net system [36,37]. Variations were found in how policies frame the migrants access to HIV services. In Portugal, Italy, and Spain, regardless of their legal status in the country, migrants have the same right to health protection as recognized citizens and legal residents. In contrast, in the Netherlands, undocumented migrants who lack access to public services, are entitled to receive the same care as an insured person but must cover the cost out-of-pocket [38]. Policies in Belgium, France, Germany, and Switzerland are unclear regarding access to HIV services for migrants, regardless of their legal status.

## Discussion

To our knowledge, this study represents the first effort to comprehensively examine policies affecting all HIV key populations across the full HIV care continuum. Importantly, the aim is not to assess or evaluate the impact of these policies, but rather to map and analyze their relevance. Moreover, this study takes an innovative approach by combining a thorough literature review with an in-depth analysis of a broad set of policies, providing a contextualized understanding of policy impact and insights into the realities faced by key populations. Our analysis identified variations across European countries regarding how existing policies directly or indirectly hinder or facilitate HIV prevention, testing, treatment, and care of key populations.

Our findings indicate that key populations are not consistently or adequately prioritized in policymaking, which may hinder progress toward UNAIDS targets. While many countries have policy frameworks that reference these groups, gaps remain in how they are defined, recognized, and addressed. As a result, some key populations are not clearly identified as beneficiaries, and their specific needs and vulnerabilities may be overlooked in policy development. Even in countries with well-structured policies that align to the needs of key populations, several policy implementation barriers (e.g., misalignment between decision-makers’ attitudes and evidence-based recommendations, and structural, cultural, and stigma-related factors) have been identified, hampering effective prevention and treatment efforts. These patterns suggest that policy recognition of key populations is often partial or inconsistent, which may limit the comprehensiveness of national HIV responses [39], or even serve as barriers to effective HIV health services [40].

Across countries, cross-cutting structural barriers continue to shape the effectiveness of HIV policies for key populations. High levels of stigma and discrimination, reinforced by misconceptions surrounding HIV, together with criminalization, limit access and drive poor engagement to HIV prevention, testing, and treatment services [11,41]. Discrimination based on sexual orientation persists [42], despite progress in legal recognition, such as marriage equality, which may reflect broader societal acceptance; at the same time, gay men and other MSM remain disproportionately affected by HIV [2], and policy recognition and targeted interventions have only recently been strengthened in some settings, such as in the Czech Republic, where their inclusion as a key population was only endorsed in the latest HIV plan [43]. Transgender people continue to face discrimination and insufficient policy recognition, with examples such as Romania, where they are mentioned under MSM in national strategies [44], overlooking their specific needs. Legal gender recognition policies can have positive effects on health care utilization [45] and may also reflect broader societal attitudes, including lower levels of stigma toward transgender individuals [46].

Criminalization further exacerbates vulnerabilities across multiple groups [11,39]. Laws related to HIV exposure may deter individuals from seeking testing and treatment and hinder open communication with healthcare providers [47]. Similarly, the criminalization of sex work contributes to unsafe working conditions, increased violence, and reduced access to prevention services, with evidence showing that criminalizing clients can negatively affect condom use and safety [4,10]. For PWID, criminalization discourages service uptake, reinforces marginalization, and impedes harm reduction efforts [48,49]. These structural barriers are compounded by inconsistencies in how legal obligations are communicated [19], including variable guidance from healthcare providers, which may undermine trust and engagement in care. Together, these findings underscore the importance of coherent, rights-based policy approaches aligned with public health principles. Rights-based approaches and the institutionalization of national human rights could improve access to HIV services and promote equitable treatment for key populations [40]. Civil society and non-governmental organizations also play a crucial role, drawing on community-level experience and access to marginalized groups to support HIV prevention, treatment, and care [50,51].

These findings are consistent with existing evidence from prior studies and reports developed by relevant organizations [11,39,40,52], and have important implications for policy design and health systems. Greater clarity and consistency in defining and prioritizing key populations are needed to ensure more inclusive and targeted policy frameworks. Health systems should address barriers faced by key and priority populations as well as enhance incentives and facilitating policies to drive uptake of prevention, testing, treatment, and care. At the same time, variation across countries presents an opportunity for cross-country learning to identify effective approaches and inform more equitable strategies [16,53]. This knowledge exchange is essential for supporting the successful implementation of policies that align with UNAIDS goals and ultimately contribute to the global fight against HIV/AIDS.

### Limitations

This study provides a comprehensive and structured mapping of policies related to HIV key populations across multiple countries, offering valuable insights into policy variation and gaps. However, the limitations must be acknowledged, particularly regarding access to information. Some relevant policy documents may not be available online or may have been excluded based on their year of publication but still be applicable. Therefore, this study is a review of documented policies and legislation that impact HIV key populations, not a synthesis of all existing policies. In addition, although the search followed a structured approach based on predefined policy areas, it may not be entirely replicable due to differences in how policies are published and accessed across countries. The peer-reviewed literature search may not fully address the barriers and facilitators in each context as policy implementation can depend on specific socio-cultural, economic, and political settings that have not been discussed in published studies.

## Conclusion

There is variation across European countries regarding policies that impact HIV efforts for key populations. Even though national HIV plans and strategies focus on key groups due to their heightened vulnerability, disproportionate risk, and role in driving transmission dynamics, there are still direct and indirect barriers that render reaching these populations a difficult task. Cross-country learnings regarding effective and equitable policies and strategies to support policy implementation is recommended. It is imperative that health systems address barriers faced by key and priority populations to achieve the UNAIDS targets.

## Supporting information

S1 File Appendix I. Selection of sampled countries. Table S1.1. Criteria for selection of countries, final sample highlighted, Table S1.2. Data per domain and country. Appendix II. Targeted policy search-Table S2. Policy areas, full description. Appendix III. Peer-reviewed literature- Table S3. Search queries for Literature Review. Appendix IV. Results from peer-reviewed literature search- Table S4. Studies included in the analysis. Appendix V. Complete dataset of policies- Table S5. Complete dataset of policies found.
(DOCX)
