# Supplementary material for: HIV-relevant policies in European countries: A comprehensive landscape of policies hindering or facilitating access to HIV prevention, testing, treatment, and care of key populations
Source: PLoS One. 2026 Jun 8;21(6):e0350099. doi: 10.1371/journal.pone.0350099 (PMC13245746; doi:10.1371/journal.pone.0350099)
Supplement: S1 File — Table S1.1. Criteria for selection of countries, final sample highlighted, Table S1.2. Data per domain and country. Appendix II. Targeted policy search-Table S2. Policy areas, full description. Appendix III. Peer-reviewed literature- Table S3. Search queries for Literature Review. Appendix IV. Results from peer-reviewed literature search- Table S4. Studies included in the analysis. Appendix V. Complete dataset of policies- Table S5. Complete dataset of policies found. (DOCX) [file pone.0350099.s001.docx]

**THE IMPACT OF HIV-RELEVANT POLICIES ON KEY POPULATIONS IN EUROPEAN COUNTRIES: A COMPREHENSIVE LANDSCAPE OF POLICIES ACROSS THE CONTINUUM OF CARE**

**Supplementary Material**

**Table of contents**

[Appendix I. Selection of sampled countries	2](#_Toc211610515)

[Table S1.1. Criteria for selection of countries, final sample highlighted	4](#_Toc211610516)

[Table S1.2. Data per domain and country	6](#_Toc211610517)

[Appendix II. Targeted policy search	8](#_Toc211610518)

[Table S2. Policy areas, full description	8](#_Toc211610519)

[Appendix III. Peer-reviewed literature	18](#_Toc211610520)

[Table S3. Search queries for Literature Review	18](#_Toc211610521)

[Appendix IV. Complete dataset of policies	19](#_Toc211610522)

[Table S4. Complete dataset of policies found	19](#_Toc211610523)

[Appendix V. Results from peer-reviewed literature search	107](#_Toc211610524)

[Table S5. Studies included in the analysis	107](#_Toc211610525)

[References	112](#_Toc211610526)

# Appendix I. Selection of sampled countries

The following minimum criteria was defined to safeguard variation of sample:

Geopolitics

- ECDC Analysis of WHO/Europe region: At least five countries of West, two from Centre, two from East subregion. Source: ECDC [1]
- UN geoscheme: At least two countries of each subregion (Eastern Europe, Northern Europe, Southern Europe, Western Europe).

Income level

- Income level: At least three countries at each level of income (classified according to tercile values among the initial sample of countries). For each subregion (West, Centre, and East), there must be at least one country at each level of income when possible. Source: World bank [2]

Social acceptance of and receptivity towards key populations

- Discrimination: At least three countries at each level of discrimination when possible (classified according to tercile values among the initial sample of countries). Source: Equaldex [3]
- Regulations on sex work: At least three countries that prohibit sex work when possible. Source: European Parliament [4]

Health system type and key population access to health systems

- Institutional features of health systems: At least three countries classified as Bismarck and three countries classified as Beveridge. Sources: Ferreira et al. 2018 [5] and Gaeta et al. 2017 [6]
- Coverage of essential health services: At least four countries at each level of coverage (classified according to median values among the initial sample of countries). WHO [7]

Levels of progress towards achieving UNAIDS targets progress

- UNAIDS 95-95-95 targets: At least one country that did not reach any target; at least one country that reached two or more targets. The countries with the best and worst reported progress should be included. Marsh et al. 2019 [8] and UNAIDS 2021 [9]

Levels of progress towards achieving UNAIDS targets progress

- Prevalence and Incidence of HIV. Source: GBD [10]

The following approach was used.

1. Select the five countries with the highest burden of HIV in terms of HIV incidence per 100,000 people, as these represent countries in the region facing challenges regarding HIV prevention.

2. Select the five countries with the highest burden of HIV in terms of HIV prevalence in absolute numbers, as these represent the countries in the region most impacted or most affected by the HIV epidemic. Do not select countries already included in the first criteria.

3. Verify if the criteria for levels of progress towards achieving UNAIDS targets are fulfilled. If not, add the countries with the best and worst reported progress.

4. Verify if the criteria geopolitics are fulfilled. If not, include countries from the underrepresented categories according to highest burden of HIV- prevalence of HIV.

5. Verify if the criteria for social acceptance of and receptivity towards key populations are fulfilled. If not, add at least one country from the underrepresented categories according to burden of HIV- incidence of HIV.

6. Verify if the criteria health system type and key population access to health systems are fulfilled. If not, add at least one country from the underrepresented categories according to burden of HIV- prevalence of HIV.

7. Verify if the criteria income level is fulfilled. If not, add at least one country from the underrepresented categories according to burden of HIV- prevalence of HIV.

Following this approach, the 15 countries selected for the study were Belgium, Czech Republic, Estonia, France, Germany, Italy, Latvia, Netherlands, Poland, Portugal, Romania, Spain, Sweden, Switzerland, United Kingdom.

Some countries in Europe have implemented decentralized government and health systems, where decision-making and healthcare management often takes place at the regional or local levels rather than a central national level. To ensure the study accounts for this variation, a subnational region from decentralized countries was also selected. Policies enacted at the subnational level and peer-review papers for the following six subnational regions were also collected: Flanders (Belgium), Berlin (Germany), Lombardi (Italy), Catalonia (Spain), Vaud (Switzerland), and England (United Kingdom).

## Table S1.1. Criteria for selection of countries, final sample highlighted

| **Country** | **Geopolitics** | | **Income level** | **Social acceptance of and receptivity towards key populations** | | **Health system type and key population access to health systems** | | **UNAIDS targets** | **Burden of HIV** | **Burden of HIV** |
| --- | --- | --- | --- | --- | --- | --- | --- | --- | --- | --- |
|  | *WHO Europe Region* | *UN geoscheme* | *Income level* | *Public attitudes towards LGBT people* | *Regulations on sex work* | *Typology of health system* | *Coverage of essential health services* | *Levels of progress towards achieving UNAIDS’s targets* | *HIV Incidence (per 100,000 people)* | *HIV Prevalence (absolute numbers)* |
| Austria | West | Western Europe | Higher income | Medium discrimination | Legal, regulated | Mixed | Higher coverage |  | 7,21 | 17.405 |
| Belgium^2^ | West | Western Europe | Medium income | Lower discrimination | Legal, unregulated | Bismarck | Higher coverage |  | 7,15 | 23.814 |
| Bulgaria | Centre | Eastern Europe | Lower income | Higher discrimination | Legal, unregulated | Mixed | Lower coverage | No targets achieved | 1,45 | 1.776 |
| Croatia | Centre | Southern Europe | Lower income | Higher discrimination | Prohibit, punish the sex worker | Mixed | Lower coverage | No targets achieved | 0,91 | 901 |
| Cyprus | Centre | Western Asia | Medium income | Higher discrimination | Legal, unregulated | Beveridge | Lower coverage |  | 0,33 | 193 |
| Czech Republic^7^ | Centre | Eastern Europe | Medium income | Medium discrimination | Legal, unregulated | Bismarck | Lower coverage | 1 target achieved | 0,19 | 1.671 |
| Denmark | West | Northern Europe | Higher income | Lower discrimination | Legal, unregulated | Beveridge | Higher coverage | 2 targets achieved | 5,33 | 6.519 |
| Estonia^4^ | East | Northern Europe | Lower income | Higher discrimination | Legal, unregulated | Bismarck | Lower coverage | No targets achieved | 0,23 | 3.213 |
| Finland | West | Northern Europe | Higher income | Medium discrimination | Legal, unregulated | Beveridge | Higher coverage | No targets achieved | 0,65 | 1.362 |
| France^2^ | West | Western Europe | Medium income | Medium discrimination | Prohibit, punish the client | Bismarck | Higher coverage | No targets achieved | 5,39 | 102.793 |
| Germany^2^ | West | Western Europe | Higher income | Lower discrimination | Legal, regulated | Bismarck | Higher coverage | 1 target achieved | 3,59 | 80.179 |
| Greece | West | Southern Europe | Lower income | Higher discrimination | Legal, regulated | Mixed | Lower coverage | No targets achieved | 0,83 | 4.407 |
| Hungary | Centre | Eastern Europe | Lower income | Higher discrimination | Legal, regulated | Bismarck | Lower coverage | No targets achieved | 1,12 | 2.069 |
| Iceland | West | Northern Europe | Higher income | Lower discrimination |  | Beveridge | Higher coverage | 2 targets achieved | 2,22 | 193 |
| Ireland | West | Northern Europe | Higher income | Medium discrimination | Prohibit, punish the client | Beveridge | Higher coverage | 1 target achieved | 1,26 | 2.224 |
| Italy^1^ | West | Southern Europe | Medium income | Medium discrimination | Legal, unregulated | Beveridge | Higher coverage | No targets achieved | 10,28 | 96.495 |
| Latvia^1^ | East | Northern Europe | Lower income | Higher discrimination | Legal, regulated | Beveridge | Lower coverage |  | 8,26 | 3.795 |
| Lithuania | East | Northern Europe | Lower income | Higher discrimination | Prohibit, punish both | Bismarck | Lower coverage |  | 2,33 | 1.879 |
| Luxembourg | West | Western Europe | Higher income | Medium discrimination | Legal, unregulated | Bismarck | Higher coverage | No targets achieved | 4,03 | 558 |
| Malta | West | Southern Europe | Medium income | Medium discrimination | Legal, unregulated | Beveridge | Lower coverage |  | 1,19 | 405 |
| Netherlands^2^ | West | Western Europe | Higher income | Lower discrimination | Legal, regulated | Bismarck | Higher coverage | No targets achieved | 2,92 | 21.048 |
| Norway | West | Northern Europe | Higher income | Lower discrimination |  | Beveridge | Higher coverage | No targets achieved | 4,92 | 3.454 |
| Poland^4^ | Centre | Eastern Europe | Lower income | Medium discrimination | Legal, unregulated | Bismarck | Lower coverage |  | 0,77 | 15.674 |
| Portugal^1^ | West | Southern Europe | Lower income | Medium discrimination | Legal, unregulated | Beveridge | Higher coverage | 1 target achieved | 18,83 | 39.360 |
| Romania^3^ | Centre | Eastern Europe | Lower income | Higher discrimination | Prohibit, punish the sex worker | Bismarck | Lower coverage | No targets achieved | 1,31 | 14.583 |
| Slovakia | Centre | Eastern Europe | Lower income | Higher discrimination | Legal, unregulated | Bismarck | Lower coverage | No targets achieved | 0,18 | 346 |
| Slovenia | Centre | Southern Europe | Medium income | Medium discrimination | Legal, unregulated | Bismarck | Lower coverage |  | 0,38 | 274 |
| Spain^1^ | West | Southern Europe | Medium income | Lower discrimination | Legal, unregulated | Beveridge | Higher coverage | No targets achieved | 14,98 | 103.330 |
| Sweden^5^ | West | Northern Europe | Higher income | Lower discrimination | Prohibit, punish the client | Beveridge | Higher coverage | 2 targets achieved | 2,79 | 5.099 |
| Switzerland^1^ | West | Western Europe | Higher income | Lower discrimination |  | Bismarck | Higher coverage | 2 targets achieved | 12,29 | 22.508 |
| United Kingdom^2^ | West | Northern Europe | Medium income | Lower discrimination |  | Beveridge | Higher coverage | 2 targets achieved | 3,16 | 131.957 |

1. Countries added on step 1

2. Countries added on step 2

3. Countries added on step 3

4. Countries added on step 4

5. Countries added on step 5

6. Countries added on step 6

7. Countries added on step 7

## Table S1.2. Data per domain and country

| **Country** | **Geopolitics** | | **Income level** | **Social acceptance of and receptivity towards key populations** | | **Health system type and key population access to health systems** | | **UNAIDS targets** | **Burden of HIV** | **Burden of HIV** |
| --- | --- | --- | --- | --- | --- | --- | --- | --- | --- | --- |
|  | *WHO Europe Region* | *UN geoscheme* | *Income level (2021 or the latest year available)* | *Public attitudes towards LGBT people (0-100). 2021* | *Regulations on sex work* | *Typology of health system* | *Universal Health Service Coverage Index (2019 or latest year available)* | *% People living with HIV who know their status; % People who are on ART among those who know their HIV status; % People with suppressed viral load among those on ART; Year* | *HIV Incidence (per 100,000 people)* | *HIV Prevalence (absolute numbers)* |
| Austria | West | Western Europe | 51.857,69 | 57 | Legal, regulated | Mixed | 82 | ...; 93 [82->95]; 86 [76->95]. Year 2016 | 7,21 | 17.405 |
| Belgium | West | Western Europe | 48.769,73 | 67 | Legal, unregulated | Bismarck | 85 | ...; ...; .... | 7,15 | 23.814 |
| Bulgaria | Centre | Eastern Europe | 22.379,09 | 25 | Legal, unregulated | Mixed | 70 | 83 [71->95]; 50 [43-58]; 94 [80->95]. Year 2018 | 1,45 | 1.776 |
| Croatia | Centre | Southern Europe | 27.076,98 | 33 | Prohibit, punish the sex worker | Mixed | 73 | 84 [69->95]; 75 [61-89]; 73 [60-87]. Year 2020 | 0,91 | 901 |
| Cyprus | Centre | Western Asia | 38.816,09 | 33 | Legal, unregulated | Beveridge | 79 | ...; ...; .... | 0,33 | 193 |
| Czech Republic | Centre | Eastern Europe | 38.511,27 | 59 | Legal, unregulated | Bismarck | 78 | 61 [51-69]; 74 [63-85]; .... Year 2018 | 0,19 | 1.671 |
| Denmark | West | Northern Europe | 55.819,91 | 73 | Legal, unregulated | Beveridge | 85 | 90 [81->95]; 95 [85->95]; >95 [89->95]. Year 2016 | 5,33 | 6.519 |
| Estonia | East | Northern Europe | 35.257,16 | 36 | Legal, unregulated | Bismarck | 78 | 83 [74-92]; 71 [63-79]; 90 [80->95]. Year 2018 | 0,23 | 3.213 |
| Finland | West | Northern Europe | 47.154,43 | 63 | Legal, unregulated | Beveridge | 83 | 91 [72->95]; 84 [66->95]; 88 [70->95]. Year 2018 | 0,65 | 1.362 |
| France | West | Western Europe | 42.320,52 | 63 | Prohibit, punish the client | Bismarck | 84 | 88 [74->95]; 92 [77->95]; 93 [78->95]. Year 2016 | 5,39 | 102.793 |
| Germany | West | Western Europe | 51.423,23 | 70 | Legal, regulated | Bismarck | 86 | 87 [71->95]; 90 [74->95]; 95 [77->95]. Year 2017 | 3,59 | 80.179 |
| Greece | West | Southern Europe | 27.072,62 | 38 | Legal, regulated | Mixed | 78 | 82 [74-89]; 67 [61-72]; .... Year 2020 | 0,83 | 4.407 |
| Hungary | Centre | Eastern Europe | 31.098,40 | 39 | Legal, regulated | Bismarck | 73 | 90 [78->95]; 62 [54-70]; .... Year 2018 | 1,12 | 2.069 |
| Iceland | West | Northern Europe | 52.375,68 | 86 |  | Beveridge | 87 | 80 [72-88]; >95 [88->95]; 95 [85->95]. Year 2018 | 2,22 | 193 |
| Ireland | West | Northern Europe | 90.789,22 | 60 | Prohibit, punish the client | Beveridge | 83 | 90 [78->95]; 89 [77->95]; 95 [82->95]. Year 2018 | 1,26 | 2.224 |
| Italy | West | Southern Europe | 39.073,41 | 54 | Legal, unregulated | Beveridge | 83 | 92 [79->95]; 87 [75->95]; 87 [75->95]. Year 2016 | 10,28 | 96.495 |
| Latvia | East | Northern Europe | 30.100,27 | 27 | Legal, regulated | Beveridge | 72 | ...; ...; .... | 8,26 | 3.795 |
| Lithuania | East | Northern Europe | 37.107,06 | 26 | Prohibit, punish both | Bismarck | 70 | ...; ...; .... | 2,33 | 1.879 |
| Luxembourg | West | Western Europe | 112.557,31 | 63 | Legal, unregulated | Bismarck | 86 | 86 [75->95]; 89 [78->95]; 89 [78->95]. Year 2018 | 4,03 | 558 |
| Malta | West | Southern Europe | 39.979,70 | 65 | Legal, unregulated | Beveridge | 81 | ...; ...; .... | 1,19 | 405 |
| Netherlands | West | Western Europe | 54.324,38 | 73 | Legal, regulated | Bismarck | 86 | 90 [81->95]; 93 [84->95]; 94 [85->95]. Year 2017 | 2,92 | 21.048 |
| Norway | West | Northern Europe | 63.548,00 | 81 |  | Beveridge | 86 | …; 80 [69-91]; 79 [68-90]. Year 2020 | 4,92 | 3.454 |
| Poland | Centre | Eastern Europe | 32.398,71 | 40 | Legal, unregulated | Bismarck | 74 | ...; ...; .... | 0,77 | 15.674 |
| Portugal | West | Southern Europe | 31.961,78 | 50 | Legal, unregulated | Beveridge | 84 | 89 [78->95]; >95 [85->95]; .... Year 2017 | 18,83 | 39.360 |
| Romania | Centre | Eastern Europe | 28.870,93 | 25 | Prohibit, punish the sex worker | Bismarck | 71 | 87 [3->95]; 66 [40-84]; 60 [36-76]. Year 2020 | 1,31 | 14.583 |
| Slovakia | Centre | Eastern Europe | 30.509,77 | 38 | Legal, unregulated | Bismarck | 77 | 69 [52->95]; 78 [59->95]; 86 [65->95]. Year 2018 | 0,18 | 346 |
| Slovenia | Centre | Southern Europe | 37.050,55 | 48 | Legal, unregulated | Bismarck | 80 | ...; ...; .... | 0,38 | 274 |
| Spain | West | Southern Europe | 36.210,88 | 71 | Legal, unregulated | Beveridge | 86 | 86 [76->95]; 93 [82->95]; 90 [79->95]. Year 2016 | 14,98 | 103.330 |
| Sweden | West | Northern Europe | 50.923,11 | 76 | Prohibit, punish the client | Beveridge | 87 | ...; >95 [88->95]; 95 [87->95]. Year 2017 | 2,79 | 5.099 |
| Switzerland | West | Western Europe | 68.755,44 | 69 |  | Bismarck | 87 | ...; >95 [85->95]; >95 [87->95]. Year 2018 | 12,29 | 22.508 |
| United Kingdom | West | Northern Europe | 42.675,78 | 75 |  | Beveridge | 88 | 92 [77->95]; >95 [82->95]; >95 [81->95]. Year 2017 | 3,16 | 131.957 |

# Appendix II. Targeted policy search

## Table S2. Policy areas, full description

| **Definitions** | | **Question** | **Adopted** | **Partially adopted** | **Not adopted** | **Sources** |
| --- | --- | --- | --- | --- | --- | --- |
| **A. Treatment and care** | | | | | | |
| A1 | 1. Treatment Initiation regardless of CD4 count | Are all people living with HIV, regardless of CD4 count, eligible to start HIV treatment in national policy? | All people living with HIV, regardless of CD4 count, are eligible to start HIV treatment according to national guidelines |  | According to national guidelines, only people whose CD4 count falls below a certain threshold are eligible to start ART | WHO, Guideline on When to Start ART and PrEP, 2015; WHO, Consolidated Guidelines on the Use of Antiretroviral Drugs for Treating and Preventing HIV Infection, 2016. |
| A2 | 2. Test and treat (ART started as soon as possible) | Is the option to start treatment as soon as possible after HIV diagnosis included in national policy? | National guidelines explicitly allow for start as soon as possible |  | National guidelines do not explicitly allow for start as soon as possible | WHO, Consolidated Guidelines on the Use of Antiretroviral Drugs for Treating and Preventing HIV Infection, 2021; WHO Consolidated Guideline on HIV Prevention, Early Infant Diagnosis and Antiretroviral Initiation & Monitoring, 2021; WHO, Guidelines for Managing Advanced HIV Disease and Rapid Initiation of ART, 2017. |
| A3 | 3. Treatment regimen | Are up to date first-line ARV regimens aligned with international recommendations included in national HIV policy? | The first-line ARV regimen included in national HIV treatment guidelines aligns with international recommendations |  | The first-line ARV regimen included in national HIV treatment guidelines does not align with international recommendations | WHO, Consolidated Guidelines on the Use of Antiretroviral Drugs for Treating and Preventing HIV Infection, 2021; WHO, Update of Recommendations on First- and Second-Line ARVs, 2019; WHO, Updated Recommendations on First-Line & Second-Line ARVs and PEP and Recommendations on Early Infant Diagnosis of HIV, 2018; WHO, Consolidated Guidelines on the Use of Antiretroviral Drugs for Treating and Preventing HIV Infection, 2016. |
| A4 | 4. Differentiated service delivery | Do national HIV treatment policies include multiple options for differentiated HIV treatment services? | National policies include multiple options for differentiated service delivery, including community ART distribution and maximum flexibility for clinic visit and multi-month dispensing | National policies include at least one option for differentiated service delivery (i.e., community ART distribution, reduced clinic visits, or multi-month dispensing) | National policies do not include options for differentiated HIV treatment services | WHO, Updated Recommendations for Service Delivery for Treatment & Care of PLHIV, April 2021; WHO, Updated Recommendations on HIV Prevention, Infant Diagnosis, Antiretroviral Initiation and Monitoring, March 2021; WHO, CDC, PEPFAR, USAID & IAS, Key Considerations for Differentiated ART Delivery for Specific Populations, 2017; WHO, Consolidated Guidelines on the Use of Antiretroviral Drugs for Treating and Preventing HIV Infection, 2016. |
| A5 | 5. Viral load testing | Is viral load monitoring at least once per year provided for in national policy? | National policy provides for viral load monitoring at least once per year | National policy provides for viral load monitoring frequently, but not clear if at least once per year | National policy does not provide for viral load monitoring at least once per year | WHO, What's New in Treatment Monitoring: Viral Load & CD4 Testing, 2017; WHO, Consolidated Guidelines on the Use of Antiretroviral Drugs for Treating and Preventing HIV Infection, 2016. |
| A6 | 6. Pediatric diagnosis & treatment | Are national pediatric testing and treatment policies aligned with international recommendations? | National guidelines for pediatric testing and treatment align with international recommendations | National guidelines for pediatric testing align with international recommendations but national guidelines for pediatric treatment do not; or national guidelines for pediatric treatment align with international recommendations but national guidelines for pediatric testing do not | National guidelines for pediatric testing and/or treatment do not align with international recommendations | WHO, Consolidated Guidelines on the Use of Antiretroviral Drugs for Treating and Preventing HIV Infection, 2021; WHO, Updated Recommendations on HIV Prevention, Infant Diagnosis, Antiretroviral Initiation and Monitoring, March 2021; WHO, Updated Recommendations on First-Line & Second-Line ARVs and PEP and Recommendations on Early Infant Diagnosis of HIV, 2018; WHO & IATT, 2018 Optimal Formulary and Limited-Use List for Paediatric ARVs, 2018; WHO, Novel Point-of-Care Tools for Early Infant Diagnosis of HIV, 2017; WHO, What's New in Infant Diagnosis, 2015. |
| A7 | 7. Migrants' access to healthcare | Are HIV services and primary healthcare available to all migrants under the same conditions as citizens under national policy? | National laws/policy makes primary healthcare and HIV services accessible to all migrants under the same conditions as citizens | National laws/policy makes either primary healthcare or HIV services accessible to all migrants under the same conditions as citizens | National laws/policy restricts access to primary healthcare and HIV services for some or all migrants | Global Commission on HIV and the Law, Risks, Rights & Health Supplement, 2018; United Nations General Assembly, New York Declaration for Refugees and Migrants, 2016; Global Commission on HIV and the Law, Risks, Rights & Health, 2012. |
| A8 | 8. Access to ART for incarcerated people | Are HIV services and primary healthcare available to incarcerated people as a national policy? | National laws/policy makes primary healthcare and HIV services accessible to all incarcerated persons. | National laws/policy makes either primary healthcare or HIV services accessible to incarcerated persons | National laws/policy restricts access to primary healthcare and HIV services for incarcerated persons | Latvia pilot. |
| A9 | 9. TB diagnostics for people with HIV | Are rapid diagnostic tests (e.g., rapid molecular diagnostics, TB-LAM) aligned with WHO recommendations used as the initial diagnostic test for TB in PLHIV under national policy? | National policy includes the use of WHO-approved rapid diagnostic tests (e.g., rapid molecular diagnostics, TB LAM) as the initial diagnostic test for TB in PLHIV |  | National policy does not include the use of WHO-approved rapid diagnostic tests (e.g., rapid molecular diagnostic, TB LAM) as the initial diagnostic test for TB in PLHIV | WHO, Consolidated Guidelines on Tuberculosis-Rapid Diagnostics for TB Detection, 2020; WHO, Lateral flow urine lipoarabinomannan assay (LF-LAM) for the diagnosis of active tuberculosis in people living with HIV, 2019; WHO, Xpert MTB/RIF assay for the diagnosis of pulmonary and extrapulmonary TB in adults and children, 2013 |
| A10 | 10. HIV treatment guidelines for adults | Are there national HIV treatment guidelines for adults available? | Guideline was identified |  | Guideline not identified | HIV Policy Lab [11]. |
| A11 | 11. HIV treatment guidelines for women and newborns | Are there national HIV treatment guidelines for pregnant women and newborns? | Guideline was identified |  | Guideline not identified | HIV Policy Lab [11]. |
| A12 | 12. Support services for PLHIV | Are there services to help people with HIV navigate the healthcare and welfare system so they can access services and programs aimed at them? | Support services are available | There are support services either for healthcare or for welfare programs, but not both | No evidence of support services for people with HIV | Latvia pilot. |
| **B. Testing and prevention** | | | | | | |
| B1 | 1. Testing guidelines/standards | Are testing guidelines/standards available in the country? | Testing guidelines or standards were identified |  | No evidence of existence of testing guidelines/standards | HIV Policy Lab [11]. |
| B2 | 2. Self-testing | Is self-testing approved in national policy? | Self-testing is approved in national policy |  | Self-testing is not approved in national policy | WHO, Guidelines on HIV Self-Testing and Partner Notification, 2016. |
| B3 | 3. Partner notification/Index testing | Is index testing/partner notification, with robust protections for patient confidentiality, provided for in national policy? | National guidelines incorporate index testing/partner notification while prioritizing robust protections for patient confidentiality | National guidelines incorporate index testing/partner notification but do not prioritize robust protections for patient confidentiality | National guidelines do not include index testing/partner notification or does not include robust protections against disclosure of HIV status without patients' consent | UNAIDS, Fast-Track and Human Rights, 2017; WHO, Guidelines on HIV Self-Testing and Partner Notification, 2016; UNAIDS, Criminalization of HIV Transmission Policy Brief, 2008. |
| B4 | 4. Compulsory testing (including travel-related) | Is compulsory HIV testing prohibited under national law? | National law/policy prohibits compulsory HIV testing, except in cases of blood/organ donation or with a court order |  | National law/policy does not prohibit compulsory HIV testing | WHO & UNAIDS, Statement on HIV Testing Services, 2017. |
| B5 | 5. Age restrictions on testing & treatment | Can adolescents access HIV testing and treatment without parental consent under national policy? | National law/policy does not require adolescents (≥12 years) to obtain parental/guardian consent to access HIV testing and/or treatment |  | National law/policy requires adolescents to obtain parental/guardian consent to access HIV testing and/or treatment | WHO, Consolidated Guidelines on the Use of Antiretroviral Drugs for Treating and Preventing HIV Infection, 2021; Joint United Nations statement on ending discrimination in health care settings, 2017; UNAIDS, Prevention Gap Report, 2016; UN, Report of the Special Rapporteur on the right of everyone to the enjoyment of the highest attainable standard of physical and mental health, 2016; WHO, HIV and Adolescents: Guidance for HIV Testing and Counselling and Care for Adolescents Living with HIV, 2013; Global Commission on HIV and the Law, Risks, Rights & Health, 2012. |
| B6 | 6. PrEP with wide/narrow eligibility criteria | Are all people/populations at substantial risk of HIV infection eligible for PrEP under national policy and have technologies for pre-exposure prophylaxis (PrEP) received national regulatory approval? | National policy makes PrEP available to people/populations at substantial risk and at least one PrEP technology has regulatory approval | National policy makes PrEP available to people/populations at substantial risk, but no PrEP technologies have received regulatory approval; or at least one PrEP technology has regulatory approval but national policy on PrEP eligibility excludes some people/populations at substantial risk | PrEP is not available under national policy and no PrEP technology has received regulatory approval | WHO, Consolidated Guidelines on the Use of Antiretroviral Drugs for Treating and Preventing HIV Infection, 2021; WHO, Guideline on When to Start ART and PrEP, 2015. |
| B7 | 7. Harm reduction | Does national law and HIV policy incorporate key harm reduction strategies, including avoidance of criminalizing syringe possession? | Harm reduction services are included in national policy and service packages for people who inject drugs and syringe possession is not criminalized | Either harm reduction services are included in national policy and service packages for people who inject drugs or syringe possession is not criminalized, but not both | Harm reduction services are not included in national policy and service packages for people who inject drugs and syringe possession is criminalized | UNAIDS, Fast-Track and human rights, 2017; Global Commission on HIV and the Law, Risks, Rights & Health, 2012; United Nations, Political Declaration on HIV and AIDS, 2011. |
| B8 | 8. Comprehensive sexuality education | Is comprehensive sexuality education required in primary and secondary schools under national policy? | National policy requires that a comprehensive sexuality education curriculum meeting international standards be taught in primary and secondary schools |  | National policy does not require that a comprehensive sexuality education curriculum meeting international standards be taught in primary & secondary schools | UNESCO, International Technical Guidance on Sexuality Education, 2018; UNAIDS, Fast-Track Strategy 2016-2021. |
| B9 | 9. Prevention strategy/policy/service for sex workers and their clients | Is there an explicit strategy/policy/service for prevention targeted at sex workers? | An official strategy/policy/service was identified |  | No evidence of existence of an official prevention strategy/policy/service for sex workers | HIV Policy Lab [11]. |
| B10 | 10. Prevention strategy/policy/service for MSM | Is there an explicit strategy/policy/service for prevention targeted at MSM? | An official strategy/policy/service was identified. |  | No evidence of existence of an official prevention strategy/policy/service for MSM | HIV Policy Lab [11]. |
| B11 | 11. Prevention services for incarcerated people and people in other closed settings | Are both condoms/lubricants and syringe access/exchange programs available to incarcerated people as a matter of policy? | Both condoms/lubricants and syringe access/exchange programs are available to incarcerated people | Either condoms/lubricants or syringe access/exchange programs are available to incarcerated people, but not both | Neither condoms/lubricants nor syringe access/exchange programs are available to incarcerated people | Global Commission on HIV and the Law, Risks, Rights & Health, 2012. |
| B12 | 12. Sexual and reproductive health law/regulation (includes/excludes) HIV prevention | Is there a sexual and reproductive health law/regulation that includes HIV prevention? | There is a law/regulation on sexual and reproductive health, and it includes HIV prevention | There is a law/regulation on sexual and reproductive health, but it does not include HIV prevention | No evidence of existence of a sexual and reproductive health law or regulation | Latvia pilot. |
| B13 | 13. General health promotion/disease prevention plan/strategy (including/excluding) HIV prevention | Is there a general health promotion or disease prevention plan or strategy and it includes HIV prevention? | There is a health promotion/disease prevention plan or strategy, and it includes HIV prevention | There is a health promotion/disease prevention plan or strategy, but it does not include HIV prevention | No evidence of existence of a health promotion/disease prevention plan or strategy | Latvia pilot. |
| B14 | 14. Sell/provision of condoms in place where key populations occur | Is there a law, regulation, program, or strategy for the sell or provision (or the prohibition) of condoms in saunas, bathroom, cinemas, nightclubs, hotels/motels? | There is a law, regulation, program, or strategy for the sell or provision of condoms in saunas, bathroom, cinemas, nightclubs, hotels/motels |  | There is a law or regulation prohibiting the sell or provision of condoms in saunas, bathroom, cinemas, nightclubs, hotels/motels | HIV Policy Lab [11]. |
| **C. Normative and Structural Characteristics** | | | | | | |
| C1 | 1. Non-criminalization/criminalization of same-sex relations | Does national law/policy refrain from criminalizing and prosecuting people for consensual same-sex sexual acts? | National law avoids criminalizing consensual same-sex sex acts and there are no reports of people being prosecuted for consensual same-sex sex acts in recent years | National law criminalizes consensual same-sex sex acts but there are no reports of people being prosecuted in recent years; or there are reports of people being prosecuted in recent years even though consensual same-sex sex acts are not criminalized under national law | National law criminalizes consensual same-sex sex acts and there are reports of people being prosecuted in recent years | UNAIDS Global AIDS Strategy 2021-2016; UNAIDS, Fast-Track and human rights, 2017; Global Commission on HIV and the Law, Risks, Rights & Health, 2012. |
| C2 | 2. Sex work non-criminalization/criminalization | Does national law avoid criminalizing sex work (buying, selling, and organizing of sex work)? | Buying, selling, and organizing of sex work are not crimes under national laws and country does not impose punitive regulations on sex work | Buying or selling sex are not crimes under national law, however the management/organization/facilitation of sex work is a crime and in some countries is used against sex workers; or national law operates a system of ‘legalization’ which imposes punitive or overly restrictive regulations on sex work | Buying or selling of sex are crimes, or other activities associated with the buying or selling of sex are crimes | UNAIDS Global AIDS Strategy 2021-2016; UNAIDS, Fast-Track and human rights, 2017; Global Commission on HIV and the Law, Risks, Rights & Health, 2012. |
| C3 | 3. Drug use non-criminalization/criminalization | Does national law refrain from criminalizing personal drug use/possession? | National law refrains from criminalizing drug use or possession of drugs, including opioids, for personal consumption | National law refrains from criminalizing drug use or possession for personal consumption, but may still impose punitive civil sanctions | National law criminalizes drug use or possession of drugs for personal consumption | UNAIDS Global AIDS Strategy 2021-2016; UNAIDS, Fast-Track and human rights, 2017; Global Commission on HIV and the Law, Risks, Rights & Health, 2012. |
| C4 | 4. Drug and addictions strategy | Is there a national drug and addictions plan or strategy? | There is a national drug and addictions plan or strategy |  | No evidence of existence of national drug and addictions plan or strategy | HIV Policy Lab [11]. |
| C5 | 5. Syringe services programs (SSPs) | Is there a national syringe services program or policy? | There is a national syringe services program or policy |  | No evidence of existence of national syringe services program or policy | HIV Policy Lab [11]. |
| C6 | 6. Opioid substitution programs | Are opioid substitution (methadone treatment) programs available and free for addicts? | Opioid substitution programs are available an provided at no cost |  | No evidence that opioid substitution programs are available an provided at no cost | HIV Policy Lab [11]. |
| C7 | 7. HIV exposure non-criminalization/criminalization | Does national law refrain from criminalizing and prosecuting people for HIV exposure/ transmission? | National law does not criminalize non-intentional HIV exposure/transmission and there are no reports of people being arrested/prosecuted for HIV transmission in recent years | National law criminalizes non-intentional HIV exposure/transmission or there have reports of people being arrested/prosecuted for HIV transmission in recent years, even though the law does not explicitly criminalize it | National law criminalizes non-intentional HIV exposure/transmission and there are reports of people being arrested/prosecuted for it in recent years | UNAIDS Global AIDS Strategy 2021-2016; UNAIDS, Fast-Track Strategy 2016-2021; Global Commission on HIV and the Law, Risks, Rights & Health, 2012; UNAIDS, Criminalization of HIV Transmission Policy Brief, 2008. |
| C8 | 8. Non-discrimination protections (constitutional/laws/programs) | Do national/laws policies include protections from discrimination on a general basis? | There is legislation or a constitutional provision prohibiting discrimination on a general basis |  | No evidence of constitutional provisions or laws against discrimination | UNAIDS Global AIDS Strategy 2021-2016; Joint United Nations statement on ending discrimination in health care settings, 2017; UNAIDS, Fast-Track Strategy 2016-2021; Global Commission on HIV and the Law, Risks, Rights & Health, 2012. |
| C8-A | a) For people with HIV | Do national/laws policies include protections from discrimination on the basis of HIV status? | National laws protect people from discrimination, including healthcare and employment discrimination, based on HIV status |  | No evidence of national laws protecting people from discrimination, including employment discrimination, based on HIV status | UNAIDS Global AIDS Strategy 2021-2016; Joint United Nations statement on ending discrimination in health care settings, 2017; UNAIDS, Fast-Track Strategy 2016-2021; Global Commission on HIV and the Law, Risks, Rights & Health, 2012. |
| C8-B | b) For MSM | Do national/laws policies include protections from discrimination based on sexual preferences? | National laws protect people from discrimination, including healthcare and employment discrimination, based on sexual preferences |  | No evidence of national laws protecting people from discrimination, including employment discrimination, based on sexual preferences | UNAIDS Global AIDS Strategy 2021-2016; Joint United Nations statement on ending discrimination in health care settings, 2017; UNAIDS, Fast-Track Strategy 2016-2021; Global Commission on HIV and the Law, Risks, Rights & Health, 2012. |
| C8-C | c) For people who use drugs | Do national/laws policies include protections from discrimination related to addictions? | National laws protect people from discrimination, including healthcare and employment discrimination related to addictions |  | No evidence of national laws protecting people from discrimination, including employment discrimination related to addictions | UNAIDS Global AIDS Strategy 2021-2016; Joint United Nations statement on ending discrimination in health care settings, 2017; UNAIDS, Fast-Track Strategy 2016-2021; Global Commission on HIV and the Law, Risks, Rights & Health, 2012. |
| C8-D | d) For trans people | Do national/laws policies include protections from discrimination based on gender/sex change? | National laws protect people from discrimination, including healthcare and employment discrimination, based on gender/sex change |  | No evidence of national laws protecting people from discrimination, including employment discrimination, based on gender/sex change and provides for gender and name change in national identity documents | UNAIDS Global AIDS Strategy 2021-2016; Joint United Nations statement on ending discrimination in health care settings, 2017; UNAIDS, Fast-Track Strategy 2016-2021; Global Commission on HIV and the Law, Risks, Rights & Health, 2012. |
| C8-E | e) For migrants | Do national/laws policies include protections from discrimination against migrants/refugees? | National laws protect people from discrimination, including healthcare and employment discrimination for migrants/refugees |  | No evidence of national laws protecting people from discrimination, including employment discrimination for migrants/refugees | UNAIDS Global AIDS Strategy 2021-2016; Joint United Nations statement on ending discrimination in health care settings, 2017; UNAIDS, Fast-Track Strategy 2016-2021; Global Commission on HIV and the Law, Risks, Rights & Health, 2012. |
| C8-F | f) For people during or after incarceration | Do national/laws policies include protections from discrimination based on past incarceration? | National laws protect people from discrimination, including healthcare and employment discrimination, based on past incarceration |  | No evidence of national laws protecting people from discrimination, including employment discrimination, based on past incarceration | UNAIDS Global AIDS Strategy 2021-2016; Joint United Nations statement on ending discrimination in health care settings, 2017; UNAIDS, Fast-Track Strategy 2016-2021; Global Commission on HIV and the Law, Risks, Rights & Health, 2012. |
| C9 | 9. Policies on legal gender change | Do policies on legal gender change and provisions for gender/sex change, including name change? | National laws provide for gender and name change in national identity documents |  | National laws do not allow or do not provide for gender and name change in national identity documents | HIV Policy Lab [11]. |
| C10 | 10. National human rights institutions | Is there an independent national human rights institution to which violations can be reported? | The country has a national human rights institution that is fully compliant with the Paris principles | The country has a national human rights institution that is partially compliant with Paris principles | The country does not have a national human rights institution to which violations can be reported in compliance with the Paris principles | UNAIDS Global AIDS Strategy 2021-2016; United Nations, Principles relating to the Status of National Institutions (The Paris Principles), 1993. |
| C11 | 11. Constitutional right to health | Is there an enforceable right to health in the national constitution? | National constitution includes the right to health |  | National constitution does not include the right to health | WHO Constitution, 1948. |
| C12 | 12. Girls’ education | Is there a national policy in place to encourage secondary school retention among girls? | National policies strongly encourage secondary school retention among girls | National policies take limited steps to encourage secondary school retention among girls | The country lacks policies to encourage secondary school retention among girls | UNAIDS, Fast Track Strategy 2016-2021. |
| C13 | 13. Gender-based violence | Does the law explicitly address domestic violence with enforceable penalties? | Country has domestic violence legislation with enforceable penalties |  | Country does not have domestic violence legislation with enforceable penalties | UNAIDS Global AIDS Strategy 2021-2016; Global Commission on HIV and the Law, Risks, Rights & Health, 2012; United Nations, Political Declaration on HIV and AIDS, 2011. |
| C14 | 14. Civil society (NGO access to registration and funding) | Are NGOs/CSOs able to register, seek funding and operate freely under national law and is there a social contracting policy for financing NGOs/CSO-provided services? | There is a social contracting policy for financing NGOs/CSO-provided services and NGOs/CSOs (including those that work with key populations) can register, seek funding, and operate freely under national law | Either there is a social contracting policy in place or NGOs/CSOs (including those that work with key populations) can register, seek funding, and operate freely under national law, but not both | There is no social contracting policy for financing NGOs/CSO-provided services, nor are NGOs/CSOs (including those that work with key populations) able to register, seek funding, and operate freely under national law | Global Fund Sustainability Transition & Co-financing Policy, 2016; United Nations, Political Declaration on HIV and AIDS, 2011. |
| C15 | 15. Laws restricting entry, stay or residence of people with HIV | Are there any laws or regulations that restrict entry, stay or residence for people with HIV? | There are no restrictions against people with HIV | National law restricts either entry, or stay and residence | National law restricts entry, stay and residence for people with HIV | HIV Policy Lab [11]. |
| C16 | 16. Equal marriage legislation | Does national law permit marriage between people of the same sex? | National law permits same-sex marriage |  | National law excludes same-sex marriage | HIV Policy Lab [11] / Latvia pilot. |
| **D. Health Systems** | | | | | | |
| D1 | 1. Overall National HIV plan/strategy | Is there a current, overall national plan or strategy for the prevention and control of HIV? | A current national plan or strategy for the prevention of HIV was located | A national plan or strategy for the prevention of HIV was located, but it is not current | No evidence of a national plan or strategy for the prevention of HIV was located | HIV Policy Lab [11] / Latvia pilot. |
| D2 | 2. National HIV Monitoring and evaluation plan/strategy | Is there a current national plan or strategy for HIV monitoring and evaluation? | A current national plan for HIV monitoring an evaluation was located | A national plan or strategy HIV monitoring and evaluation was located, but it is not current | No evidence of a national plan or strategy for HIV monitoring and evaluation | HIV Policy Lab [11]. |
| D3 | 3. Task shifting (non-physician HCPs can initiate ART) | Are nurses or other non-physicians allowed to initiate HIV treatment under national policy? | National policy allows nurses or other non-physicians to initiate ART |  | National policy does not allow nurses or other non-physicians to initiate ART | WHO, CDC, PEPFAR, USAID & IAS, Key Considerations for Differentiated ART Delivery for Specific Populations, 2017 |
| D4 | 4. Health financing (dedicated allocation for HIV) | Does the national budget have a line-item for the HIV program? | A line-item in the country's budget provides an allocation for HIV prevention and control |  | No evidence of a dedicated allocation for HIV prevention and control | Adapted from WHO, UHC-Lessons to Guide Country Actions on Health Financing, 2019; Global Fund Sustainability Transition & Co-financing Policy, 2016. |
| D5 | 5. Universal health coverage for HIV prevention and treatment | Does national health coverage include medications for HIV treatment & pre-exposure prophylaxis (PrEP)? | National health coverage includes medications for HIV treatment & PrEP | National health coverage includes either ARVs or PrEP, but not both | National health coverage does not include ARVs or PrEP | United Nations, Political Declaration of the High-level Meeting on Universal Health Coverage, 2019; WHO Model List of Essential Medicines; United Nations, Political Declaration on HIV and AIDS, 2011 |
| D6 | 6. User fees | Are public primary healthcare and HIV services available without user fees at the point of service under national policy? | Most patients can access both primary healthcare and HIV services in public facilities without having to pay user fees | Most patients can access either primary healthcare or HIV services (but not both) in public facilities without having to pay user fees | Most patients are required to pay user fees to access both primary healthcare and HIV services in public facilities | United Nations, Political Declaration of the High-level Meeting on Universal Health Coverage, 2019; WHO, Primary Health Care on the Road to UHC, 2019; WHO, “The Practice of Charging User Fees at The Point of Service Delivery for HIV/AIDS Treatment and Care.” WHO Discussion Paper. WHO/HIV/2005.11. |
| D7 | 7. Access to medicines (TRIPS) | Does national law/policy take advantage of TRIPS flexibilities (compulsory licensing) for affordable medicines? | Country has incorporated TRIPS flexibilities into national law and has made use of those flexibilities | Country has either incorporated TRIPS flexibilities into national law or has made use of those flexibilities, but not both | Country has neither incorporated TRIPS flexibilities into national law nor made use of those flexibilities | United Nations, Political Declaration on HIV and AIDS, 2011; Doha Declaration on the TRIPS Agreement and Public Health, 2001 |
| D8 | 8. Unique identifiers w/ data protections | Are unique identifiers for continuity of care across multiple facilities included in national policy along with protections for patients' privacy? | The country utilizes unique IDs and has legally enforceable data privacy protections | The country utilizes unique IDs or has legally-enforceable data privacy protections, but not both | The country neither utilizes unique IDs nor has legally-enforceable data privacy protections | Global Commission on HIV and the Law, Risks, Rights & Health Supplement, 2018; UNAIDS, Fast-Track and Human Rights, 2017; UNAIDS, Developing and Using Individual Identifiers for the Provision of Health Services including HIV, 2009. |
| D9 | 9. Data sharing | Is it national policy to publicly share disaggregated HIV data on a regular basis? | The country shares disaggregated data at least quarterly | The country shares data on a quarterly basis but it is not disaggregated, or shares disaggregated data but not on a quarterly basis | The country does not share disaggregated data on a quarterly basis | UNAIDS Fast-Track Strategy 2016-2021 |

# Appendix III. Peer-reviewed literature

## Table S3. Search queries for Literature Review

| **Database** | **Search queries** |
| --- | --- |
| **PubMed** | ***Search query 1:*** *Country[tiab] AND HIV[tiab] AND (policy[tiab] OR strategy[tiab] OR program[tiab] OR guidelines[tiab] OR standards[tiab] OR law[tiab] OR legislation[tiab] OR plan[tiab])*  ***Search query 2:*** *Country[tiab] AND (gay men[tiab] OR men who have sex with men[tiab] OR sex worker[tiab] OR transgender[tiab] OR people who inject drugs[tiab] OR injection drug use[tiab] OR incarcerated people[tiab] OR prison population[tiab]) AND (policy[tiab] OR strategy[tiab] OR program[tiab] OR guidelines[tiab] OR standards[tiab] OR law[tiab] OR legislation[tiab] OR plan[tiab])* |
| **Web of Science** | ***Search query 1:*** *AB=(Country) AND AB=(hiv) AND (AB=(policy) OR AB=(strategy) OR AB=(program) OR AB=(guidelines) OR AB=(standards) OR AB=(law) OR AB=(legislation) OR AB=(plan))*  ***Search query 2:*** *AB=(Country) AND (AB=(“gay men”) OR AB=(“men who have sex with men”) OR AB=(“sex worker”) OR AB=(transgender) OR AB=(“people who inject drugs”) OR AB=(“injection drug use”) OR AB=(“incarcerated people”) OR AB=(“prison population”)) AND (AB=(policy) OR AB=(strategy) OR AB=(program) OR AB=(guidelines) OR AB=(standards) OR AB=(law) OR AB=(legislation) OR AB=(plan))* |
| **Google Scholar** | ***Search query 1:*** *allintitle: COUNTRY HIV policy OR strategy OR program OR guidelines OR standards OR law OR legislation OR plan*  ***Search query 2:*** *allintitle: COUNTRY policy OR strategy OR program OR guidelines OR standards OR law OR legislation OR plan "POPULATION KEYWORD"* |

# Appendix IV. Complete dataset of policies

## Table S4. Complete dataset of policies found

| **Country** | **Title of policy and link** | **Policy ID** | **Policy and sections relevant to the policy area** | **Area of intervention** | **Targeted HIV key and priority populations** | **Direct or indirect impact on key/priority populations** | **HIV policy target adoption** | **Facilitates or hinders HIV interventions** |
| --- | --- | --- | --- | --- | --- | --- | --- | --- |
| Belgium | Act reforming transgender regulations as regards the indication of an adjustment of the registration of gender in civil status records and its consequences (*Wet tot hervorming van regelingen inzake transgenders wat de vermelding van een aanpassing van de registratie van het geslacht in de akten van de burgerlijke stand en de gevolgen hiervan betreft* / *Loi réformant des régimes relatifs aux personnes transgenres en ce qui concerne la mention d'une modification de l'enregistrement du sexe dans les actes de l'état civil et ses effets*) http://www.ejustice.just.fgov.be/eli/wet/2017/06/25/2017012964/justel | C9.2 | Act reforming transgender regulations | Prevention, Testing, Treatment, Care | Transgender people | Indirect | Adopted | Facilitates |
| Belgium | Act to punish certain acts inspired by racism or xenophobia (*Wet tot bestraffing van bepaalde door racisme of xenophobie ingegeven daden / Loi tendant à réprimer certains actes inspirés par le racisme ou la xénophobie*) http://www.ejustice.just.fgov.be/eli/wet/1981/07/30/1981001359/justel | C8-E | Anti-Racism Law. Articles 3, 4 and 5 | Prevention, Testing, Treatment, Care | (Undocumented) migrants from high HIV prevalence countries, All fulfill that may be faced with discrimination based on the criteria listed in the law | Indirect | Adopted | Facilitates |
| Belgium | Agreement between the Health Insurance Committee and the ... on behalf of the HIV Reference Center ... (Convention entre le Comité de l'assurance soins de santé et le ... au nom du Centre de Référence VIH ...) https://www.inami.fgov.be/SiteCollectionDocuments/convention_sida.pdf | D5.2 | Model agreement. Sections 10 to 20 | Prevention, Testing, Treatment, Care | Pregnant people with HIV or pregnant people with AIDS, infants who have been exposed in utero to antiretroviral treatments that are still on stage of diagnostic uncertainty as well as their parents, PLHIV | Direct | Adopted | Facilitates |
| Belgium | Anti-Discrimination Law (*Wet ter bestrijding van bepaalde vormen van discriminatie* / *Loi tendant à lutter contre certaines formes de discrimination* / *Gesetz zur Bekämpfung bestimmter Formen von Diskriminierung*) http://www.ejustice.just.fgov.be/eli/wet/2007/05/10/2007002099/justel | C8 | Anti-Discrimination Law. Article 4 | Prevention, Testing, Treatment, Care | All individuals that fulfill any of the criteria that could be grounds for discrimination and that are covered by this law. | Indirect | Adopted | Facilitates |
| Belgium |  | C8-A | Anti-Discrimination Law. Article 4 | Prevention, Testing, Treatment, Care | PLHIV | Indirect | Adopted | Facilitates |
| Belgium |  | C8-B | Anti-Discrimination Law. Article 4 | Prevention, Testing, Treatment, Care | Gay men and other MSM | Indirect | Adopted | Facilitates |
| Belgium | Basic Law on the prison system and the legal status of prisoners (*Basiswet betreffende het gevangeniswezen en de rechtspositie van de gedetineerden / Loi de principes concernant l'administration pénitentiaire ainsi que le statut juridique des détenus*) http://www.ejustice.just.fgov.be/eli/loi/2005/01/12/2005009033/justel | A8 | Law on the prison system. Article 88 | Prevention, Testing, Treatment, Care | Incarcerated people | Indirect | Adopted | Facilitates |
| Belgium | Belgium National PrEP Guidelines (*Lignes directrices nationales pour la PrEP en Belgique*) https://www.prepwatch.org/wp-content/uploads/2022/03/Belgium-National-PrEP-Guidelines-2019.pdf | B6.2 | National PrEP guidelines | Prevention | Gay men and other MSM, Sex workers, Transgender people, People who inject drugs, PLHIV sexual partners | Direct | Adopted | Facilitates |
| Belgium | Civil Code (*Burgerlijk Wetboek* / *Code Civil* / *Zivilgesetzbuch)* http://www.ejustice.just.fgov.be/eli/wet/1804/03/21/1804032150/justel | C16 | Civil Code. Article 143 | Prevention, Testing, Treatment, Care | Gay men and other MSM, Transgender people, Lesbians | Indirect | Adopted | Facilitates |
| Belgium | Code of Economic Law, of February 28, 2013 (Wetboek van Economisch Recht / Code de droit économique) http://www.ejustice.just.fgov.be/eli/wet/2013/02/28/2013A11134/justel | D7 | Code of Economic Law. Articles XI.37 to XI.46 | Prevention, Treatment | General population | Indirect | Adopted | Facilitates |
| Belgium | Coordination of the Royal Decree of 19 March 2004 regulating treatment with substitutes with the Royal Decree of 6 October 2006 (*Coördinatie van het koninklijk besluit van 19 maart 2004 tot reglementering van de behandeling met vervangingsmiddelen met het koninklijk besluit van 6 oktober 2006 / Coordination de l’arrêté royal du 19 mars 2004 réglementant le traitement de substitution avec l’arrêté royal du 6 octobre 2006) https://www.klav.be/klavinfo/files/Dossiers/Methadonsubstitutie/Wettelijke%20basis.pdf* | C6 | Royal Decree. Articles 2, 3, 4, 6 and 9 | Prevention | People who inject drugs | Direct | Adopted | Facilitates |
| Belgium | Criminal Code (*Strafwetboek / Code pénal) http://www.ejustice.just.fgov.be/eli/wet/1867/06/08/1867060850/justel* | C1 | Criminal code conferres no penal sanctions | Prevention, Testing, Treatment, Care | Gay men and other MSM | Indirect | Adopted | Facilitates |
| Belgium |  | C7 | Criminal Code. Article 402 | Prevention, Testing, Treatment, Care | PLHIV | Indirect | Partially adopted | Hinders |
| Belgium | Federal Drug Policy Note (*Beleidsnota van de Federale Regering in verband met de drugproblematiek / Note politique du Gouvernement fédéral relative à la problématique de la drogue*) http://www.emcdda.europa.eu/system/files/media/country_overview/documents/2557/Belgium%20Policy%20Note%202001-%20French.pdf | B7.1 | Federal Drug Policy Note. Sections 1.2 and 4.4 | Prevention | People who inject drugs, Users of both illicit and licit substances | Direct | Adopted | Facilitates |
| Belgium | Federal Institute for the Protection and Promotion of Human Rights (*Federaal Instituut voor de Rechten van de Mens, FIRM*) https://federaalinstituutmensenrechten.be/nl | C10.2 | Federal Institute for the Protection and Promotion of Human Rights | Prevention, Testing, Treatment, Care | General population | Indirect | Partially adopted | Facilitates |
| Belgium | Guidelines for HIV viral load measurement in HIV patients (*Richtlijnen voor de bepaling van virale lading bij HIV patiënten / Directives pour la mesure de la charge virale du VIH chez les patients séropositifs*) https://www.sciensano.be/fr/laboratoires-de-reference-sida-lrs/mesure-de-la-charge-virale-du-vih-chez-les-patients-seropositifs | A5 | Guidelines for HIV viral load measurement | Testing, Treatment | PLHIV | Direct | Adopted | Facilitates |
| Belgium | HIV Plan 2014-2019 (*HIV-Plan 2014-2019 /* *Plan VIH 2014-2019*) https://hiv-plan.be/wp-content/uploads/2022/01/HIV-plan_ENG.pdf | A1.1 | HIV Plan. Action 36 | Prevention, Treatment | PLHIV | Direct | Adopted | Facilitates |
| Belgium |  | A2 | HIV Plan. Action 36 | Treatment, Prevention, Treatment | PLHIV | Direct | Adopted | Facilitates |
| Belgium | HIV surveillance by Sciensano, the Belgian Health Institute https://www.sciensano.be/sites/default/files/rapport_vih_2022_fr.pdf | D9 | HIV surveillance | Prevention, Testing, Treatment, Care | All PLHIV and PrEP and PEP users | Direct | Partially adopted | Facilitates |
| Belgium | Joint circular letter from the Minister of Justice and the Board of Prosecutors General on the establishment, registration and prosecution policy regarding the possession of and the retail trade in illicit narcotics (Dutch: *Gemeenschappelijke omzendbrief van de minister van Justitie en het College van Procureurs-generaal betreffende de vaststelling, de registratie en het vervolgingsbeleid inzake het bezit van en de detailhandel in illegale verdovende middelen)* ***https://www.om-mp.be/sites/default/files/u1/col15_2015_herzien2018.zip*** French: *Circulaire commune du ministre de la Justice et du Collège des procureurs généraux relative à la constatation, l’enregistrement et la politique des poursuites en matière de détention et de vente au détail de drogues illicites*) | B7.3 | Joint circular letter. Section 5.4 | Prevention | People who inject drugs, Users of ilicit substances | Indirect | Adopted | Facilitates |
| Belgium | Joint Declaration of the Interministerial Conference on Drugs (*Gemeenschappelijke Verklaring van de Interministeriële Conferentie Drugs / Déclaration Conjointe de la Conférence Interministérielle Drogues*) https://www.jeminforme.be/images/drogues/Declaration-PolitiqueGlobaleDrogues-25012010.pdf | B7.2 | Joint Declaration. Section 2.2 | Prevention | People who inject drugs, Users of both illicit and licit substances | Direct | Adopted | Facilitates |
| Belgium |  | C4.2 | Joint Declaration. Sections 2.1 and 2.2 | Prevention | People who inject drugs, All drug users | Direct | Adopted | Facilitates |
| Belgium | Law aimed at repressing non-compliance with the temporary ban on residence in the event of domestic violence and modifying articles 594 and 627 of the Judicial Code (*Wet tot bestraffing van de overtreding van het tijdelijk huisverbod en tot wijziging van artikelen 594 en 627 van het Gerechtelijk Wetboek* / *Loi tendant à réprimer le non-respect de l'interdiction temporaire de résidence en cas de violence domestique et modifiant les articles 594 et 627 du Code judicaire*)  https://www.ejustice.just.fgov.be/cgi/article_body.pl?language=fr&caller=summary&pub_date=12-10-01&numac=2012009264 | C13.3 | Judicial Code. Articles 594 and 627 modified | Prevention, Testing, Treatment, Care | General population | Indirect | Adopted | Facilitates |
| Belgium | Law allocating the family home to the spouse or legal cohabitant who is the victim of physical violence committed by his partner and supplementing Article 410 of the Criminal Code (*Wet tot toewijzing van de gezinswoning aan de echtgenoot of aan de wettelijk samenwonende die het slachtoffer is van fysieke gewelddaden vanwege zijn partner en tot aanvulling van artikel 410 van het Strafwetboek* / *Loi visant a` l’attribution du logement familial au conjoint ou au cohabitant le´gal victime d’actes de violence physique de son partenaire, et comple´tant l’article 410 du Code pe´nal*) https://www.ejustice.just.fgov.be/mopdf/2003/02/12_1.pdf#Page6 | C13.1 | Criminal Code. Article 410 supplemented | Prevention, Testing, Treatment, Care | General population | Indirect | Adopted | Facilitates |
| Belgium | Law amending the Criminal Code with regard to sexual criminal law (*Wet houdende wijzigingen aan het Strafwetboek met betrekking tot het seksueel strafrecht / Loi modifiant le Code pénal en ce qui concerne le droit pénal sexuel*) https://www.ejustice.just.fgov.be/cgi_loi/change_lg.pl?language=nl&la=N&table_name=wet&cn=2022032101 | C2 | Criminal Code. Articles 379, 380, and 380bis repealed | Prevention, Testing, Treatment, Care | Sex workers | Indirect | Adopted | Facilitates |
| Belgium | Law amending the law of February 24, 1921 on the trade in poisons, sleeping pills and narcotics, disinfectants and antiseptics and the Royal Decree no. 78 of November 10, 1967 on the practice of medicine, nursing , the paramedical professions and the medical commissions (*Wet tot wijziging van de wet van 24 februari 1921 betreffende het verhandelen van de giftstoffen, slaapmiddelen en verdovende middelen, ontsmettingsstoffen en antiseptica en het koninklijk besluit nr. 78 van 10 november 1967 betreffende de uitoefening van de geneeskunst, de verpleegkunde, de paramedische beroepen en de geneeskundige commissies* / *Loi modifiant la loi du 24 février 1921 concernant le trafic des substances vénéneuses, soporifiques, stupéfiantes, désinfectantes ou antiseptiques et l’arrêté royal n° 78 du 10 novembre 1967 relatif à l’exercice de l’art de guérir, de l’art infirmier, des professions paramédicales et aux commissions médicales*) https://www.ejustice.just.fgov.be/mopdf/1998/12/23_1.pdf#Page5 | B7.4 | Drugs Act. Article 3 | Prevention | People who inject drugs | Indirect | Adopted | Facilitates |
| Belgium | Law on Combating Discrimination between Women and Men (*Wet ter bestrijding van discriminatie tussen vrouwen en mannen / Loi tendant à lutter contre la discriminination entre les femmes et les hommes*) https://www.jeminforme.be/wp-content/uploads/2023/04/LB-DiscriminationEntreFemmesHommes-10052007-Maj15022023.pdf | C8-D | Gender Act. Article 4 | Prevention, Testing, Treatment, Care | Transgender people | Indirect | Adopted | Facilitates |
| Belgium | Law on medical examinations in the context of labor relations (*Wet betreffende de medische onderzoeken die binnen het kader van de arbeidsverhoudingen worden uitgevoerd / Loi relative aux examens médicaux dans le cadre des relations de travail) https://www.jeminforme.be/images/sida/LB-ExamensMedicauxDansLeCadreDesRelationsDeTravail-SPFEmploi-23012003Maj21042016.pdf* | B4.3 | Law on medical examinations. Article 3 (1) | Testing | All employees and job candidates in the private and public sector | Direct | Adopted | Facilitates |
| Belgium | Law on Public Centers for Social Welfare (*Loi organique des centres publics d’action sociale / Organieke wet betreffende de openbare centra voor maatschappelijk welzijn) https://wallex.wallonie.be/eli/loi-decret/1976/07/08/197670810/2023/02/01* | A7.1 | Law on Public Centers for Social Welfare. Article 57 (2) | Prevention, Testing, Treatment, Care | (Undocumented) migrants from high HIV prevalence countries, All persons who reside in Belgium illegally | Indirect | Adopted | Facilitates |
| Belgium | Law on the temporary prohibition of residence in the event of domestic violence (*Wet betreffende het tijdelijk huisverbod in geval van huiselijk geweld* / *Loi relative à l'interdiction temporaire de résidence en cas de violence domestique*) https://www.ejustice.just.fgov.be/cgi/article_body.pl?language=fr&caller=summary&pub_date=12-10-01&numac=2012009239 | C13.2 | Law on the temporary prohibition of residence in the event of domestic violence | Prevention, Testing, Treatment, Care | All people at risk of experiencing domestic violence | Indirect | Adopted | Facilitates |
| Belgium | Law on the trade of poisonous, soporific, narcotic, psychotropic, disinfectant or antiseptic substances and substances that can be used in the illicit manufacture of narcotics and psychotropic substances (*Wet betreffende het verhandelen van giftstoffen, slaapmiddelen en verdovende middelen, psychotrope stoffen, ontsmettingsstoffen en antiseptica en van de stoffen die kunnen gebruikt worden voor de illegale vervaardiging van verdovende middelen en psychotrope stoffen* / *Loi concernant le trafic des substances vénéneuses, soporifiques, stupéfiantes, psychotropes, désinfectantes ou antiseptiques et des substances pouvant servir à la fabrication illicite de substances stupéfiantes et psychotropes*) http://www.ejustice.just.fgov.be/eli/wet/1921/02/24/1921022450/justel | C3 | Drug Law. Article 2(2) | Prevention, Testing, Treatment, Care | People who inject drugs | Indirect | Not adopted | Facilitates / Hinders |
| Belgium | Law on Transsexuality (*Wet betreffende de transseksualiteit* / *Loi relative à la transsexualité*) http://www.ejustice.just.fgov.be/eli/wet/2007/05/10/2007009570/justel | C9.1 | Law on Transsexuality. Article 2 | Prevention, Testing, Treatment, Care | Transgender people | Indirect | Adopted | Facilitates |
| Belgium | Law regarding compulsory insurance for medical care and benefits coordinated on 14 July 1994 (*Wet betreffende de verplichte verzekering voor geneeskundige verzorging en uitkeringen gecoördineerd op 14 juli 1994 / Loi relative à l'assurance obligatoire soins de santé et indemnités coordonnée le 14 juillet 1994*) http://www.ejustice.just.fgov.be/eli/wet/1994/07/14/1994071451/justel | D5.1 | Health Insurance Act. Articles 14 to 31 bis, 34, 35 | Prevention, Testing, Treatment, Care | Beneficieries of the medical benefits | Indirect | Adopted | Facilitates |
| Belgium |  | D6 | Health Insurance Act. Articles 37 and 37 bis | Prevention, Testing, Treatment, Care | All patients, including PLHIV or people at high risk of HIV infection | Indirect | Not adopted | Hinders |
| Belgium | Ministerial Decree amending the list appended to the Royal Decree of 21 December 2001 establishing the procedures, terms and conditions regarding the contribution of the compulsory insurance for medical care and benefits towards the costs of pharmaceutical specialties, of June 2017 (*Ministerieel besluit tot wijziging van de lijst gevoegd bij het koninklijk besluit van 21 december 2001 tot vaststelling van de procedures, termijnen en voorwaarden inzake de tegemoetkoming van de verplichte verzekering voor geneeskundige verzorging en uitkeringen in de kosten van farmaceutische specialiteiten /* *Arrêté ministériel modifiant la liste jointe à l'arrêté royal du 21 décembre 2001 fixant les procédures, délais et conditions en matière d'intervention de l'assurance obligatoire soins de santé et indemnités dans le coût des spécialités pharmaceutiques*) https://www.ejustice.just.fgov.be/mopdf/2017/06/20_2.pdf#Page56 | B6.1 | Ministerial Decree. Article 2 | Prevention |  | Direct | Adopted | Facilitates |
| Belgium | Patient Rights Act (*Wet betreffende de rechten van de patiënt* / *Loi relative aux droits du patient*) http://www.ejustice.just.fgov.be/eli/wet/2002/08/22/2002022737/justel | B5 | Patient Rights Act. Article 12 | Testing, Treatment | All patients (Art. 12 concerns minors specifically) | Indirect | Adopted | Facilitates |
| Belgium |  | D8 | Patient Rights Act. Article 10 | Prevention, Testing, Treatment, Care | All patients | Indirect | Partially adopted | Facilitates |
| Belgium | Proposal of the Monitoring Committee for a renewed national HIV plan for the period 2020-2026 paying particular attention to STIs (Voorstel van het HIV Monitoring Comité voor een voortgezet nationaal HIV Plan 2020-2026 met bijzondere aandacht voor soa’s / *Proposition du Comité de Monitoring pour un plan national HIV reconduit pour la période 2020-2026 accordant une attention particulière aux IST*) https://www.arl.ugent.be/file/402/download?token=yO95f-f6 | A1.2 | National HIV plan. Priority Area 4 | Prevention, Treatment | PLHIV | Direct | Adopted | Facilitates |
| Belgium |  | A12 | National HIV plan. Priority Areas 2 and 4 | Prevention, Treatment, Care | PLHIV | Direct | Adopted | Facilitates |
| Belgium |  | B2 | National HIV plan. Priority Area 2 | Testing | Gay men and other MSM, Sex workers, Transgender people, People who inject drugs, (Undocumented) migrants from high HIV prevalence countries, Individuals who do not get tested in a health center or in a community structure | Direct | Adopted | Facilitates |
| Belgium |  | B4.1 | National HIV plan. Pillar 2 | Testing | General population, including key populations | Direct | Adopted | Facilitates |
| Belgium |  | B14 | National HIV plan. Priority Area 2 | Prevention | Gay men and other MSM, Sex workers, (Undocumented) migrants from high HIV prevalence countries, | Direct | Adopted | Facilitates |
| Belgium |  | D1 | National HIV plan | Prevention, Testing, Treatment, Care | Gay men and other MSM, Sex workers, Transgender people, People who inject drugs, Incarcerated people, (Undocumented) migrants from high HIV prevalence countries | Direct | Adopted | Facilitates |
| Belgium |  | D2 | National HIV plan. Section IV | Prevention, Testing, Treatment, Care | Gay men and other MSM, Sex workers, Transgender people, People who inject drugs, Incarcerated people, (Undocumented) migrants from high HIV prevalence countries | Direct | Adopted | Facilitates |
| Belgium | Policy note from the Federal Government relating to the drug problem (*Beleidsnota van de Federale Regering in verband met de drugproblematiek /* *Note politique du Gouvernement fédéral relative à la problématique de la drogue*) http://www.emcdda.europa.eu/system/files/media/country_overview/documents/2557/Belgium%20Policy%20Note%202001-%20French.pdf | C4.1 | Federal Drug Policy Note. Sections 1.2, 4.4, 4.5, and 4.5.3 | Prevention | People who inject drugs, All drug users | Direct | Adopted | Facilitates |
| Belgium | Royal Decree establishing the conditions under which the Insurance Committee can enter into agreements regulating the contribution of the compulsory insurance for medical care to the benefits for specific forms of the fight against AIDS (*Arrêté royal fixant les conditions dans lesquelles le Comité de l'assurance peut conclure des conventions visant à régler l'intervention de l'assurance soins de santé obligatoire dans les prestations pour des formes spécifiques de lutte contre le SIDA* */ kundige verzorging in de prestaties voor specifieke vormen van aidsbestrijding*) https://www.ejustice.just.fgov.be/cgi/article_body.pl?language=fr&caller=summary&pub_date=17-03-23&numac=2017011227 | B1 | Royal Decree. Articles 4 and 6(1) | Testing | Gay men and other MSM, Sex workers, People who inject drugs, (Undocumented) migrants from high HIV prevalence countries, Partners of sex workers, Clients of prostitution and their partners, Persons who have sexual relations outside the context of a stable relationship, Persons who have several partners or who have sexual relations with a partner who has multiple partners | Direct | Adopted | Facilitates |
| Belgium |  | B10 | Royal Decree. Article 4 | Prevention, Testing | Gay men and other MSM | Direct | Adopted | Facilitates |
| Belgium | Royal decree implementing article 124, 1° of the law of 10 May 2015 on the exercise of health care professions, with a view to regulating diagnostic orientation tests for HIV human immunodeficiency virus infection (*Koninklijk besluit houdende de toepassing van artikel 124, 1° van de wet van 10 mei 2015 betreffende de uitoefening van de gezondheidsberoepen teneinde een kader te scheppen voor de diagnostische oriëntatietests voor het humaan immuundeficiëntievirus HIV / Arrêté royal portant application de l'article 124, 1° de la loi du 10 mai 2015 relative à l'exercice des professions de soins de santé, en vue d'encadrer les tests d'orientation diagnostique de l'infection du virus de l'immunodéficience humaine VIH*) http://www.ejustice.just.fgov.be/eli/arrete/2018/07/19/2018031817/moniteur | B4.2 | Royal Decree. Article 2 | Testing | Gay men and other MSM, Sex workers, Transgender people, People who inject drugs, Incarcerated people, (Undocumented) migrants from high HIV prevalence countries | Direct | Adopted | Facilitates |
| Belgium | Royal Decree implementing Article 4, § 2, 6° of Royal Decree No. 78 of 10 November 1967 on the practice of medicine, nursing, paramedical professions and medical commissions (*Koninklijk besluit tot uitvoering van artikel 4, § 2, 6° van het koninklijk besluit nr. 78 van 10 november 1967 betreffende de uitoefening van de geneeskunst, de verpleegkunde, de paramedische beroepen en de geneeskundige commissies / Arrêté royal portant exécution de l’article 4, § 2, 6° de l’arrêté royal n° 78 du 10 novembre 1967 relatif à l’exercice de l’art de guérir, de l’art infirmier, des professions paramédicales et aux commissions médicales*) https://www.ejustice.just.fgov.be/mopdf/2000/07/07_1.pdf#Page10 | C5 | Royal Decree. Article 1 | Prevention | People who inject drugs | Direct | Adopted | Facilitates |
| Belgium | Royal Decree on urgent medical aid provided by public social welfare centers to foreigners staying illegally in the Kingdom (*Arrêté royal relatif à l’aide médicale urgente octroyée par les centres publics d’aide sociale aux étrangers qui séjournent illégalement dans le Royaume / Koninklijk besluit betreffende de dringende medische hulp die door de openbare centra voor maatschappelijk welzijn wordt verstrekt aan de vreemdelingen die onwettig in het Rijk verblijven*) http://www.mi-is.be/sites/default/files/documents/KB%201996-12-12_4.pdf | A7.2 | Royal Decree. Article 1 | Prevention, Testing, Treatment, Care | (Undocumented) migrants from high HIV prevalence countries, All persons who reside in Belgium illegally | Indirect | Adopted | Facilitates |
| Belgium | The Constitution (*De Grondwet / La Constitution / Die Verfassung*) https://www.senate.be/doc/const_fr.html | C11 | The Constitution. Articles 7bis and 23 | Prevention, Testing, Treatment, Care | General population | Indirect | Adopted | Facilitates |
| Belgium |  | C14 | The Constitution. Articles 7bis and 27 | Prevention, Testing, Treatment, Care | General population | Indirect | Partially adopted | Facilitates |
| Belgium | Unia, Interfederal Centre for Equal Opportunities https://www.unia.be/fr | C10.1 | Interfederal Centre for Equal Opportunities | Prevention, Testing, Treatment, Care | General population | Indirect | Partially adopted | Facilitates |
| Belgium (Flanders) | Decree containing a framework for the Flemish equal opportunities and equal treatment policy (*Decreet houdende een kader voor het Vlaamse gelijkekansen- en gelijkebehandelingsbeleid*) https://codex.vlaanderen.be/portals/codex/documenten/1017082.html | C8 | Equal opportunities and equal treatment policy. Article 6(2) | Prevention, Testing, Treatment, Care | All people on Flemish soil | Indirect | Adopted | Facilitates |
| Belgium (Flanders) |  | C8-A | Equal opportunities and equal treatment policy. Article 6(2) | Prevention, Testing, Treatment, Care | PLHIV | Indirect | Adopted | Facilitates |
| Belgium (Flanders) |  | C8-B | Equal opportunities and equal treatment policy. Article 6(2) | Prevention, Testing, Treatment, Care | Gay men and other MSM, Lesbian women, asexual people, bisexual people | Indirect | Adopted | Facilitates |
| Belgium (Flanders) |  | C8-D | Equal opportunities and equal treatment policy. Article 6(2) | Prevention, Testing, Treatment, Care | Transgender people | Indirect | Adopted | Facilitates |
| Belgium (Flanders) |  | C8-E | Equal opportunities and equal treatment policy. Article 6(2) | Prevention, Testing, Treatment, Care | (Undocumented) migrants from high HIV prevalence countries, Anyone experiencing discrimination based on race, skin colour, descent, or ethnic or national origin, religion or philosophy of life | Indirect | Adopted | Facilitates |
| Belgium (Flanders) | Educational goals *(Onderwijsdoelen*) https://onderwijsdoelen.be/ | B8 | Educational goals | Prevention | Students in secondary edcuation | Indirect | Adopted | Facilitates |
| Belgium (Flanders) | Flemish Human Rights Institute https://www.vlaamsmensenrechteninstituut.be/ | C10 | Flemish Human Rights Institute | Prevention, Testing, Treatment, Care | All people living in Flanders and that might be victims of discrmimination | Indirect | Adopted | Facilitates |
| Belgium (Flanders) | Revised strategic plan 'The Flemish live healthier in 2025' (*Herziening van het strategisch plan ‘De Vlaming leeft gezonder in 2025’*) https://www.zorg-en-gezondheid.be/sites/default/files/2022-12/GezLev_Herziening_strategisch_plan_FINAL.pdf | B13 | The Flemish live healthier in 2025 |  | Flemish population | N/A | Partially adopted | N/A |
| Belgium (Flanders) | Syringe Exchange Program implemented by Free Clinic https://zorg-en-gezondheid.be/per-domein/preventie/organisaties-met-terreinwerking/free-clinic | C5 | Syringe Exchange Program | Prevention | People who inject drugs | Direct | Adopted | Facilitates |
| Belgium (Flanders) | Violett https://www.violett.be/en/about-violett/who-are-we/ | B9 | Violett | Prevention | Sex workers, Customers of Sex workers | 1. Indirect | Adopted | Facilitates |
| Czech Republic | Act No. 115/2006 Coll. The Act on Registered Partnerships and on Amendments to Certain Related Acts *(Zákon č. 115/2006 Sb. Zákon o registrovaném partnerství a o změně některých souvisejících zákonů)* https://www.zakonyprolidi.cz/cs/2006-115 | C16 | Act on Registered Partnerships | Prevention, Testing, Treatment, Care | Gay men and other MSM, Transgender people, Lesbians | Indirect | Not adopted | Hinders |
| Czech Republic | Act No. 198/2009 Coll. Act on Equal Treatment and Legal Means of Protection Against Discrimination and on Amendments to Certain Acts (Anti-Discrimination Act) *(Zákon č. 198/2009 Sb. Zákon o rovném zacházení a o právních prostředcích ochrany před diskriminací a o změně některých zákonů)* https://www.zakonyprolidi.cz/cs/2009-198 | C8.1 | Anti-Discrimination Act. Section 2 | Prevention, Testing, Treatment, Care | Gay men and other MSM, Transgender people, General population | Indirect | Adopted | Facilitates |
| Czech Republic |  | C8-B | Anti-Discrimination Act. Section 2 | Prevention, Testing, Treatment, Care | Gay men and other MSM | Indirect | Adopted | Facilitates |
| Czech Republic |  | C8-D | Anti-Discrimination Act. Section 2 | Prevention, Testing, Treatment, Care | Transgender people | Indirect | Adopted | Facilitates |
| Czech Republic |  | C8-E | Anti-Discrimination Act. Section 2 | Prevention, Testing, Treatment, Care | Foreigners from countries with a higher risk of HIV (Migrants), | Indirect | Adopted | Facilitates |
| Czech Republic | Act No. 373/2011 Coll. Act on Specific Health Services *(Zákon č. 373/2011 Sb. Zákon o specifických zdravotních službách)* https://www.zakonyprolidi.cz/cs/2011-373 | C9 | Act on Specific Health Services. Part 4, sections 21 and 22 | Prevention, Testing, Treatment, Care | Transgender people | Indirect | Adopted | Facilitates |
| Czech Republic | Act No. 48/1997 on Public Health Insurance [as amended] *(Zákon č. 48/1997 Sb. o veřejném zdravotním pojištění a o změně a doplnění některých souvisejících zákonů)* https://www.zakonyprolidi.cz/cs/1997-48 | A7 | Act on Public Health Insurance. Section 2 | Prevention, Treatment | Foreigners from countries with a higher risk of HIV (Migrants), | Indirect | Not adopted | Hinders |
| Czech Republic |  | D5 | Act on Public Health Insurance. Part 5 | Testing, Treatment | General population, PLHIV | Direct | Partially adopted | Facilitates |
| Czech Republic | Act No. 527 / 1990 Coll. on Inventions and Rationalisation Proposals *(527/1990 Sb. ZÁKON ze dne 27. listopadu 1990 o vynálezech a zlepšovacích návrzích)* https://upv.gov.cz/files/uploads/PDF_Dokumenty/legislation/national/527_1990-072014_en.pdf | D7 | Act on Inventions and Rationalisation Proposals |  | General public | Indirect | Partially adopted | Facilitates |
| Czech Republic | Act No. 561/2004 Coll. Act on Pre-school, Primary, Secondary, Higher Vocational and Other Education (Education Act) *(Zákon č. 561/2004 Sb. Zákon o předškolním, základním, středním, vyšším odborném a jiném vzdělávání)* https://www.zakonyprolidi.cz/cs/2004-561#p36 | C12.2 | Act on Pre-school, Primary, Secondary, Higher Vocational and Other Education. Parts 1 and 3 | Prevention | General population | Indirect | Adopted | Facilitates |
| Czech Republic | Act No. 89/2012 Coll. Law Civil Code *(Zákon č. 89/2012 Sb. Zákon občanský zákoník)* https://www.zakonyprolidi.cz/cs/2012-89 | C14 | Law Civil Code. Chapter 1 | Prevention, Testing, Treatment, Care | PLHIV | Indirect | Partially adopted | Facilitates |
| Czech Republic | Framework Education Programme for Secondary General Education *(Rámcový vzdělávací program pro gymnázia)* https://www.edu.cz/wp-content/uploads/2021/09/001_RVP_GYM_uplne_zneni.pdf | B8.3 | Framework Education Programme. Chapter 5 | Prevention | General population | Indirect | Adopted | Facilitates |
| Czech Republic | Framework Educational Programme for Basic Education *(Rámcový vzdělávací program pro základní vzdělávání) https://www.msmt.cz/file/56005_1_1/* | B8.2 | Framework Education Programme. Chapter 5 | Prevention | General population | Indirect | Adopted | Facilitates |
| Czech Republic | HIV pre-exposure prophylaxis (PrEP): Appendix No. 2 of the Recommended Procedure for the Care of HIV-Infected Adults and Post-Exposure Prophylaxis HIV infection *(Preexpoziční profylaxe HIV (PrEP): Příloha č. 2 Doporučeného postupu péče o dospělé infikované HIV a postexpoziční profylaxe infekce HIV)* https://infektologie.cz/Legislativa/DP-HIV-2-PrEP-0921p.pdf | B6 | PrEP Guidelines | Prevention | Gay men and other MSM, Sex workers, Transgender people, People who inject drugs, Incarcerated people, foreigners from countries with a higher risk of HIV (Migrants), General population | Direct | Adopted | Facilitates |
| Czech Republic | Methods to solve the issue of HIV/AIDS infection in the Czech Republic *(Metodický Návod Křešení problematiky infekce HIV/AIDS V České Republice)* https://www.mzcr.cz/wp-content/uploads/2023/01/Vestnik-MZ_01-2023.pdf | A4 | HIV/AIDS guidelines. Article 3 | Prevention, Testing, Treatment, Care | PLHIV | Direct | Not adopted | Hinders |
| Czech Republic |  | B1.2 | HIV/AIDS guidelines. Article 2 | Testing, Prevention | General population | Direct | Adopted | Facilitates |
| Czech Republic |  | B3 | HIV/AIDS guidelines. Articles 2 and 4 | Prevention, Testing | PLHIV and their sexual partners | Direct | Adopted | Facilitates |
| Czech Republic |  | B4 | HIV/AIDS guidelines. Article 2 | Testing | General population | Direct | Adopted | Facilitates |
| Czech Republic |  | D2.3 | HIV/AIDS guidelines. Articles 6 and 11 | Prevention, Testing, Treatment, Care | PLHIV | Direct | Adopted | Facilitates |
| Czech Republic | National program for solving the problem of HIV/AIDS in the Czech Republic for the period 2023–2027 *(Národní program řešení problematiky HIV/AIDS v ČR na období 2023–2027)* https://www.mzcr.cz/wp-content/uploads/2023/05/NP_HIV_AIDS-2023-2027.pdf | A8 | National HIV/AIDS program. Section 2.5 | Prevention, Treatment | Incarcerated people | Indirect | Adopted | Facilitates |
| Czech Republic |  | A12 | National HIV/AIDS program. Section 3.4 | Care | PLHIV | Direct | Adopted | Facilitates |
| Czech Republic |  | B1.1 | National HIV/AIDS program. Section 2.3 | Prevention, Testing | Gay men and other MSM, People who inject drugs, Sex workers, Foreigners from countries with a higher risk of HIV (Migrants), General population | Direct | Adopted | Facilitates |
| Czech Republic |  | B2 | National HIV/AIDS program. Section 2.3 | Testing | General population | Direct | Adopted | Facilitates |
| Czech Republic |  | B7.1 | National HIV/AIDS program. Section 2.5 | Prevention | People who inject drugs | Direct | Adopted | Facilitates |
| Czech Republic |  | B8.1 | National HIV/AIDS program. Section 2.5 | Prevention | General population | Indirect | Adopted | Facilitates |
| Czech Republic |  | B9 | National HIV/AIDS program. Section 2.5 | Prevention | Sex workers | Direct | Adopted | Facilitates |
| Czech Republic |  | B10 | National HIV/AIDS program. Section 2.5 | Prevention | Gay men and other MSM | Direct | Adopted | Facilitates |
| Czech Republic |  | B11 | National HIV/AIDS program. Section 2.5 | Prevention | Incarcerated people | Direct | Partially adopted | Facilitates |
| Czech Republic |  | B14 | National HIV/AIDS program. Section 3.4 | Prevention | Gay men and other MSM | Direct | Adopted | Facilitates |
| Czech Republic |  | C5 | National HIV/AIDS program. Section 2.5 | Prevention | People who inject drugs, Incarcerated people | Direct | Adopted | Facilitates |
| Czech Republic |  | C6.1 | National HIV/AIDS program. Section 2.5 | Prevention | People who inject drugs, Incarcerated people | Direct | Adopted | Facilitates |
| Czech Republic |  | D1 | National HIV/AIDS Program | Prevention, Testing, Treatment, Care | Gay men and other MSM, Sex workers, Transgender people, People who inject drugs, Incarcerated people, foreigners from countries with a higher risk of HIV (Migrants), | Direct | Adopted | Facilitates |
| Czech Republic |  | D2.1 | National HIV/AIDS Program. Chapter 5 | Prevention, Testing, Treatment, Care | PLHIV | Direct | Adopted | Facilitates |
| Czech Republic |  | D4 | National HIV/AIDS Program. Section 2.7 | Prevention, Testing, Treatment, Care | General population, PLHIV | Direct | Adopted | Facilitates |
| Czech Republic |  | D9 | National HIV/AIDS Program. Section 5.3 | Prevention, Testing, Treatment, Care | PLHIV | Direct | Adopted | Facilitates |
| Czech Republic | National Strategy to Prevent and Reduce the Harm Associated with Addictive Behaviour 2019-2027 (*Národní strategie prevence a snižování škod spojených se závislostním chováním 2019–2027*) https://www.drogy-info.cz/data/obj_files/33017/1045/National_strategy_2019_2027_fin_rev3.pdf | B7.2 | National Addiction Strategy | Prevention | People who inject drugs | Direct | Adopted | Facilitates |
| Czech Republic |  | C4 | National Addiction Strategy | Prevention | People who inject drugs, All drug users | Indirect | Adopted | Facilitates |
| Czech Republic |  | C6.2 | National Addiction Strategy | Prevention | People who inject drugs | Direct | Adopted | Facilitates |
| Czech Republic | Recommended practice of care for HIV-infected adults and post-exposure prophylaxis of HIV infection *(Doporučený postup péče o dospělé infikované HIV a postexpoziční profylaxe infekce HIV)* https://infektologie.cz/DPHIV19.htm | A1 | HIV Guidelines. Chapter 5 | Treatment, Prevention | PLHIV | Direct | Adopted | Facilitates |
| Czech Republic |  | A2 | HIV Guidelines. Chapter 5 | Treatment, Prevention | PLHIV | Direct | Adopted | Facilitates |
| Czech Republic |  | A3 | HIV Guidelines. Chapter 7 | Treatment, Prevention | PLHIV | Direct | Adopted | Facilitates |
| Czech Republic |  | A5 | HIV Guidelines. Section 2 | Prevention, Treatment, Care | PLHIV | Direct | Adopted | Facilitates |
| Czech Republic |  | A10 | HIV Guidelines | Treatment, Prevention, Care | PLHIV | Direct | Adopted | Facilitates |
| Czech Republic |  | A11 | HIV Guidelines. Chapter 8 | Treatment, Prevention, Care | PLHIV | Direct | Adopted | Facilitates |
| Czech Republic |  | D2.2 | HIV Guidelines. Chapter 6 | Prevention, Treatment, Care | PLHIV | Direct | Adopted | Facilitates |
| Czech Republic | Recommended procedure for the diagnosis and treatment of nontuberculous mycobacteriosis in adults *(Doporučený postup pro diagnostiku a léčbu netuberkulózních mykobakterióz dospělých)* http://www.pneumologie.cz/upload/1583105766.7115.pdf | A9 | TB diagnostics and treatment guidelines. Section "Epidemiological characteristics" | Treatment | PLHIV | Direct | Adopted | Facilitates |
| Czech Republic | Regulation No. 40/2009 Coll. Criminal Code *(Předpis 40/2009 Sb. Zákon trestní zákoník)* https://www.zakonyprolidi.cz/cs/2009-40 | C1 | Criminal Code. Title III | Prevention, Testing, Treatment, Care | Gay men and other MSM, Transgender people | Indirect | Adopted | Facilitates |
| Czech Republic |  | C2 | Criminal Code. Title II, Part 1 | Prevention, Testing, Treatment, Care | Sex workers | Indirect | Partially adopted | Hinders |
| Czech Republic |  | C3 | Criminal Code. Title VII, Part 1 | Prevention, Testing, Treatment, Care | People who inject drugs | Indirect | Adopted | Facilitates |
| Czech Republic |  | C7 | Criminal Code. Title I, Part 3 | Prevention, Testing, Treatment, Care | PLHIV | Indirect | Partially adopted | Hinders |
| Czech Republic |  | C13 | Criminal Code. Title III | Prevention, Testing, Treatment, Care | General population | Indirect | Adopted | Facilitates |
| Czech Republic | Resolution No. 2/1993 Coll. Resolution of the Presidium of the Czech National Council on the announcement of the CHARTER OF FUNDAMENTAL RIGHTS AND FREEDOMS as part of the constitutional order of the Czech Republic *(Usnesení č. 2/1993 Sb. Usnesení předsednictva České národní rady o vyhlášení LISTINY ZÁKLADNÍCH PRÁV A SVOBOD jako součástí ústavního pořádku České republiky)* https://www.zakonyprolidi.cz/cs/1993-2 | C8.2 | Charter of Fundamental Rights and Freedoms. Chapter 1, Article 3 | Prevention, Testing, Treatment, Care | Gay men and other MSM, Transgender people, General population | Indirect | Adopted | Facilitates |
| Czech Republic |  | C11 | Charter of Fundamental Rights and Freedoms. Chapter 4, Article 31 | Prevention, Testing, Treatment, Care | General population | Indirect | Adopted | Facilitates |
| Czech Republic |  | C12.1 | Charter of Fundamental Rights and Freedoms. Chapter 4, Article 33 | Prevention | General population | Indirect | Adopted | Facilitates |
| Czech Republic | Resolution of the Government of the Czech Republic No. 809 of December 9, 1998 on improving the protection of human rights in the Czech Republic *(usnesením vlády ČR č. 809 ze dne 9. prosince 1998 ke zlepšení ochrany lidských práv v České republice)* https://albatros.odok.cz/usneseni/usneseni_webtest.nsf/0/3B62DC3C926E5647C12571B6006EC9D2 | C10 | Council of the Government of the Czech Republic for Human Rights | Treatment, Care | General population | Indirect | Adopted | Facilitates |
| Czech Republic | The Strategic Framework for the Development of Health Care in the Czech Republic until 2030, 'Health 2030' *(Strategický rámec rozvoje péče o zdraví v České republice do roku 2030, 'Strategický rámec Zdraví 2030')* https://zdravi2030.mzcr.cz/zdravi-2030-strategicky-ramec.pdf | B13 | Strategic Framework for the Development of Health Care. Section 1.2 | Prevention | General population | Direct | Adopted | Facilitates |
| Estonia | Act on Narcotic Drugs and Psychotropic Substances and Precursors thereof *(Narkootiliste ja psühhotroopsete ainete ning nende lähteainete seadus)* https://www.riigiteataja.ee/en/eli/ee/523012020001/consolide/current | C3.1 | Act on Narcotic Drugs and Psychotropic Substances. Chapter 3, Section 15 | Prevention, Testing, Treatment, Care | People who inject drugs | Indirect | Not adopted | Hinders |
| Estonia | Basic Schools and Upper Secondary Schools Act *(Põhikooli- ja gümnaasiumiseadus)* https://www.riigiteataja.ee/en/eli/ee/Riigikogu/act/515032023010/consolide | C12 | Act on Narcotic Drugs and Psychotropic Substances.Chapter 2 | Prevention | General population | Indirect | Adopted | Facilitates |
| Estonia | Common Requirements for Medical Operations for Gender Reassignment *(Soovahetuse arstlike toimingute ühtsed nõuded)* https://www.riigiteataja.ee/en/eli/509072019001/consolide | C9 | Common Requirements for Medical Operations for Gender Reassignment | Prevention, Testing, Treatment, Care | Transgender people | Indirect | Adopted | Facilitates |
| Estonia | Equal Treatment Act *(Võrdse kohtlemise seadus)* https://www.riigiteataja.ee/en/eli/530102013066/consolide | C8.3 | Equal Treatment Act. Chapter 1, Section 1 | Prevention, Testing, Treatment, Care | General population | Indirect | Adopted | Facilitates |
| Estonia | Family Law Act *(Perekonnaseadus)* https://www.riigiteataja.ee/akt/111012023011 | C16 | Family Law Act. Chapter 1, Section 1 | Prevention, Testing, Treatment, Care | Gay men and other MSM, Transgender people, Lesbians | Indirect | Not adopted | Hinders |
| Estonia | Foundations Act *(Sihtasutuste seadus)* https://www.riigiteataja.ee/akt/123122022031 | C14.1 | Foundations Act. Chapter 2, Section 11 | Prevention, Testing, Treatment, Care | General population | Indirect | Partially adopted | Facilitates |
| Estonia | Health Insurance Act *(Ravikindlustuse seadus)* https://www.riigiteataja.ee/akt/120062022053 | D6 | Health Insurance Act. Chapter 1, Section 2 | Prevention, Treatment | General population | Indirect | Not adopted | Hinders |
| Estonia | National curriculum for basic schools *(Põhikooli riiklik õppekava)* https://www.riigiteataja.ee/en/eli/524092014014/consolide | B8.1 | National curriculum for basic schools. Chapter 2, Division 7, Section 13 | Prevention | General population | Indirect | Adopted | Facilitates |
| Estonia | National curriculum for upper secondary schools https://www.riigiteataja.ee/en/eli/524092014009/consolide | B8.2 | National curriculum for upper secondary schools. Chapter 2, Division 4, Section 8 | Prevention | General population | Indirect | Adopted | Facilitates |
| Estonia | National HIV Action Plan 2017–2025 *(Riiklik HIV tegevuskava aastateks 2017–2025)* https://www.sm.ee/sites/default/files/content-editors/Tervishoid/rahvatervis/hiv_riiklik_tegevuskava_2017_2025.pdf | A8 | National HIV Action Plan. Section 3 | Treatment | Incarcerated people | Direct | Adopted | Facilitates |
| Estonia |  | B6.2 | National HIV Action Plan. Section 3 | Prevention | Gay men and other MSM, Sex workers, People who inject drugs, Incarcerated people | Direct | Adopted | Facilitates |
| Estonia |  | B7 | National HIV Action Plan. Section 3 | Prevention | People who inject drugs | Direct | Adopted | Facilitates |
| Estonia |  | B9 | National HIV Action Plan. Section 3 | Prevention, Treatment | Sex workers | Direct | Adopted | Facilitates |
| Estonia |  | B10 | National HIV Action Plan. Section 3 | Prevention, Treatment, Testing | Gay men and other MSM | Direct | Adopted | Facilitates |
| Estonia |  | B11 | National HIV Action Plan. Section 3 | Prevention | Incarcerated people | Direct | Partially adopted | Facilitates |
| Estonia |  | C5 | National HIV Action Plan. Section 3 | Prevention | People who inject drugs | Direct | Adopted | Facilitates |
| Estonia |  | C6 | National HIV Action Plan. Section 3 | Prevention | People who inject drugs | Direct | Adopted | Facilitates |
| Estonia |  | D1 | National HIV Action Plan | Prevention, Testing, Treatment, Care | Gay men and other MSM, Sex workers, People who inject drugs, Incarcerated people | Direct | Adopted | Facilitates |
| Estonia |  | D2 | National HIV Action Plan. Section 4 | Prevention, Testing, Treatment, Care | Gay men and other MSM, Sex workers, People who inject drugs, Incarcerated people | Direct | Adopted | Facilitates |
| Estonia | Non-profit Associations Act *(Mittetulundusühingute seadus)* https://www.riigiteataja.ee/en/eli/ee/Riigikogu/act/501022023003/consolide | C14.2 | Non-profit Associations Act. Chapter 2, Section 8 | Prevention, Testing, Care | General population | Indirect | Partially adopted | Facilitates |
| Estonia | Patents Act *(Patendiseadus)* https://www.riigiteataja.ee/en/eli/511112013016/consolide | D7 | Patents Act. Chapter X |  | General population | Indirect | Partially adopted | Facilitates |
| Estonia | Penal Code *(Karistusseadustik)* https://www.riigiteataja.ee/en/eli/ee/Riigikogu/act/520032023010/consolide | C1 | The Penal Code contains no mention of same-sex relations or sexual orientation as a criminal offense. | Prevention, Testing, Treatment, Care | Gay men and other MSM | Indirect | Adopted | Facilitates |
| Estonia |  | C2 | Penal Code. Chapter 6, Article 133 | Prevention, Testing, Treatment, Care | Sex workers | Indirect | Partially adopted | Facilitates |
| Estonia |  | C3.2 | Penal Code. Chapter 12, Subchapter 1, Section 183 | Prevention, Testing, Treatment, Care | People who inject drugs | Indirect | Not adopted | Hinders |
| Estonia |  | C7 | Penal Code. Chapter 9, Subchapter 2, Sections 118 and 119 | Prevention, Testing, Treatment, Care | PLHIV | Indirect | Partially adopted | Hinders |
| Estonia |  | C8.1 | Penal Code. Chapter 10 | Prevention, Testing, Treatment, Care | Gay men and other MSM, Transgender people, General population | Indirect | Adopted | Facilitates |
| Estonia |  | C8-B | Penal Code. Chapter 10 | Prevention, Testing, Treatment, Care | Gay men and other MSM | Indirect | Adopted | Facilitates |
| Estonia |  | C8-E | Penal Code. Chapter 10 | Prevention, Testing, Treatment, Care | Migrants | Indirect | Adopted | Facilitates |
| Estonia | Population health development plan 2020-2030 *(Rahvastiku tervise arengukava 2020-2030)* https://www.sm.ee/media/3054/download | B13 | Population health development plan. Chapter 6 | Prevention, Testing, Treatment | General population | Direct | Adopted | Facilitates |
| Estonia |  | C4 | Population health development plan. Chapter 6 | Prevention | People who inject drugs, All drug users | Indirect | Adopted | Facilitates |
| Estonia | State Budget Strategy 2021-2024 (*Riigi Eelarvestrateegia 2021-2024*) https://www.fin.ee/en/media/2273/download | D4 | State Budget Strategy. Section 2.2 | Prevention, Testing, Treatment, Care | PLHIV | Direct | Adopted | Facilitates |
| Estonia | The Constitution of the Republic of Estonia *(Eesti Vabariigi põhiseadus)* https://www.riigiteataja.ee/en/eli/ee/rhvv/act/530122020003/consolide | C8.2 | The Constitution. Chapter 2, Article 12 | Prevention, Testing, Treatment, Care | Gay men and other MSM, Transgender people, General population | Indirect | Adopted | Facilitates |
| Estonia |  | C10 | The Constitution. Chapter 12 | Treatment, Care | General population | Indirect | Adopted | Facilitates |
| Estonia |  | C11 | The Constitution. Chapter 2, Section 28 | Prevention, Testing, Treatment, Care | General population | Indirect | Adopted | Facilitates |
| Estonia | Treatment guidelines: HIV testing treatment guide *(Ravijuhendid: HIV-testimise ravijuhend)* https://www.ravijuhend.ee/tervishoiuvarav/juhendid-gp/6/hiv-testimise-ravijuhend | B1 | HIV testing guideline | Testing | General population | Direct | Adopted | Facilitates |
| Estonia |  | B2 | HIV testing guideline. Treatment Manual Recommendations Section | Testing | General population | Direct | Adopted | Facilitates |
| Estonia |  | B3 | HIV testing guideline. Treatment Manual Recommendations Section | Testing, Treatment | PLHIV and their sexual partners | Direct | Partially adopted | Hinders |
| Estonia |  | B4 | HIV testing guideline | Testing | Gay men and other MSM, Sex workers, People who inject drugs, General population | Direct | Adopted | Facilitates |
| Estonia | Treatment guidelines: Management of pulmonary and extrapulmonary tuberculosis *(Ravijuhendid: Kopsu- ja kopsuvälise tuberkuloosi käsitlus)* https://www.ravijuhend.ee/tervishoiuvarav/juhendid/200/kopsu-ja-kopsuvalise-tuberkuloosi-kasitlus | A9 | Tuberculosis treatment guidelines. Laboratory diagnostics of pulmonary and extrapulmonary tuberculosis Section | Treatment | PLHIV | Direct | Adopted | Facilitates |
| Estonia | Treatment guidelines: Pre- and post-exposure prophylaxis of HIV infection and treatment of HIV-positive individuals *(Ravijuhendid: HIV-infektsiooni kokkupuute-eelne ja -järgne profülaktika ning HIV-positiivsete isikute ravi)* https://www.ravijuhend.ee/tervishoiuvarav/juhendid/195/hiv-infektsiooni-kokkupuute-eelne-ja-jargne-profulaktika-ning-hiv-positiivsete-isikute-ravi | A1 | HIV treatment guidelines. ARV treatment preparations Section | Treatment, Prevention | PLHIV | Direct | Adopted | Facilitates |
| Estonia |  | A2 | HIV treatment guidelines. ARV treatment preparations Section | Treatment, Prevention | PLHIV | Direct | Adopted | Facilitates |
| Estonia |  | A3 | HIV treatment guidelines. ARV treatment preparations Section | Treatment, Prevention, Care | PLHIV | Direct | Adopted | Facilitates |
| Estonia |  | A4 | HIV treatment guidelines. ARV treatment preparations Section | Treatment | PLHIV | Direct | Not adopted | Hinders |
| Estonia |  | A5 | HIV treatment guidelines. ARV treatment monitoring Section | Treatment, Care, Prevention | PLHIV | Direct | Adopted | Facilitates |
| Estonia |  | A10 | HIV treatment guidelines | Treatment | PLHIV | Direct | Adopted | Facilitates |
| Estonia |  | B6.1 | HIV treatment guidelines. PrEP Section | Prevention | General population | Direct | Adopted | Facilitates |
| Estonia |  | D3 | HIV treatment guidelines. ARV treatment preparations Section | Treatment, Prevention | PLHIV | Direct | Not adopted | Hinders |
| Estonia |  | D5 | HIV treatment guidelines. ARV treatment preparations Section | Treatment, Prevention | PLHIV | Direct | Adopted | Facilitates |
| France | Annex 31-2 to Annex III of the Public Health Code (*Annexe 31-2 à L'Annexe III du Code de la Santé Publique*) https://www.legifrance.gouv.fr/codes/article_lc/LEGIARTI000006908109 | C4.1 | Public Health Code. Annex 31-2 to Annex III | Prevention | People who inject drugs, All illicit drug users | Direct | Adopted | Facilitates |
| France |  | C5 | Public Health Code | Prevention | People who inject drugs | Direct | Adopted | Facilitates |
| France | Article L.3421-1 of the Public Health Code (*Article L.3421-1 du Code de la Santé Publique*) https://www.santepubliquefrance.fr/determinants-de-sante/drogues-illicites/articles/que-prevoit-la-loi-en-cas-de-consommation-de-stupefiant | C3 | Public Health Code. Article L.3421-1 | Prevention, Testing, Treatment, Care | People who inject drugs, All illicit drug users | Indirect | Not adopted | Hinders |
| France | Asylum Access to Care https://www.immigration.interieur.gouv.fr/Asile/L-acces-aux-soins | A7.1 | Asylum Access to Care | Treatment, Care | Migrants and other foreign residents | Indirect | Partially adopted | Facilitates |
| France | Constitution of the French Republic of 1946 (*Constitution de la République Française 1946*) https://www.elysee.fr/la-presidence/la-constitution-du-27-octobre-1946# | C11 | The Constitution. Paragraph 11 | Prevention, Treatment, Care | General population | Indirect | Adopted | Facilitates |
| France | Decree No. 2017-281 of March 2, 2017 approving the national risk reduction benchmark aimed at prostitutes and supplementing the public health code (*Décret no 2017-281 du 2 mars 2017 approuvant le référentiel national de réduction des risques en direction des personnes prostituées et complétant le code de la santé publique*) https://www.legifrance.gouv.fr/download/pdf?id=PsbADs9fLORgkRZRI3VTcN2zup93I6QTVIOrDQLksWk= | B9.2 | Decree supplementing the public health code | Prevention, Testing | Sex workers | Direct | Adopted | Facilitates |
| France | Decree No. 2017-450 of 29 March 2017 on the procedures for changing the first name and changing the designation of sex in the civil status https://www.legifrance.gouv.fr/loda/id/LEGIARTI000034321559 | C9 | Decree on the procedures for changing the first name and changing the designation of sex in the civil status | Prevention, Testing, Treatment, Care | Transgender people | Indirect | Adopted | Facilitates |
| France | HIV Diagnosis (D*ępistage de VIH L'Assuarnce Maladie*) https://www.ameli.fr/assure/sante/themes/vih/depistage | D6.1 | HIV Diagnosis | Prevention | General population | Direct | Adopted | Facilitates |
| France | HIV management- Expert panel Recommendations. Support for children and teenagers infected with HIV (*Prise en charge des enfants et adolescents infectés par le VIH*) https://cns.sante.fr/wp-content/uploads/2018/02/experts-vih_pediatrie.pdf | A6 | HIV management- Expert panel Recommendations | Treatment, Care | Children with HIV | Direct | Adopted | Facilitates |
| France | HIV Management- Expert Panel Recommendations. Follow-up of Persons Living with HIV (*Prise en charge médicale des personnes vivant avec le VIH. Suivi de l’adulte vivant avec le VIH et organisation des soins*) https://cns.sante.fr/wp-content/uploads/2018/05/experts-vih_suivi.pdf | A3.2 | HIV management- Expert panel Recommendations | Care | PLHIV | Direct | Adopted | Facilitates |
| France |  | A4 | HIV management- Expert panel Recommendations | Treatment, Care | PLHIV | Direct | Adopted | Facilitates |
| France |  | A7.3 | HIV management- Expert panel Recommendations | Prevention, Testing, Treatment, Care | Migrants and other foreign residents | Direct | Partially adopted | Facilitates |
| France |  | A10.2 | HIV management- Expert panel Recommendations | Care | All PLHIV | Direct | Adopted | Facilitates |
| France |  | A11.2 | HIV management- Expert panel Recommendations | Treatment, Care | Migrants and other foreign residents, All WLHIV | Direct | Adopted | Facilitates |
| France | HIV Management – Expert Panel Recommendations. Infections in adults: prophylaxis and curative treatments (*Infections chez l’adulte: prophylaxis et traitements curatifs*) https://cns.sante.fr/wp-content/uploads/2018/05/experts-vih_infections.pdf | A9 | HIV management- Expert panel Recommendations | Care | Migrants and other foreign residents, All PLHIV | Direct | Adopted | Facilitates |
| France | HIV Management – Expert Panel Recommendations. Initiation of first antiretroviral treatment. (*Prise En Charge Médical Des Personnes Vivant Avec Le VIH. Initiation d'un premier traitement antirétroviral (avril 2018*)) https://cns.sante.fr/wp-content/uploads/2017/01/experts-vih_initiation.pdf | A1 | HIV management- Expert panel Recommendations | Treatment, Prevention | PLHIV | Direct | Adopted | Facilitates |
| France |  | A2 | HIV management- Expert panel Recommendations | Treatment, Prevention | PLHIV | Direct | Adopted | Facilitates |
| France |  | A3.1 | HIV management- Expert panel Recommendations | Treatment, Prevention | PLHIV | Direct | Adopted | Facilitates |
| France |  | A10.1 | HIV management- Expert panel Recommendations | Care, Treatment | All PLHIV | Direct | Adopted | Facilitates |
| France |  | A11.1 | HIV management- Expert panel Recommendations | Treatment, Care | All WLHIV | Direct | Adopted | Facilitates |
| France | HIV Management – Expert Panel Recommendations. Optimization of antiretroviral treatment in a situation of virological success (*Optimisation d'un traitement antirétroviral en situation de succès virologique*) https://cns.sante.fr/wp-content/uploads/2017/01/experts-vih_optimisation.pdf | A10.3 | HIV management- Expert panel Recommendations | Treatment | All PLHIV | Direct | Adopted | Facilitates |
| France | Human Rights League (*Ligue des droits de l'homme - LDH*) https://www.ldh-france.org | C10.2 | Human Rights League | Prevention, Testing, Treatment, Care | General population | Indirect | Adopted | Facilitates |
| France | HIV-STI Public Health Bulletin (*Bulletin de santé publique VIH-IST*) https://www.santepubliquefrance.fr/maladies-et-traumatismes/infections-sexuellement-transmissibles/vih-sida/documents/bulletin-national/bulletin-de-sante-publique-vih-ist.-decembre-2022 | D9 | HIV-STI Public Health Bulletin | Testing | Gay men and other MSM, Sex workers, Transgender people, People who inject drugs, General population | Direct | Partially adopted | Facilitates |
| France | Interministerial strategy of mobilization against addictive behaviors 2023-2027 (*Stratégie Interministérielle de mobilization contre les conduites addictives 2023-2027*) https://www.drogues.gouv.fr/sites/default/files/2023-03/SIMCA%202023-2027_0.pdf | C4.2 | Interministerial strategy of mobilization against addictive behaviors. Strategic Orientation 6 | Prevention | People who inject drugs, All drug users | Direct | Adopted | Facilitates |
| France | Issue and implementation of sexuality education (*Problématique et mise en œuvre de l'éducation à la sexualité*) https://www.education.gouv.fr/education-la-sexualite-1814 | B8.2 | Issue and implementation of sexuality education Program | Prevention | School children in general | Indirect | Adopted | Facilitates |
| France | Law No. 2008-496 of 27 May 2008 containing various provisions for adaptation to Community law in the field of anti-discrimination (*Loi n° 2008-496 du 27 mai 2008 portant diverses dispositions d'adaptation au droit communautaire en matière de lutte contre les discriminations*) https://www.legifrance.gouv.fr/loda/id/JORFTEXT000018877783/ | C8 | Anti-discrimination Law | Prevention, Testing, Treatment, Care | Gay men and other MSM, Transgender people, Sex workers, People who inject drugs, All PLHIV | Indirect | Adopted | Facilitates |
| France |  | C8-B | Anti-discrimination Law | Prevention, Testing, Treatment, Care | Gay men and other MSM | Indirect | Adopted | Facilitates |
| France |  | C8-D | Anti-discrimination Law | Prevention, Testing, Treatment, Care | Transgender people | Indirect | Adopted | Facilitates |
| France |  | C8-E | Anti-discrimination Law | Prevention, Testing, Treatment, Care | Migrants and other foreign residents | Indirect | Adopted | Facilitates |
| France | Law No. 2013-404 of May 17, 2013, opening marriage to same-sex couples https://www.legifrance.gouv.fr/loda/id/JORFTEXT000027414540/ | C16 | Law opening marriage to same-sex couples | Prevention, Testing, Treatment, Care | Gay men and other MSM, Transgender people, Lesbians | Indirect | Adopted | Facilitates |
| France | Law of 13 April 2016 to strengthen the fight against the prostitution system and to support prostitutes (*Loi du 13 avril 2016 pour renforcer la lutte contre le système prostitutionnel et pour soutenir les personnes prostituées*) https://www.legifrance.gouv.fr/loda/id/JORFTEXT000032396046 | C2 | Law to strengthen the fight against the prostitution system and to support prostitutes. Chapter V | Prevention, Testing, Treatment, Care | Sex workers | Indirect | Not adopted | Facilitates / Hinders |
| France | Law of 20 June 2018 on the protection of personal data. (*Loi du 20 juin 2018 relative à la protection des données personnelles*) https://www.legifrance.gouv.fr/loda/id/JORFTEXT000037085952/?isSuggest=true | D8 | Law on the protection of personal data | Prevention, Testing, Treatment, Care | Gay men and other MSM, Sex workers, Transgender people, General population | Indirect | Partially adopted | Facilitates |
| France | Medical care of people living with HIV. Prevention and screening (*Prise En Charge Médical Des Personnes Vivant Avec Le VIH. Prevention et dépistage*) https://cns.sante.fr/wp-content/uploads/2018/04/experts-vih_prevention-depistage.pdf | B1 | Guidelines on medical care of people living with HIV | Prevention, Testing | Gay men and other MSM, People who inject drugs, Migrants and other foreign residents, Heterosexual women and men born in sub-Saharan Africa and in the French Territories of America (TFA) | Direct | Adopted | Facilitates |
| France |  | B2 | Guidelines on medical care of people living with HIV | Prevention, Testing | Gay men and other MSM, People who inject drugs, Migrants and other foreign residents, Heterosexual women and men born in sub-Saharan Africa and in the French Territories of America (TFA) | Direct | Adopted | Facilitates |
| France |  | B6 | Guidelines on medical care of people living with HIV | Prevention | Gay men and other MSM, Transgender people, People who inject drugs, Sex workers, Serodiscordant couples, adolescent at high risk of infection | Direct | Adopted | Facilitates |
| France | National Health Strategy 2018-2022 (*Stratégie nationale de santé 2018-2022*) https://sante.gouv.fr/IMG/pdf/dossier_sns_2017_vdefpost-consult.pdf | B13 | National Health Strategy. Part A | Prevention, Testing | General population but especially young people and key populations - though not specially identified by group | Direct | Adopted | Facilitates |
| France | National Plan to Combat HIV/AIDS and STI 2010-2014 (*Plan national de lutte contre le VIH/SIDA et les IST 2010-2014*) https://sante.gouv.fr/IMG/pdf/plan_national_lutte_contre_le_VIH-SIDA_et_les_IST_2010-2014.pdf | D2 | National Plan to Combat HIV/AIDS and STI | Prevention, Testing, Treatment, Care | Gay men and other MSM, General population | Direct | Partially adopted | Facilitates |
| France |  | D4 | National Plan to Combat HIV/AIDS and STI | Prevention, Testing | Gay men and other MSM, General population | Direct | Adopted | Facilitates |
| France | National Strategy for Sexual Health. Agenda 2017-2030 (*Stratégie nationale de santé sexuelle Agenda 2017-2030*) https://sante.gouv.fr/IMG/pdf/strategie_nationale_sante_sexuelle.pdf | A7.2 | National Strategy for Sexual Health. Axis IV | Prevention, Testing, Treatment, Care | Migrants and other foreign residents | Direct | Partially adopted | Facilitates |
| France |  | B7 | National Strategy for Sexual Health | Prevention, Testing | People who inject drugs | Direct | Adopted | Facilitates |
| France |  | B8.1 | National Strategy for Sexual Health. Axis I | Prevention | Whole population, particularly young people | Indirect | Adopted | Facilitates |
| France |  | B9.1 | National Strategy for Sexual Health. Axis IV, Objective 1.4 | Prevention, Testing | Sex workers | Direct | Adopted | Facilitates |
| France |  | B10 | National Strategy for Sexual Health. Axis IV, Objective 1.1 | Prevention, Testing | Gay men and other MSM | Direct | Adopted | Facilitates |
| France |  | B11 | National Strategy for Sexual Health. Axis IV, Objective 1.4 | Prevention, Testing | Incarcerated people | Direct | Partially adopted | Facilitates |
| France |  | D1 | National Strategy for Sexual Health | Prevention, Testing, Treatment, Care | Gay men and other MSM, Sex workers, Transgender people, People who inject drugs, Incarcerated people, Migrants; Disabled people and those with chronic disease | Direct | Adopted | Facilitates |
| France | Notification to partners (*La notification aux partenaires*) https://www.has-sante.fr/jcms/p_3187510/en/la-notification-au-x-partenaire-s | B3 | Notification to partners guideline | Prevention, Testing | PLHIV and their sexual partners | Direct | Adopted | Facilitates |
| France | Penal Code (Code Pénal) https://ledroitcriminel.fr/la_legislation_criminelle/anciens_textes/code_penal_25_09_1791.htm | C1 | Penal Code decriminalize homosexual acts | Prevention, Testing, Treatment, Care | Gay men and other MSM | Indirect | Adopted | Facilitates |
| France | Penal risk incurred by people living with HIV due to unprotected sex (*Risque Pénal Encouru Par Les Personnes Vivant Avec Le VIH Du Fait De Relations Sexuelles Non Protégées*) https://www.legifrance.gouv.fr/codes/article_lc/LEGIARTI000006417651/2021-04-30 | C7 | Penal Code. Article 222-15 | Prevention, Testing, Treatment, Care | All PLHIV | Indirect | Partially adopted | Hinders |
| France | The Fight Against HIV/AIDS: An Approach, Knowledge and Practices to Serve to the Challenges of the Present (*La Lutte Contre Le VIH/SIDA: Une Démarche, des Savoirs et des Pratiques Pour Servir Aux Enjeux du Présent*) https://cns.sante.fr/wp-content/uploads/2021/07/2021-06-17_avi_fr_prise_en_charge.pdf | B4 | The Fight Against HIV/AIDS strategy | Testing | General population | Direct | Adopted | Facilitates |
| France | The French National Consultative Commission on Human Rights (*Commission nationale consultative des droits de l’homme – CNCDH*) https://www.cncdh.fr/ | C10.1 | National Consultative Commission on Human Rights | Prevention, Testing, Treatment, Care | General population | Indirect | Adopted | Facilitates |
| France | Therapeutic strategies for opioid-dependent people: the place of substitution treatment https://www.has-sante.fr/jcms/c_272358/fr/strategies-therapeutiques-pour-les-personnes-dependantes-des-opiaces-place-des-traitements-de-substitution | C6 | Therapeutic strategies for opioid-dependent people guidelines | Prevention | People who inject drugs | Direct | Adopted | Facilitates |
| France | Universal Health Protection (*L'Assurance Maladie*) https://www.ameli.fr/assure/droits-demarches/principes/protection-universelle-maladie | D5 | Universal Health Protection | Prevention, Testing, Treatment, Care | General population | Direct | Adopted | Facilitates |
| France |  | D6.2 | Universal Health Protection | Treatment | General population | Indirect | Partially adopted | Hinders |
| Germany | Act on the Execution of Imprisonment and Measures of Correction and Prevention involving Deprivation of Freedom (*Gesetz über den Vollzug der Freiheitsstrafe und der freiheitsentziehenden Maßregeln der Besserung und Sicherung (StVollzG)*) https://www.gesetze-im-internet.de/stvollzg/ | A8 | Law regulating incarceration. Article 56 | Prevention, Testing, Treatment, Care | Incarcerated people | Indirect | Adopted | Facilitates |
| Germany |  | B11 | Law regulating incarceration. Article 56 | Prevention | Incarcerated people | Indirect | Partially adopted | Facilitates |
| Germany | Asylum Seeker Benefits Act (*Asylbewerberleistungsgesetz (AsylbLG)*) https://www.gesetze-im-internet.de/asylblg/BJNR107410993.html | A7 | Asylum Seeker Benefits Act. Article 4 | Treatment, Testing | Migrants | Indirect | Not adopted | Hinders |
| Germany | Basic Law for the Federal Republic of Germany (Grundgesetz für die Bundesrepublik Deutschland) https://www.gesetze-im-internet.de/englisch_gg/englisch_gg.html | C8.1 | The Constitution. Article 3 | Prevention, Testing, Treatment, Care | General population | Indirect | Adopted | Facilitates |
| Germany | Civil Code (Bürgerliches Gesetzbuch) https://www.gesetze-im-internet.de/bgb/BJNR001950896.html | C14 | Civil Code. Paragraph 21 | Prevention, Testing, Treatment, Care | General population | Indirect | Adopted | Facilitates |
| Germany | General Equal Treatment Act (Allgemeines Gleichbehandlungsgesetz) https://www.gesetze-im-internet.de/agg/BJNR189710006.html#BJNR189710006BJNG000300000 | C8.2 | General Equal Treatment Act. Paragraph 2 | Prevention, Testing, Treatment, Care | General population | Indirect | Adopted | Facilitates |
| Germany |  | C8-A | General Equal Treatment Act. Paragraph 2 | Prevention, Testing, Treatment, Care | PLHIV | Indirect | Adopted | Facilitates |
| Germany |  | C8-B | General Equal Treatment Act. Paragraph 2 | Prevention, Testing, Treatment, Care | Gay men and other MSM | Indirect | Adopted | Facilitates |
| Germany |  | C8-C | General Equal Treatment Act. Paragraph 2 | Prevention, Testing, Treatment, Care | People who inject drugs | Indirect | Adopted | Facilitates |
| Germany |  | C8-D | General Equal Treatment Act. Paragraph 2 | Prevention, Testing, Treatment, Care | Transgender people | Indirect | Adopted | Facilitates |
| Germany |  | C8-E | General Equal Treatment Act. Paragraph 2 | Prevention, Testing, Treatment, Care | Migrants | Indirect | Adopted | Facilitates |
| Germany | German Penal Code https://www.gesetze-im-internet.de/englisch_stgb/englisch_stgb.html#p1666 | C1 | Penal Code. Paragraph 175 repealed | Prevention, Testing, Treatment, Care | Gay men and other MSM | Indirect | Adopted | Facilitates |
| Germany |  | C7 | Penal Code. Sections 223 to 225 | Prevention, Testing, Treatment, Care | PLHIV | Indirect | Partially adopted | Hinders |
| Germany | German-Austrian guidelines for HIV pre-exposure prophylaxis (*Deutsch-Österreichische Leitlinien zur HIV-Präexpositionsprophylaxe*) https://register.awmf.org/assets/guidelines/055-008l_S2k_HIV-Praeexpositionsprophylaxe_2019-01_01.pdf | B6 | German-Austrian guidelines for HIV pre-exposure prophylaxis | Prevention | Gay men and other MSM, Transgender people, People who inject drugs, General population | Direct | Adopted | Facilitates |
| Germany | HIV / AIDS: key data and trends for Germany and the federal states (HIV/AIDS: Eckdaten und Trends für Deutschland und für die Bundesländer) https://www.rki.de/DE/Content/InfAZ/H/HIVAIDS/Eckdaten/Eckdaten.html?nn=2374210 | D9 | HIV/AIDS: key data and trends | Prevention, Testing, Treatment, Care | PLHIV | Direct | Partially adopted | Facilitates |
| Germany | HIV Self-tests, Paul Ehrlich Institute https://www.pei.de/EN/newsroom/hiv-self-tests/hiv-self-tests-node.html | B2 | HIV Self-tests | Testing | General population | Direct | Adopted | Facilitates |
| Germany | Integrated Strategy for HIV, Hepatitis B and C and Other Sexually Transmitted Infections (*Strategie zur Eindämmung von HIV, Hepatitis B und C und anderen sexuell übertragbaren Infektionen*) https://www.bundesgesundheitsministerium.de/fileadmin/Dateien/5_Publikationen/Praevention/Broschueren/Strategy_HIV_HEP_STI.pdf | D1 | Integrated Strategy for HIV, Hepatitis B and C and Other Sexually Transmitted Infections | Prevention, Testing, Treatment, Care | Gay men and other MSM, Sex workers, Transgender people, People who inject drugs, Incarcerated people, Migrants, Juveniles, Pregnant Women , General population | Direct | Adopted | Facilitates |
| Germany |  | D2 | Integrated Strategy for HIV, Hepatitis B and C and Other Sexually Transmitted Infections | Prevention, Testing, Treatment, Care | Gay men and other MSM, Sex workers, Transgender people, People who inject drugs, Incarcerated people, Migrants, Juveniles, Pregnant Women , General population | Direct | Adopted | Facilitates |
| Germany | Law amending the Narcotics Act (Gesetz zur Änderung des Betäubungsmittelgesetzes) https://www.bgbl.de/xaver/bgbl/start.xav?startbk=Bundesanzeiger_BGBl&jumpTo=bgbl192s1593.pdf#__bgbl__%2F%2F*%5B%40attr_id%3D%27bgbl192s1593.pdf%27%5D__1685522006414 | C5 | Narcotics Act. Article 1, Paragraph 4 ammended | Prevention | People who inject drugs | Direct | Adopted | Facilitates |
| Germany | Law for the Protection of Persons Working in Prostitution (Prostitute Protection Act - ProstSchG) (*Gesetz zum Schutz von in der Prostitution tätigen Personen (Prostituiertenschutzgesetz - ProstSchG)*) https://www.gesetze-im-internet.de/prostschg/BJNR237210016.html | B9 | Prostitution Protection Act. Articles 7 to 20, 32 and 33 | Prevention | Sex workers | Direct | Adopted | Facilitates |
| Germany | Law on changing first names and determining gender in special cases (Gesetz über die Änderung der Vornamen und die Feststellung der Geschlechtszugehörigkeit in besonderen Fällen) https://www.gesetze-im-internet.de/tsg/BJNR016540980.html | C9 | Law on changing first names and determining gender. Paragraph 1 | Prevention, Testing, Treatment, Care | Transgender people | Indirect | Adopted | Facilitates |
| Germany | Law on civil law protection against acts of violence and snares (Gesetz zum zivilrechtlichen Schutz vor Gewalttaten und Nachstellungen) https://www.gesetze-im-internet.de/gewschg/BJNR351310001.html | C13 | Law on civil law protection against acts of violence and snares. Articles 1 and 2 | Prevention, Testing, Treatment, Care | General population | Indirect | Adopted | Facilitates |
| Germany | Law on the Legal Status and Tasks of the German Institute for Human Rights (Gesetz über die Rechtsstellung und Aufgaben des Deutschen Instituts für Menschenrechte) https://www.gesetze-im-internet.de/dimrg/BJNR119400015.html | C10 | Law on the Legal Status and Tasks of the German Institute for Human Rights. Paragraph 1 | Treatment, Care | General population | Indirect | Adopted | Facilitates |
| Germany | Law regulating the legal relationships of prostitutes- Prostitution Act (Gesetz zur Regelung der Rechtsverhältnisse der Prostituierten- Prostitutionsgesetz) https://www.gesetze-im-internet.de/prostg/BJNR398310001.html | C2 | Prostitution Act.Paragraph 1 | Prevention, Testing, Treatment, Care | Sex workers | Indirect | Adopted | Facilitates |
| Germany | Law to introduce marriage for persons of the same sex (*Gesetz zur Einführung des Rechts auf Eheschließung für Personen gleichen Geschlechts*) https://www.bgbl.de/xaver/bgbl/start.xav?startbk=Bundesanzeiger_BGBl&jumpTo=bgbl117s2787.pdf | C16 | Law to introduce marriage for persons of the same sex. Articles 1 and 2 | Prevention, Testing, Treatment, Care | Gay men and other MSM, Transgender people, Lesbians | Indirect | Adopted | Facilitates |
| Germany | Law to protect electronic patient data in the telematics infrastructure / Patient Data Protection Act - PDSG (Gesetz zum Schutz elektronischer Patientendaten in der Telematikinfrastruktur / Patientendaten-Schutz-Gesetz – PDSG) https://www.bundesgesundheitsministerium.de/fileadmin/Dateien/3_Downloads/Gesetze_und_Verordnungen/GuV/P/PDSG_bgbl.pdf | D8 | Patient Data Protection Act | Treatment | PLHIV, General Population | Indirect | Partially adopted | Facilitates |
| Germany | Narcotics Trafficking Act (Gesetz über den Verkehr mit Betäubungsmitteln) https://www.gesetze-im-internet.de/btmg_1981/ | C3 | Narcotics Trafficking Act. Section 29 | Prevention, Testing, Treatment, Care | People who inject drugs | Indirect | Not adopted | Facilitates / Hinders |
| Germany | National Health Targets (*Nationale Gesundheitsziele*) https://www.bundesgesundheitsministerium.de/themen/gesundheitswesen/gesundheitsziele.html | B13 | National Health Targets |  | General population | Indirect | Partially adopted | N/A |
| Germany | National Strategy on Drug and Addiction Policy (*Nationale Strategie zur Drogen- und Suchtpolitik*) https://www.bundesdrogenbeauftragter.de/assets/EN/Drug_Commissioner_of_the_Federal_Government__Germany__2012__National_Strategy_on_Drug_and_Addiction_Policy.pdf | B7 | National Strategy on Drug and Addiction Policy | Prevention | People who inject drugs | Direct | Adopted | Facilitates |
| Germany |  | C4 | National Strategy on Drug and Addiction Policy | Prevention | People who inject drugs, All drug users | Direct | Adopted | Facilitates |
| Germany | Ordinance on the prescription, dispensing and proof of whereabouts of narcotics (narcotics prescription ordinance - BtMVV) (*Verordnung über das Verschreiben, die Abgabe und den Nachweis des Verbleibs von Betäubungsmitteln (Betäubungsmittel-Verschreibungsverordnung - BtMVV)*) https://www.gesetze-im-internet.de/btmvv_1998/BJNR008000998.html | C6 | Narcotics prescription ordinance. Articles 5, 5a, and 5b. | Prevention | People who inject drugs | Direct | Adopted | Facilitates |
| Germany | Patent Law (Patentgesetz) https://www.gesetze-im-internet.de/patg/BJNR201170936.html | D7 | Patent Law. Section 24 | Treatment | PLHIV | Indirect | Partially adopted | Facilitates |
| Germany | Social Code (SGB) Twelfth Book (XII) - Social Assistance (*Sozialgesetzbuch (SGB) Zwölftes Buch (XII) - Sozialhilfe*) https://www.gesetze-im-internet.de/sgb_12/ | A12 | Social Security Code. Book XII | Care | PLHIV, other people with disabilities and/or economically vulnerable | Indirect | Adopted | Facilitates |
| Germany | Statutory health insurance (Gesetzliche Krankenversicherung) https://www.bundesgesundheitsministerium.de/gkv.html | D5 | Statutory health insurance | Prevention, Testing, Treatment, Care | General population | Direct | Adopted | Facilitates |
| Germany |  | D6 | Statutory health insurance | Prevention, Testing, Treatment, Care | General population | Indirect | Adopted | Facilitates |
| Germany (Berlin) | Action Program Health (*Aktionsprogramm Gesundheit*) https://www.parlament-berlin.de/ados/17/GesSoz/vorgang/gs17-0182-v-SenGesSoz%20Aktionsprogramm%20Gesundheit.pdf | B13 | Action Program Health | Testing, Prevention | Gay men and other MSM, People who inject drugs, | Direct | Adopted | Facilitates |
| Germany (Berlin) | Cooperation agreement for the implementation of the integrated health and care program for the period January 1st, 2021 December 31st, 2025 (*Kooperationsvereinbarung zur Umsetzung des Integrierten Gesundheits- und Pflege-Programms für den Zeitraum 01.01.2021 - 31.12.2025*) https://www.berlin.de/sen/gesundheit/_assets/themen/gesundheitsfoerderung-und-praevention/integriertes-gesundheitsprogramm/kooperationsvereinbarung-igpp.pdf?ts=1666280146 | C14 | Cooperation agreement for the implementation of the integrated health and care program | Prevention | Vulnerable population | Indirect | Adopted | Facilitates |
| Germany (Berlin) | Framework concept for the prevention of HIV/AIDS, hepatitis and sexually transmitted infections as well as for the care of people with HIV/AIDS and/or chronic hepatitis infections in Berlin (*Rahmenkonzept zur Prävention von HIV/Aids, Hepatitis- und sexuell übertragbaren Infektionen sowie zur Versorgung von Menschen mit HIV/Aids und/oder chronischen Hepatitisinfektionen in Berlin*) https://www.berlin.de/sen/gesundheit/_assets/themen/hiv-aids-sexuell-uebertragbare-infektionen-und-hepatitiden/rosenbrock_entwicklungskonzept_komlett_1_.pdf | D1 | Framework concept for the prevention of HIV/AIDS, hepatitis and STIs | Prevention, Treatment, Testing, Care | Gay men and other MSM, Sex workers, People who inject drugs, Incarcerated people, Migrants, General population, youth and young adults, people from high-prevalence countries. | Direct | Adopted | Facilitates |
| Germany (Berlin) | Guidelines for addiction prevention in the state of Berlin. (*Leitlinien für die Suchtprävention im Land Berlin*) https://www.berlin.de/lb/drogen-sucht/_assets/service/leitlinien.pdf | C4 | Guidelines for addiction prevention |  | Juveniles, General population. | Indirect | Adopted | Hinders |
| Germany (Berlin) | School Act for the State of Berlin - (School Act - SchulG) (*Schulgesetz für das Land Berlin - (Schulgesetz – SchulG)*) https://www.schulgesetz-berlin.de/berlin/schulgesetz.php | B8 | School Act for the State of Berlin | Prevention | Juveniles, Children | Indirect | Adopted | Facilitates |
| Germany (Berlin) | Syringe vending machines in Berlin (*Sprizenautomaten in Berlin*) https://www.berlin.de/ba-mitte/politik-und-verwaltung/service-und-organisationseinheiten/qualitaetsentwicklung-planung-und-koordination-des-oeffentlichen-gesundheitsdienstes/suchthilfekoordination/spritzenautomat_flyer_fixpunkt.pdf | C5 | Syringe vending machines | Prevention | People who inject drugs | Direct | Adopted | Facilitates |
| Italy | Agreement, pursuant to article 8, paragraph 6, of the law of 5 June 2003, n. 131, between the Government, the Regions and the autonomous Provinces of Trento and Bolzano and the local Autonomies on the document containing «HIV infection and detention» (*Intesa, ai sensi dell'articolo 8, comma 6, della legge 5 giugno 2003, n. 131, tra il Governo, le Regioni e le Province autonome di Trento e Bolzano e le Autonomie locali sul documento recante «Infezione da HIV e detenzione»*) https://www.trovanorme.salute.gov.it/norme/dettaglioAtto?id=42045 | A8 | HIV infection and detention. Sections 3, 5 and 7 | Testing, Treatment, Care | Incarcerated people | Direct | Adopted | Facilitates |
| Italy | Agreement, pursuant to article 8, paragraph 6, of the law of 5 June 2003, n. 131, between the Government, the Regions and the Autonomous Provinces of Trento and Bolzano concerning the "Consensus document on the offer policies and methods of carrying out the HIV test in Italy" (*Intesa, ai sensi dell'articolo 8, comma 6, della legge 5 giugno 2003, n. 131, tra il Governo, le Regioni e le Province autonome di Trento e Bolzano concernente «Documento di consenso sulle politiche di offerta e le modalita' di esecuzione del test per HIV in Italia»*) https://www.gazzettaufficiale.it/eli/id/2011/08/18/11A11001/sg | B1 | Consensus document on the offer policies and methods of carrying out the HIV test | Testing | People who inject drugs, Incarcerated people, General population | Direct | Adopted | Facilitates |
| Italy |  | B5 | Consensus document on the offer policies and methods of carrying out the HIV test. Section 2.4 | Testing | Minors | Direct | Not adopted | Hinders |
| Italy | Constitution of the Italian Republic (*Costituzione della Repubblica Italiana*) https://www.senato.it/documenti/repository/istituzione/costituzione_inglese.pdf | C8 | The Constitution | Prevention, Testing, Treatment, Care | General population | Indirect | Adopted | Facilitates |
| Italy |  | C11 | The Constitution. Article 32 | Prevention, Testing, Treatment, Care | General population | Indirect | Adopted | Facilitates |
| Italy |  | C12.1 | The Constitution. Articles 2, 3 and 34 |  | General population | Indirect | Adopted | Facilitates |
| Italy |  | C14.1 | The Constitution. Article 18 | Prevention, Testing, Treatment, Care | General population | Indirect | Adopted | Facilitates |
| Italy | Decision 8 May 2023 Reimbursement regime and price, following new therapeutic indications, of the medicine for human use «Emtricitabine/Tenofovir Disoproxil Mylan». (Decision n. 349/2023). *Regime di rimborsabilita' e prezzo, a seguito di nuove indicazioni terapeutiche, del medicinale per uso umano «Emtricitabina/Tenofovir Disoproxil Mylan». (Determina n. 349/2023)*) https://www.gazzettaufficiale.it/atto/serie_generale/caricaDettaglioAtto/originario?atto.dataPubblicazioneGazzetta=2023-05-18&atto.codiceRedazionale=23A02836&elenco30giorni=false | B6.1 | Decision on reimbursement regime and price | Prevention | Adults with high-risk practices, as defined in the policy. | Direct | Adopted | Facilitates |
| Italy | Decree March 31, 2008 Establishment of the surveillance system of new diagnoses of HIV infections.(*Decreto 31 marzo 2008 Istituzione del sistema di sorveglianza delle nuove diagnosi di infezioni da HIV*) https://www.gazzettaufficiale.it/atto/serie_generale/caricaDettaglioAtto/originario?atto.dataPubblicazioneGazzetta=2008-07-28&atto.codiceRedazionale=08A05276&elenco30giorni=false | D9 | Establishment of the surveillance system of new diagnoses of HIV infections | Prevention, Testing, Treatment, Care | General population | Direct | Partially adopted | Hinders |
| Italy | Decree of the President of the Republic No. 309 of 9 October 1990 Consolidated text of the laws concerning the regulation of narcotics and psychotropic substances, prevention, treatment and rehabilitation of the related states of drug addiction (*Decreto el Presidente della Repubblica 9 ottobre 1990, n. 309 Testo unico delle leggi in materia di disciplina degli stupefacenti e sostanze psicotrope, prevenzione, cura e riabilitazione dei relativi stati di tossicodipendenza)* https://www.normattiva.it/atto/caricaDettaglioAtto?atto.dataPubblicazioneGazzetta=1990-10-31&atto.codiceRedazionale=090G0363&atto.articolo.numero=0&atto.articolo.sottoArticolo=1&atto.articolo.sottoArticolo1=10&qId=e231e4a6-f0b3-4ffb-bff4-4e95451cf86d&tabID=0.29563456497896046&title=lbl.dettaglioAtto | C3 | Legal framework surrounding drugs. Articles 73 and 75 | Prevention, Testing, Treatment, Care | People who inject drugs | Indirect | Not adopted | Hinders |
| Italy | Italian Guidelines on the use of Antiretroviral Therapy and the Diagnostic-Clinical Management of People Infected with HIV (*Linee Guida Italiane sull’utilizzo della Terapia Antiretrovirale e la gestione diagnostico-clinica delle persone con infezione da HIV-1*) https://www.salute.gov.it/imgs/C_17_pubblicazioni_2696_allegato.pdf | A1 | Guidelines on the use of Antiretroviral Therapy and the Diagnostic-Clinical Management of People Infected with HIV. Section 1 | Treatment, Prevention | People living with HIV, Partners of PLHIV | Direct | Adopted | Facilitates |
| Italy |  | A3 | Guidelines on the use of Antiretroviral Therapy and the Diagnostic-Clinical Management of People Infected with HIV. Section 1 | Treatment, Prevention, Care | People living with HIV, Partners of PLHIV | Direct | Adopted | Facilitates |
| Italy |  | A4 | Guidelines on the use of Antiretroviral Therapy and the Diagnostic-Clinical Management of People Infected with HIV. Section 2 | Treatment | PLHIV | Direct | Adopted | Facilitates |
| Italy |  | A5 | Guidelines on the use of Antiretroviral Therapy and the Diagnostic-Clinical Management of People Infected with HIV. Section 1 | Treatment, Care | PLHIV | Direct | Adopted | Facilitates |
| Italy |  | A6 | Guidelines on the use of Antiretroviral Therapy and the Diagnostic-Clinical Management of People Infected with HIV. Section 4 | Treatment | Children living with HIV | Direct | Adopted | Facilitates |
| Italy |  | A9 | Guidelines on the use of Antiretroviral Therapy and the Diagnostic-Clinical Management of People Infected with HIV. Section 3 | Care, Testing | People living with HIV, People newly diagnosed with TB | Direct | Adopted | Facilitates |
| Italy |  | A10 | Guidelines on the use of Antiretroviral Therapy and the Diagnostic-Clinical Management of People Infected with HIV | Testing, Treatment, Prevention | People living with HIV, Partners of PLHIV | Direct | Adopted | Facilitates |
| Italy |  | A11 | Guidelines on the use of Antiretroviral Therapy and the Diagnostic-Clinical Management of People Infected with HIV. Section 4 | Testing, Treatment, Prevention | WLHIV, newborns | Direct | Adopted | Facilitates |
| Italy |  | A12 | Guidelines on the use of Antiretroviral Therapy and the Diagnostic-Clinical Management of People Infected with HIV. Section 2 | Treatment | PLHIV | Direct | Adopted | Facilitates |
| Italy |  | B6.2 | Guidelines on the use of Antiretroviral Therapy and the Diagnostic-Clinical Management of People Infected with HIV | Prevention | Gay men and other MSM, Transgender people, People who inject drugs, Heterosexuals with (Inconsistent use of condoms and casual relationships or with partners a risk for HIV and at least one of the following conditions, at least one anal intercourse without the use of a condom, Treatment of an STI, Previous PEP) | Direct | Adopted | Facilitates |
| Italy | Law 135 of 5 June 1990: Program of urgent interventions for the prevention and fight against AIDS. (*Programma di interventi urgenti per la prevenzione e la lotta contro l'AIDS*) https://www.gazzettaufficiale.it/eli/id/1990/06/08/090G0179/sg | B4 | Program of urgent interventions for the prevention and fight against AIDS. Article 5, Paragraph 3 | Testing | General population | Direct | Adopted | Facilitates |
| Italy | Law 20 February 1958, N°75 Abolition of the regulation of prostitution and fight against the exploitation of the prostitution of others. (L*egge 20 febbraio 1958, n. 75 Abolizione della regolamentazione della prostituzione e lotta contro lo sfruttamento della prostituzione altrui*) ttps://www.normattiva.it/atto/caricaDettaglioAtto?atto.dataPubblicazioneGazzetta=1958-03-04&atto.codiceRedazionale=058U0075&atto.articolo.numero=0&atto.articolo.sottoArticolo=1&atto.articolo.sottoArticolo1=10&qId=101df4b4-6c38-4a92-b943-d9600ece4b93&tabID=0.38256514825653176&title=lbl.dettaglioAtto | C2 | Abolition of the regulation of prostitution and fight against the exploitation of the prostitution of others | Prevention, Testing, Treatment, Care | Sex workers | Indirect | Partially adopted | Hinders |
| Italy | Law 20 May 2016, n. 76 Regulation of civil unions between persons of the same sex and regulation of cohabitation (*Legge 20 maggio 2016, n. 76 Regolamentazione delle unioni civili tra persone dello stesso sesso e disciplina delle convivenze*) https://www.normattiva.it/uri-res/N2Ls?urn:nir:stato:legge:2016-05-20;76 | C16 | Regulation of civil unions between persons of the same sex and regulation of cohabitation | Prevention, Testing, Treatment, Care | Gay men and other MSM, Transgender people, Lesbians | Indirect | Adopted | Facilitates |
| Italy | Law 27 December 2006, n. 296 "Provisions for the formation of the annual and multi-year state budget (financial law 2007)" (*Legge 27 dicembre 2006, n. 296 "Disposizioni per la formazione del bilancio annuale e pluriennale dello Stato (legge finanziaria 2007*)") https://www.parlamento.it/parlam/leggi/06296l.htm | C12.2 | Financial Law. Article 1 | Prevention | General population | Indirect | Adopted | Facilitates |
| Italy | Law 6 March 1998, n. 40. "Regulation of immigration and rules on the status of foreigners." (*Legge 6 marzo 1998, n. 40. "Disciplina dell'immigrazione e norme sulla condizione dello straniero.")* https://www.parlamento.it/parlam/leggi/98040l.htm | A7 | Regulation of immigration and rules on the status of foreigners. Chapter I of Title V, Articles 32 and 33 | Prevention, Testing, Treatment, Care | Migrants | Indirect | Partially adopted | Facilitates |
| Italy | Law n. 164 of April 14, 1982: "Rules Concerning the Rectification of Sex-Attribution" (*Norme in materia di rettificazione di attribuzione di sesso*) https://www.normattiva.it/uri-res/N2Ls?urn:nir:stato:legge:1982-04-14;164 | C9 | Rules Concerning the Rectification of Sex-Attribution. Article 454 ammended | Prevention, Testing, Treatment, Care | Transgender people | Indirect | Adopted | Facilitates |
| Italy | Law No. 38/2009 on Urgent Measures regarding Sexual Violence https://evaw-global-database.unwomen.org/es/countries/europe/italy/2009/law-no-382009-on-urgent-measures-regarding-sexual-violence | C13 | Law on Urgent Measures regarding Sexual Violence. Article 612bis modified | Prevention, Testing, Treatment, Care | General population | Indirect | Adopted | Facilitates |
| Italy | Legislative Decree 3 July 2017, n. 117 of the Third Sector Code, pursuant to article 1, paragraph 2, letter b), of the law of 6 June 2016, n. 106. (17G00128) (*Decreto Legislativo 3 luglio 2017, n. 117 Codice del Terzo settore, a norma dell'articolo 1, comma 2, lettera b), della legge 6 giugno 2016, n. 106. (17G00128)*) https://www.gazzettaufficiale.it/dettaglio/codici/terzoSettore | C14.2 | Code of the Third Sector. Articles 21, 23 and 25 | Prevention, Testing, Treatment, Care | General population | Indirect | Adopted | Facilitates |
| Italy | National Action Plan on Dependences 2022-2025 (*Piano di Azione Nazionale Dipendenze 2022-2025) https://www.cnca.it/wp-content/uploads/2022/10/PAND.pdf* | B7 | National Action Plan on Dependencies | Prevention | People who inject drugs, Incarcerated people | Direct | Adopted | Facilitates |
| Italy |  | C4 | National Action Plan on Dependencies | Prevention, Testing, Care | People who inject drugs, All drug users | Direct | Partially adopted | Facilitates |
| Italy | National Plan of Interventions Against HIV and AIDS (*Piano Nazionale Di Interventi Contro HIV e AIDS (PNAIDS)*) https://www.salute.gov.it/imgs/C_17_pubblicazioni_2655_allegato.pdf | A2 | National Plan of Interventions Against HIV and AIDS. Third Section | Treatment, Prevention | People living with HIV, Partners of PLHIV | Direct | Adopted | Facilitates |
| Italy |  | B2 | National Plan of Interventions Against HIV and AIDS. Second Section | Testing | General population | Direct | Adopted | Facilitates |
| Italy |  | B9 | National Plan of Interventions Against HIV and AIDS. Subsection 2.4 | Prevention, Testing, Treatment | Sex workers | Direct | Adopted | Facilitates |
| Italy |  | B10 | National Plan of Interventions Against HIV and AIDS. Subsection 2.1 | Prevention, Treatment, Testing | Gay men and other MSM | Direct | Adopted | Facilitates |
| Italy |  | B11 | National Plan of Interventions Against HIV and AIDS. Subsection 2.3 | Prevention, Testing | Incarcerated people | Direct | Adopted | Facilitates |
| Italy |  | D1 | National Plan of Interventions Against HIV and AIDS | Prevention, Testing, Treatment, Care | Gay men and other MSM, Sex workers, Transgender people, People who inject drugs, Incarcerated people, Migrants, People living with HIV, Partners of people living with HIV, General population | Direct | Partially adopted | Facilitates |
| Italy |  | D2 | National Plan of Interventions Against HIV and AIDS | Prevention, Testing, Treatment, Care | Gay men and other MSM, Sex workers, Transgender people, People who inject drugs, Incarcerated people, Gay men and other MSM, Sex workers, Transgender people, People who inject drugs, Incarcerated people, Migrants, People living with HIV, Partners of people living with HIV, General population | Direct | Partially adopted | Facilitates |
| Italy | National Prevention Plan (*Piano Nazionale della Prevenzione 2020-2025*) https://www.salute.gov.it/imgs/C_17_notizie_5029_0_file.pdf | B13 | National Prevention Plan. Macro objective 6 | Prevention, Testing | General population, young people | Direct | Adopted | Facilitates |
| Italy | Penal Code (Codice Penale) https://www.gazzettaufficiale.it/sommario/codici/codicePenale | C1 | Penal Code does not include any provisions against same-sex relations. | Prevention, Testing, Treatment, Care | Gay men and other MSM | Indirect | Adopted | Facilitates |
| Italy |  | C7 | Penal Code. Article 582 | Prevention, Testing, Treatment, Care | PLHIV | Indirect | Partially adopted | Hinders |
| Italy |  | C8-E | Penal Code. Articles 604-bis and 605-ter | Prevention, Testing, Treatment, Care | Migrants | Indirect | Adopted | Facilitates |
| Italy | Resolution February 8, 2023 National Health Fund 2022. Allocation of financial resources for the National Health Service (Resolution n. 4/2023) (*Delibera 8 febbraio 2023 Fondo sanitario nazionale 2022. Riparto delle disponibilita' finanziarie per il Servizio sanitario nazionale (Delibera n. 4/2023)*) https://www.gazzettaufficiale.it/eli/id/2023/03/21/23A01710/sg | D4 | National Health Fund. Resolution n. 4/2023 | Prevention, Testing, Treatment, Care | General population | Direct | Adopted | Facilitates |
| Italy | The Principles of the National Health Service (*I Principi del Servizio Sanitario Nazionale (SSN)*) https://www.salute.gov.it/portale/lea/dettaglioContenutiLea.jsp?area=Lea&id=5073&lingua=italiano&menu=vuoto | D5 | Principles of the National Health Service | Prevention, Testing, Treatment, Care | General population | Indirect | Partially adopted | Facilitates |
| Italy (Lombardy) | Diagnostic Therapeutic Care Path of the Patient Suffering from HIV/AIDS Infection/Disease (*Percorso Diagnostico Terapeutico Assistenziale (PDTA) del Paziente Afetto da Infezione/Malattia HIV/AIDS*) https://www.simit.org/images/regioni/lombardia/comunicazioni/Documenti%20Regionali/PDTA%20HIV%20Regione%20Lombardia%202021.pdf | A1 | Regional clinical HIV guideline | Treatment, Prevention | PLHIV | Direct | Adopted | Facilitates |
| Italy (Lombardy) |  | A3 | Regional clinical HIV guideline | Treatment, Prevention, Care | People living with HIV, Partners of PLHIV | Direct | Adopted | Facilitates |
| Italy (Lombardy) |  | A10 | Regional clinical HIV guideline | Treatment, Prevention | People living with HIV, Partners of PLHIV | Direct | Adopted | Facilitates |
| Italy (Lombardy) | Regional Preventions Plan 2021-2025 (*Piano Regionale Prevenzione 2021-2025)* https://www.regione.lombardia.it/wps/wcm/connect/f6af4f98-18f9-4441-8550-20483ba95211/PRP_2021_2025_Lombardia.pdf?MOD=AJPERES&CACHEID=ROOTWORKSPACE-f6af4f98-18f9-4441-8550-20483ba95211-oj8T2oQ | B13 | Regional Prevention Plan. Free program PL21 | Prevention | General population | Direct | Adopted | Facilitates |
| Latvia | About the conceptual report "On Latvia's accession to the Council of Europe Convention on preventing and combating violence against women and domestic violence" (*Par konceptuālu ziņojumu "Par Latvijas pievienošanos Eiropas Padomes Konvencijai par vardarbības pret sievietēm un vardarbības ģimenē novēršanu un apkarošanu"*) https://likumi.lv/ta/id/282156 | C13 | Convention on preventing and combating violence against women and domestic violence | Prevention, Testing, Treatment, Care | Sex workers, Transgender people, Women in general and specifically victims of violence. | Indirect | Adopted | Facilitates |
| Latvia | Amendments to the regulations of the Cabinet of Ministers of September 15, 2008 no. 746 "Procedures for creating, supplementing and maintaining the register of patients suffering from certain diseases" (*Grozījumi Ministru kabineta 2008. gada 15. septembra noteikumos Nr. 746 "Ar noteiktām slimībām slimojošu pacientu reģistra izveides, papildināšanas un uzturēšanas kārtība"*) https://likumi.lv/ta/id/304158 | D8 | Regulations for creating, supplementing and maintaining the register of patients suffering from certain diseases | Prevention, Treatment | PLHIV | Direct | Not adopted | Hinders |
| Latvia | Civil law. First Part. Family law (Civillikums. PIRMĀ DAĻA. Ģimenes tiesības) https://likumi.lv/ta/id/90223 | C16 | Civil Law. Article 35 | Prevention, Testing, Treatment, Care | Gay men and other MSM, Transgender people, Lesbians | Indirect | Not adopted | Hinders |
| Latvia | Clinical guidelines for the diagnosis, treatment, and prevention of human immunodeficiency virus (HIV) infection (*Cilvēka imūndeficīta vīrusa (HIV) infekcijas diagnostikas, ārstēšanas un profilakses klīniskās vadlīnijas*) https://www.vmnvd.gov.lv/lv/media/1469/download?attachment | A6 | Clinical HIV guidelines | Prevention, Testing, Treatment, Care | PLHIV | Direct | Adopted | Facilitates |
| Latvia |  | A10.2 | Clinical HIV guidelines | Prevention, Testing, Treatment, Care | PLHIV | Direct | Adopted | Facilitates |
| Latvia | Constitution of the Republic of Latvia (*Latvijas Republikas Satversme*) https://likumi.lv/ta/id/57980 | C8 | The Constitution. Articles 91 and 111 | Prevention, Testing, Treatment, Care | General population | Indirect | Adopted | Facilitates |
| Latvia |  | C11 | The Constitution. Chapter VIII, Article 111 | Prevention, Testing, Treatment, Care | General population | Indirect | Adopted | Facilitates |
| Latvia | Criminal Code (Kriminālkodekss) https://www.warnathgroup.com/wp-content/uploads/2015/03/Latvia-Criminal-Code.pdf | C1 | Criminal Code. Article 124.1 repealed | Prevention, Testing, Treatment, Care | Gay men and other MSM | Indirect | Adopted | Facilitates |
| Latvia | Criminal law (*Krimināllikums*) https://likumi.lv/ta/id/88966 | C7 | Criminal Law. Article 134 | Prevention, Testing, Treatment, Care | PLHIV | Indirect | Not adopted | Hinders |
| Latvia | Education Act (*Izglītības likums*) https://likumi.lv/ta/id/50759-izglitibas-likums | C12 | Education Act. Article 8 | Prevention | Children and youth | Indirect | Adopted | Facilitates |
| Latvia | Health promotion and disease prevention plan (*Veselības veicināšanas un slimību profilakses plāns)* https://esparveselibu.lv/sites/default/files/inline-files/Ves_veic_plans_aktual_161220_gala.pdf | B13 | Health promotion and disease prevention plan | Prevention | Ethnic minorities with integration problems (including refugees), People who inject drugs, | Indirect | Adopted | Facilitates |
| Latvia | HIV tests https://www.spkc.gov.lv/lv/hiv-tests | A2 | HIV tests services | Testing, Treatment, Prevention | General population | Direct | Not adopted | Hinders |
| Latvia | HIV/AIDS Statistics (*HIV/AIDS statistika*) https://www.spkc.gov.lv/lv/hivaids-statistika | D9 | HIV/AIDS Statistics | Prevention, Treatment, Care | PLHIV | Direct | Partially adopted | Facilitates |
| Latvia | Law of the Ombudsman (*Tiesībsarga likums*) https://likumi.lv/ta/id/133535-tiesibsarga-likums | C10 | Law of the Ombudsman | Treatment, Care | General population | Indirect | Adopted | Facilitates |
| Latvia | Law on the Legal Circulation of Narcotic and Psychotropic Substances and Medicines, as well as Precursors. https://likumi.lv/ta/id/40283-narkotisko-un-psihotropo-vielu-un-zalu-ka-ari-prekursoru-likumigas-aprites-likums | C3 | Law on the Legal Circulation of Narcotic and Psychotropic Substances and Medicines. Article 48 | Prevention, Testing, Treatment, Care | People who inject drugs, People who use drugs in general | Indirect | Not adopted | Hinders |
| Latvia | Order of the Cabinet of Ministers No. 630 On the action plan for limiting the spread of HIV infection, sexually transmitted infections, B and C hepatitis for the years 2018-2020. (*Par HIV infekcijas, seksuālās transmisijas infekciju, B un C hepatīta izplatības ierobežošanas rīcības plānu 2018.-2020. gadam*) https://likumi.lv/ta/id/294753 | D1 | National HIV/AIDS plan | Prevention, Testing, Treatment, Care | Gay men and other MSM, Sex workers, People who inject drugs, Incarcerated people | Direct | Partially adopted | Facilitates |
| Latvia |  | D2 | National HIV/AIDS plan. Section III | Prevention, Testing, Treatment, Care | Gay men and other MSM, Sex workers, People who inject drugs, PLHIV | Direct | Partially adopted | Facilitates |
| Latvia | Patent Law (*Patentu likums)* https://likumi.lv/ta/id/153574-patentu-likums | D7 | Patent Law. Article 54 | Treatment | PLHIV | Indirect | Adopted | Facilitates |
| Latvia | Plan for the improvement of preventive measures and health care services in the field of reducing the prevalence of alcohol and drug abuse for the years 2023-2025 (*Profilakses pasākumu un veselības aprūpes pakalpojumu uzlabošanas plāns alkoholisko dzērienu un narkotisko vielu lietošanas izplatības mazināšanas jomā 2023.–2025. gadam*) https://likumi.lv/ta/id/336736 | C4 | Alcohol and drug abuse plan | Prevention | People who inject drugs, Incarcerated people, People who use drugs in general | Direct | Adopted | Facilitates |
| Latvia |  | C5 | Alcohol and drug abuse plan | Prevention | People who inject drugs, Incarcerated people | Direct | Adopted | Facilitates |
| Latvia | Procedure for implementation of health care for arrested and convicted persons (*Apcietināto un notiesāto personu veselības aprūpes īstenošanas kārtība*) https://likumi.lv/ta/id/274511 | A8 | Procedure for implementation of health care for arrested and convicted persons | Testing, Treatment, Care | Incarcerated people | Indirect | Adopted | Facilitates |
| Latvia | Procedure for organization and payment of health care services (*Veselības aprūpes pakalpojumu organizēšanas un samaksas kārtība)* https://likumi.lv/ta/id/301399-veselibas-aprupes-pakalpojumu-organizesanas-un-samaksas-kartiba | D6 | Procedure for organization and payment of health care services. Section 3.5, Disposition 56.2.11 | Testing, Treatment, Care | PLHIV and their sexual partners | Direct | Adopted | Facilitates |
| Latvia | Rational Pharmacotherapy Recommendations for Antiretroviral Therapy Paid from State Budget Funds for the Treatment of HIV/AIDS Infection (*Racionālas Farmakoterapijas Rekomendācijas No Valsts Budžeta Līdzekļiem Apmaksātai Antiretrovirālai Terapijai Hiv/Aids Infekcijas Ārstēšanai*) https://www.vmnvd.gov.lv/lv/media/1250/download | A1 | HIV treatment guidelines | Treatment, Prevention | PLHIV | Direct | Adopted | Facilitates |
| Latvia |  | A3 | HIV treatment guidelines | Prevention, Treatment | PLHIV, pregnant women and children | Direct | Adopted | Facilitates |
| Latvia |  | A5 | HIV treatment guidelines. Section 6 | Treatment | PLHIV, pregnant women and children | Direct | Adopted | Facilitates |
| Latvia |  | A9 | HIV treatment guidelines. Section 8.5 | Treatment | PLHIV, pregnant women and children | Direct | Adopted | Facilitates |
| Latvia |  | A10.1 | HIV treatment guidelines | Treatment | PLHIV, pregnant women and children | Direct | Adopted | Facilitates |
| Latvia |  | A11 | HIV treatment guidelines. Section 8 | Prevention, Treatment | PLHIV | Direct | Adopted | Facilitates |
| Latvia |  | D5 | HIV treatment guidelines | Treatment |  | Direct | Partially adopted | Facilitates |
| Latvia | Recommendations for express testing, pre-test and post-test counseling at HIV prevention points (*Rekomendācijas eksprestestēšanā, pirmstesta un pēctesta konsultēšanā HIV profilakses punktos*) https://www.spkc.gov.lv/lv/media/18303/download?attachment | B1 | Recommendations for HIV testing and counselling | Testing | Gay men and other MSM, Transgender people, People who inject drugs | Direct | Adopted | Facilitates |
| Latvia | Regulations of the Cabinet of Ministers No. 32 Prostitution Restriction Regulations (Prostitūcijas ierobežošanas noteikumi) https://likumi.lv/ta/id/169772 | C2 | Prostitution Restriction Regulations | Prevention, Testing, Treatment, Care | Sex workers | Indirect | Partially adopted | Hinders |
| Latvia | Regulations of the Cabinet of Ministers of June 14, 2005 No. 413 "Procedure for mandatory medical and laboratory examination of persons, mandatory and forced isolation and treatment in cases of infectious diseases". https://likumi.lv/ta/id/110743 | B4 | Procedure for mandatory medical and laboratory examination of persons | Testing | General population | Direct | Not adopted | Facilitates |
| Latvia | Regulations of the Cabinet of Ministers of September 19, 2006 No. 744 "Procedures for identification of contact persons, primary medical examination, laboratory examination and medical observation". (*Ministru kabineta noteikumi Nr.774 Rīgā 2006.gada 19.septembrī (prot. Nr.48 20.§) Kontaktpersonu noteikšanas, primārās medicīniskās pārbaudes, laboratoriskās pārbaudes un medicīniskās novērošanas kārtība*) https://likumi.lv/ta/id/144279 | B3 | Procedures for identification of contact persons, primary medical examination, laboratory examination and medical observation | Testing | PLHIV and their sexual partners | Direct | Partially adopted | Facilitates |
| Latvia | Sexual and Reproductive Health Law (*Seksuālās un reproduktīvās veselības likums*) https://likumi.lv/ta/id/58982 | B12 | Sexual and Reproductive Health Law. Chapter II to VI | Prevention, Testing, Treatment, Care | General population | Direct | Adopted | Facilitates |
| Latvia | Suport person services (*Atbalsta personas pakalpojumi*) https://www.spkc.gov.lv/lv/atbalsta-personas-pakalpojumi | A12 | Suport person services | Care | People who received a positive test result for HIV, HBV, or HCV. | Direct | Adopted | Facilitates |
| Latvia | Support Measures for harm reduction (*Atbalsta pasākumi kaitējuma mazināšanai*) https://www.spkc.gov.lv/lv/atbalsta-pasakumi-kaitejuma-mazinasanai | B7 | Support Measures for harm reduction | Prevention | People who inject drugs, Sex workers, Gay men and other MSM, Exconvicts, Anyone "of interest" who has been at risk of contracting HIV, HCV, HBV, or syphilis. | Direct | Adopted | Facilitates |
| Latvia | Treatment procedures for patients addicted to alcohol, narcotics, psychotropic substances, toxic substances, gambling or computer games (*Alkohola, narkotisko, psihotropo, toksisko vielu, azartspēļu vai datorspēļu atkarības slimnieku ārstēšanas kārtība*) https://likumi.lv/ta/id/243233 | C6 | Treatment procedures for patients with addictions | Prevention | People who inject drugs | Direct | Adopted | Facilitates |
| Netherlands | Agreement on prescription regulation (*Akkoord afspraken prescriptieregeling*) https://www.hivvereniging.nl/downloads/verenigingsblog/387-hiv-vereniging-levering-hiv-medicatie-per-drie-maanden-2022/file | A4 | Agreement on prescription regulation. Article 3 | Treatment, Care | PLHIV | Indirect | Adopted | Facilitates |
| Netherlands | Civil Code (*Burgerlijk Wetboek*) https://www.ilo.org/dyn/natlex/natlex4.detail?p_lang=en&p_isn=91671 | C9 | Civil Code. Article 1:28 | Prevention, Testing, Treatment, Care | Transgender people | Indirect | Adopted | Facilitates |
| Netherlands | Civil Code Book 2 (*Burgerlijk Wetboek Boek 2*) https://wetten.overheid.nl/BWBR0003045/2023-05-01 | C14 | Civil Code. Book 2, Article 3 | Prevention, Testing, Treatment, Care | General population | Indirect | Adopted | Facilitates |
| Netherlands | Criminal Code (*Wetboek van Strafrecht*) https://www.hivjustice.net/country/nl/ | C7 | Criminal Code. Section 302 | Prevention, Testing, Treatment, Care | PLHIV | Indirect | Partially adopted | Hinders |
| Netherlands | Dutch multidisciplinary guideline Pre-exposure prophylaxis (PrEP) for the prevention of HIV (*Nederlandse multidisciplinaire richtlijn Pre-expositie profylaxe (PrEP) ter preventie van hiv*) https://www.soaaids.nl/files/2022-07/20220711-PrEP-richtlijn-Nederland-versie-3-update-2022.pdf | B6 | PrEP Guidelines. Section 1 | Prevention | Gay men and other MSM, Transgender people, Partners of MSM, transgender people, People from an area with a high HIV prevalence. | Direct | Adopted | Facilitates |
| Netherlands | Health Insurance Act (*Zorgverzekeringswet*) https://wetten.overheid.nl/BWBR0018450/2023-01-01 | D5 | Health Insurance Act. Chapters 2 and 3 | Prevention, Testing, Treatment, Care | General population | Direct | Partially adopted | Facilitates / Hinders |
| Netherlands |  | D6 | Health Insurance Act. Article 123 | Prevention, Testing, Treatment, Care | General population | Indirect | Not adopted | Hinders |
| Netherlands | HIV infection guideline (*Hivinfectie Richtlijn*) https://lci.rivm.nl/richtlijnen/hivinfectie | B1 | Guidelines on HIV infection. Section "Diagnostics" | Testing | Gay men and other MSM, People who inject drugs, General population | Direct | Adopted | Facilitates |
| Netherlands |  | B2 | Guidelines on HIV infection | Testing | General population | Direct | Adopted | Hinders |
| Netherlands |  | B4 | Guidelines on HIV infection. Section "Testing for HIV inside and outside the hospital" | Testing | General population | Direct | Adopted | Facilitates |
| Netherlands | HIV Monitoring Report (*HIV Monitoring Rapport*) https://www.hiv-monitoring.nl/application/files/1816/6851/5357/NL_HIV_MONITORING_REPORT_2022.pdf | D9 | HIV Monitoring Report |  | PLHIV | Direct | Partially adopted | Facilitates |
| Netherlands | HIV Treatment Guidelines (*Richtlijnen HIV-Behandeling*) http://richtlijnhiv.nvhb.nl/index.php/ | A1 | HIV Treatment Guidelines. Section 2.1 | Prevention, Treatment | PLHIV and their sexual partners | Direct | Adopted | Facilitates |
| Netherlands |  | A2 | HIV Treatment Guidelines. Section 2.2 | Prevention, Treatment | PLHIV and their sexual partners | Direct | Adopted | Facilitates |
| Netherlands |  | A3 | HIV Treatment Guidelines. Section 2.1 | Prevention, Treatment, Care | PLHIV | Direct | Adopted | Facilitates |
| Netherlands |  | A5 | HIV Treatment Guidelines. Section 4.1 | Prevention, Treatment | PLHIV | Direct | Adopted | Facilitates |
| Netherlands |  | A6 | HIV Treatment Guidelines. Chapter 3 | Treatment | Children living with HIV | Direct | Adopted | Facilitates |
| Netherlands |  | A9 | HIV Treatment Guidelines. Chapter 11 | Care | PLHIV | Direct | Adopted | Facilitates |
| Netherlands |  | A10 | HIV Treatment Guidelines. Chapter 2 | Prevention, Treatment | PLHIV and their sexual partners | Direct | Adopted | Facilitates |
| Netherlands |  | A11 | HIV Treatment Guidelines. Chapters 3 and 7 | Prevention, Treatment | Women living with HIV, Newborns exposed to/infected with HIV | Direct | Adopted | Facilitates |
| Netherlands | Hygiene guideline for correctional institutions and national services (*Hygiënerichtlijn voor justitiële inrichtingen en landelijke diensten*) https://www.rivm.nl/hygienerichtlijnen/justitiele-inrichtingen | B11 | Hygiene guideline for correctional institutions and national services. Section 5.3 | Prevention | Incarcerated people | Direct | Partially adopted | Facilitates |
| Netherlands | Law College for Human Rights (*Wet College voor de rechten van de mens*) https://wetten.overheid.nl/BWBR0030733/2013-01-01 | C10 | Law College for Human Rights. Article 1.3 | Treatment, Care | General population | Indirect | Adopted | Facilitates |
| Netherlands | Linkage Act (*Koppelingswet*) https://www.eerstekamer.nl/wetsvoorstel/24233_koppelingswet | A7 | Linkage Act | Prevention, Testing, Treatment, Care | Migrants | Indirect | Partially adopted | Hinders |
| Netherlands | Marriage Opening Act (*Wet openstelling huwelijk*) https://wetten.overheid.nl/BWBR0012099/2001-04-01 | C16 | Marriage Opening Act | Prevention, Testing, Treatment, Care | Gay men and other MSM, Transgender people, Lesbians | Indirect | Adopted | Facilitates |
| Netherlands | Medical Treatment Agreement Act (*Wet geneeskundige behandelingsovereenkomst*) https://wetten.overheid.nl/BWBR0005290/2023-02-18/#Boek7_Titeldeel7_Afdeling5_Artikel448 | B5 | Medical Treatment Agreement Act. Article 7:450.1 and Article 7:451.2 | Testing, Treatment | Minors aged 12 to 16 years old | Indirect | Not adopted | Hinders |
| Netherlands | National Action Plan for STI, HIV and sexual health (*Nationaal Actieplan soa, hiv en seksuele gezondheid*) https://www.rivm.nl/bibliotheek/rapporten/2018-0034.pdf | D1 | National Action Plan for STI, HIV and sexual health | Prevention, Testing, Treatment, Care | General population, PLHIV | Direct | Partially adopted | Facilitates |
| Netherlands |  | D2 | National Action Plan for STI, HIV and sexual health. Sections 4.2 and 4.3 |  | PLHIV | Direct | Partially adopted | Facilitates |
| Netherlands | National Budget (*Begroting*) https://open.overheid.nl/documenten/ronl-e2c9f907e7c2a5c561901289e3e3bb5769cab44a/pdf | D4 | National budget does not include HIV | Prevention, Testing, Treatment, Care | General population | N/A | Not adopted | Hinders |
| Netherlands | National Prevention Agreement (*Nationaal Preventieakkoord*) https://open.overheid.nl/documenten/ronl-1f7b7558-4628-477d-8542-9508d913ab2c/pdf | B13 | National Prevention Agreement does not include HIV |  | General population (smokers, obese, alcohol drinkers) | Indirect | Partially adopted | N/A |
| Netherlands | Official Gazette 2012, 470 (Staatsblad 2021, 470) https://zoek.officielebekendmakingen.nl/stb-2012-470.html | B8 | Decree on the core objectives of education in the field of sexuality and sexual diversity. | Prevention | Students | Indirect | Adopted | Facilitates |
| Netherlands | Opium Act (*Opiumwet*) https://wetten.overheid.nl/BWBR0001941/2023-01-01 | C3 | Opium Act. Articles 2 and 3 | Prevention, Testing, Treatment, Care | People who inject drugs | Indirect | Not adopted | Facilitates / Hinders |
| Netherlands | Penal code (*Wetboek van Strafrecht*) https://www.ilo.org/dyn/natlex/natlex4.detail?p_lang=en&p_isn=69274&p_country=NLD&p_count=1787&p_classification=01.04&p_classcount=12 | C1 | The Penal Code does not explicitly criminalize homosexuality | Prevention, Testing, Treatment, Care | Gay men and other MSM | Indirect | Adopted | Facilitates |
| Netherlands |  | C2 | The Penal Code. Article 250bis | Prevention, Testing, Treatment, Care | Sex workers | Indirect | Adopted | Facilitates |
| Netherlands | State Patent Act (*Rijksoctrooiwet*) https://wetten.overheid.nl/BWBR0007118/2021-08-01 | D7 | State Patent Act | Treatment |  | Indirect | Adopted | Facilitates |
| Netherlands | STI and HIV partner management (*Soa en hiv partnermanagement*) https://lci.rivm.nl/sites/default/files/2018-12/Draaiboek%20Soa%20en%20hiv%20partnermanagement%20%28update%202018%29.pdf | B3 | STI and HIV partner management. Section 4.2 and Article 457.1 | Prevention, Testing | PLHIV and their sexual partners | Direct | Adopted | Facilitates |
| Netherlands | Supplementary Provisions for the Processing of Personal Data in Healthcare (*Wet aanvullende bepalingen verwerking persoonsgegevens in de zorg*) https://wetten.overheid.nl/BWBR0023864/2020-07-01 | D8 | Supplementary Provisions for the Processing of Personal Data in Healthcare. Article 17a | Testing, Treatment | General population | Indirect | Adopted | Facilitates |
| Netherlands | Temporary Restraining Order Act (*Wet tijdelijk huisverbod*) https://wetten.overheid.nl/BWBR0024649/2020-07-01 | C13 | Temporary Restraining Order Act. Articles 2.1 and 11.2 | Prevention, Testing, Treatment, Care | General population | Indirect | Adopted | Facilitates |
| Netherlands | The Constitution of the Kingdom of the Netherlands (*Grondwet voor het Koninkrijk der Nederlanden*) https://www.government.nl/documents/reports/2019/02/28/the-constitution-of-the-kingdom-of-the-netherlands | C8 | The Constitution. Article 1 | Prevention, Testing, Treatment, Care | General population | Indirect | Adopted | Facilitates |
| Netherlands |  | C8-A | The Constitution. Article 1 | Prevention, Testing, Treatment, Care | PLHIV | Indirect | Adopted | Facilitates |
| Netherlands |  | C8-B | The Constitution. Article 1 | Prevention, Testing, Treatment, Care | Gay men and other MSM | Indirect | Adopted | Facilitates |
| Netherlands |  | C8-C | The Constitution. Article 1 | Prevention, Testing, Treatment, Care | People who inject drugs | Indirect | Adopted | Facilitates |
| Netherlands |  | C8-D | The Constitution. Article 1 | Prevention, Testing, Treatment, Care | Transgender people | Indirect | Adopted | Facilitates |
| Netherlands |  | C8-E | The Constitution. Article 1 | Prevention, Testing, Treatment, Care | Migrants | Indirect | Adopted | Facilitates |
| Netherlands |  | C8-F | The Constitution. Article 1 | Prevention, Testing, Treatment, Care | Incarcerated people, General population | Indirect | Adopted | Facilitates |
| Poland | Law on the Penitentiary Service (*Ustawa o Służbie Więziennej*) https://isap.sejm.gov.pl/isap.nsf/download.xsp/WDU20100790523/U/D20100523Lj.pdf | A8.2 | Law on the Penitentiary Service. Article 2 | Treatment, Prevention, Care, Testing | Incarcerated people | Indirect | Adopted | Facilitates |
| Poland | Art 189 Civil Procedural Code (*Art 189 Kodeks Postępowania Cywilnego*) https://lexlege.pl/kpc/art-189/ | C9 | Civil Procedural Code. Article 189 | Prevention, Testing, Treatment, Care | General population | Indirect | Adopted | Facilitates |
| Poland | Art 68 Constitution of the Republic of Poland (*Art 68 Konstytucja Rzeczpospolitej Polskiej*) https://lexlege.pl/konstytucja-rzeczypospolitej-polskiej/art-68/ | C11 | The Constitution. Article 68 | Prevention, Testing, Treatment, Care | General population | Indirect | Adopted | Facilitates |
| Poland | Constitution of the Republic of Poland (*Konstytucja Rzeczpospolitej Polskiej*) https://lexlege.pl/konstytucja-rzeczypospolitej-polskiej/art-32/ | C8.1 | The Constitution. Article 32 | Prevention, Testing, Treatment, Care | General population | Indirect | Adopted | Facilitates |
| Poland | Health Services Office, Penitentiary Service (*Biuro Służby Zdrowia, Służba Więzienna*) https://www.sw.gov.pl/jednostka/biuro-sluzby-zdrowia | A8.1 | Health Services Office, Penitentiary Service | Treatment, Prevention, Care, Testing | Incarcerated people | Indirect | Adopted | Facilitates |
| Poland | HIV infection and AIDS cases in Poland in 2022 (*Zakażenia HIV i zachorowania na AIDS w Polsce w 2022 roku*) https://wwwold.pzh.gov.pl/oldpage/epimeld/hiv_aids/index.htm | D9 | Epidemiology of infectious diseases and surveillance | Testing | General population | Direct | Adopted | Facilitates |
| Poland | Law dated 15 July 1987 on the Citizens Rights Ombudsman (*Ustawa z dnia 15 lipca 1987 r. o Rzeczniku Praw Obywatelskich*) https://isap.sejm.gov.pl/isap.nsf/DocDetails.xsp?id=WDU19870210123 | C10 | Citizens Rights Ombudsman | Treatment, Care | General population | Indirect | Adopted | Facilitates |
| Poland | Law dated 27 August 2004 on health services financed from public funds. (*Ustawa z dnia 27 sierpnia 2004 r. o świadczeniach opieki zdrowotnej finansowanych ze środków publicznych*) https://isap.sejm.gov.pl/isap.nsf/download.xsp/WDU20042102135/U/D20042135Lj.pdf | D6.2 | Health services financed from public funds law. Article 15 | Prevention, Testing, Treatment, Care | All PLHIV | Indirect | Adopted | Facilitates |
| Poland | Law dated 29 July 2005 on Combating domestic violence (Ustawa z dnia 29 lipca 2005 r. o przeciwdziałaniu przemocy w rodzinie) https://lexlege.pl/ustawa-o-przeciwdzialaniu-przemocy-w-rodzinie/ | C13.1 | Law on Combating domestic violence | Prevention, Testing, Treatment, Care | General population | Indirect | Adopted | Facilitates |
| Poland | Law dated 29 July 2005 on Combating drug addiction (Ustawa z dnia 29 lipca 2005 o przeciwdziałaniu narkomanii) https://lexlege.pl/ustawa-o-przeciwdzialaniu-narkomanii/art-62/ | C3 | Law on Combating drug addiction | Prevention, Testing, Treatment, Care | People who inject drugs | Indirect | Not adopted | Hinders |
| Poland | Law dated 3 December 2010 on implementation of certain European Union provisions regarding equal treatment (*Ustawa z dnia 3 grudnia 2010 r. o wdrożeniu niektórych przepisów Unii Europejskiej w zakresie równego traktowania*) https://isap.sejm.gov.pl/isap.nsf/download.xsp/WDU20102541700/U/D20101700Lj.pdf | C8.2 | Equal treatment law. | Prevention, Testing, Treatment, Care | Gay men and other MSM, General population | Indirect | Adopted | Facilitates |
| Poland |  | C8-B | Equal treatment law. | Prevention, Testing, Treatment, Care | Gay men and other MSM | Indirect | Adopted | Facilitates |
| Poland |  | C8-E | Equal treatment law. | Prevention, Testing, Treatment, Care | Migrants | Indirect | Adopted | Facilitates |
| Poland | Law dated 6 April 1984 on Foundations (*Ustawa z dnia 6 kwietnia 1984 r. o fundacjach*) https://isap.sejm.gov.pl/isap.nsf/DocDetails.xsp?id=WDU19840210097 | C14.2 | Law on Foundations | Prevention, Testing, Treatment, Care | General population | Indirect | Adopted | Facilitates |
| Poland | Law dated 6 June 1997 – Penal Code (*Ustawa z dnia 6 czerwca 1997 r. – Kodeks karny*) https://isap.sejm.gov.pl/isap.nsf/DocDetails.xsp?id=WDU20220001138 | C2 | Penal Code. Articles 203 and 204 | Prevention, Testing, Treatment, Care | Sex workers | Indirect | Partially adopted | Facilitates |
| Poland | Law dated 7 April 1989 on Associations (*Ustawa z dnia 7 kwietnia 1989 r. Prawo o stowarzyszeniach*) https://isap.sejm.gov.pl/isap.nsf/DocDetails.xsp?id=WDU19890200104 | C14.1 | Law on Associations | Prevention, Testing, Treatment, Care | General population | Indirect | Adopted | Facilitates |
| Poland | Law on Patients’ Rights and Patient Rights Ombudsman (*Ustawa z dnia 6 listopada 2008 r. o prawach pacjenta i Rzeczniku Praw Pacjenta*) https://lexlege.pl/ustawa-o-prawach-pacjenta-i-rzeczniku-praw-pacjenta/ | B4 | Law on Patients’ Rights and Patient Rights Ombudsman. Article 17 | Testing | General population | Direct | Adopted | Facilitates |
| Poland |  | B5 | Law on Patients’ Rights and Patient Rights Ombudsman. Article 17 | Testing, Treatment | General population | Indirect | Not adopted | Hinders |
| Poland | Law on prevention and Combating human infectious disease (Ustawa o zapobieganiu oraz zwalczaniu zakażeń i chorób zakaźnych u ludzi) https://lexlege.pl/ustawa-o-zapobieganiu-oraz-zwalczaniu-zakazen-i-chorob-zakaznych-u-ludzi/ | B3.2 | Law on prevention and Combating human infectious disease | Prevention, Testing | PLHIV and their sexual partners | Direct | Partially adopted | Hinders |
| Poland | Law on provision of health care services from public funds (*Ustawa o świadczeniach opieki zdrowotnej finansowanych ze środków publicznych*) https://isap.sejm.gov.pl/isap.nsf/download.xsp/WDU20042102135/U/D20042135Lj.pdf | A7 | Law on provision of health care services from public funds. Article 2 | Prevention, Testing, Treatment, Care | Migrants and refugees | Indirect | Not adopted | Facilitates |
| Poland | National Health Plan 2021-2025 (*Narodowy Program Zdrowia na lata 2021-2025*) https://www.gov.pl/attachment/4ddd691b-5c1b-4492-8c1b-e7bcc6c4a382 | B7.2 | National Health Plan | Prevention | People who inject drugs | Direct | Adopted | Facilitates |
| Poland |  | B13 | National Health Plan |  | People who inject drugs, General population | Indirect | Partially adopted | N/A |
| Poland | National Program Combating Domestic Violence 2022 (Krajowy Program Przeciwdziałania Przemocy w Rodzinie na rok 2022) https://www.gov.pl/attachment/743c620c-c458-4a7e-8d01-4964bbd72264 | C13.2 | National Program Combating Domestic Violence | Prevention, Testing, Treatment, Care | General population | Direct | Adopted | Facilitates |
| Poland | National Program of Prevention of HIV Infection and Combating AIDS 2022-2026 (Krajowy Program Zapobiegania Zakażeniom HIV i Zwalczania AIDS opracowany na lata 2022 – 2026) https://aids.gov.pl/krajowy_program/2022/ | A12 | National Program of Prevention of HIV Infection and Combating AIDS |  | All PLHIV | Direct | Adopted | Facilitates |
| Poland |  | D1 | National Program of Prevention of HIV Infection and Combating AIDS | Prevention, Testing, Treatment, Care | Gay men and other MSM, Sex workers, Transgender people, People who inject drugs, Incarcerated people, General population, youth | Direct | Adopted | Facilitates |
| Poland |  | D2 | National Program of Prevention of HIV Infection and Combating AIDS | Prevention, Testing, Treatment, Care | Gay men and other MSM, Sex workers, Transgender people, People who inject drugs, Incarcerated people, General population, youth | Direct | Adopted | Facilitates |
| Poland |  | D4 | National Program of Prevention of HIV Infection and Combating AIDS. State budget section | Prevention, Testing, Treatment, Care | General population | Direct | Adopted | Facilitates |
| Poland |  | D5 | National Program of Prevention of HIV Infection and Combating AIDS | Prevention, Treatment | General population; sexual partners of PLHIV | Direct | Partially adopted | Facilitates |
| Poland |  | D8 | National Program of Prevention of HIV Infection and Combating AIDS. Monitoring activities | Treatment, Care | All PLHIV | Direct | Partially adopted | Facilitates |
| Poland | Ordinance of the Minister of National Education of 12 August 1999 on the method of school teaching and the scope of content regarding knowledge about human sexual life, the principles of conscious and responsible parenthood, the value of the family, life in the prenatal phase and methods and means of conscious procreation contained in the core curriculum of general education. (*Rozporządzenia Ministra Edukacji Narodowej z dnia 12 sierpnia 1999 r. w sprawie sposobu nauczania szkolnego oraz zakresu treści dotyczących wiedzy o życiu seksualnym człowieka, o zasadach świadomego i odpowiedzialnego rodzicielstwa, o wartości rodziny, życia w fazie prenatalnej oraz metodach i środkach świadomej prokreacji zawartych w podstawie programowej kształcenia ogólnego.*) https://www.portaloswiatowy.pl/ksztalcenie-i-wychowanie/rozporzadzenie-ministra-edukacji-narodowej-z-dnia-12-sierpnia-1999-r.-w-sprawie-sposobu-nauczania-szkolnego-oraz-zakresu-tresci-dotyczacych-wiedzy-o-zyciu-seksualnym-czlowieka-o-zasadach-swiadomego-i-odpowiedzialnego-rodzicielstwa-o-wartosci-rodziny-zycia-w-fazie-prenatalnej-oraz-metodach-i-srodkach-swiadomej-prokreacji-zawartych-w-podstawie-programowej-ksztalcenia-ogolnego-tekst-jedn.-dz.u.-z-2014-r.-poz.-395-9738.html | B8 | Ordiance on sex education |  | General population of children and adolescents | Indirect | Not adopted | Hinders |
| Poland | Patients (*pacjent.gov.pl*) https://pacjent.gov.pl/artykul/informacje-o-swiadczeniach | D6.1 | Government website for patients | Testing, Treatment, Care | All PLHIV | Indirect | Adopted | Facilitates |
| Poland | Penal code (Kodeks karny) https://isap.sejm.gov.pl/isap.nsf/download.xsp/WDU19970880553/U/D19970553Lj.pdf | C1 | The Penal Code decriminalizes consensual same-sex acts | Prevention, Testing, Treatment, Care | Gay men and other MSM | Indirect | Adopted | Facilitates |
| Poland | Penal Code Art 161 (*Kodeks Karny art. 161*) https://lexlege.pl/kk/art-161 | B3.1 | Penal Code. Article 161 | Prevention, Testing | PLHIV and their sexual partners | Direct | Partially adopted | Hinders |
| Poland |  | C7 | Penal Code. Article 161 | Prevention, Testing, Treatment, Care | PLHIV | Indirect | Partially adopted | Hinders |
| Poland | Principles of care for people infected with HIV. Recommendations of the Polish AIDS Society. (*Zasady opieki nad osobami zakażonymi HIV. Zalecenia Polskiego Towarzystwa Naukowego AIDS*) http://www.ptnaids.pl/images/pliki/zalecenia_2022_internet_OK.pdf | A10 | HIV guidelines | Treatment, Care | All PLHIV | Direct | Adopted | Facilitates |
| Poland |  | A11 | HIV guidelines. Section C, Chapter 12 | Prevention, Testing, Treatment | All women and neonates for prevention, WLHIV on pregnancy | Direct | Adopted | Facilitates |
| Poland |  | A1 | HIV guidelines. Section C, Chapter 10.3 | Treatment | All PLHIV | Direct | Adopted | Facilitates |
| Poland |  | A2 | HIV guidelines. Section C, Chapter 10.3 | Treatment | All PLHIV | Direct | Adopted | Facilitates |
| Poland |  | A3 | HIV guidelines. Section C | Treatment, Care, Prevention | All PLHIV | Direct | Adopted | Facilitates |
| Poland |  | A5 | HIV guidelines. Section C | Treatment, Care, Prevention | All PLHIV | Direct | Adopted | Facilitates |
| Poland |  | A6 | HIV guidelines. Section D | Prevention, Treatment, Care | General pediatric population (prevention); All children living with HIV | Direct | Adopted | Facilitates |
| Poland |  | A9 | HIV guidelines. Section G, Chapter 27 | Care | All PLHIV | Direct | Adopted | Facilitates |
| Poland |  | B1 | HIV guidelines. Section A | Prevention, Testing | Gay men and other MSM, Sex workers, People who inject drugs, General population | Direct | Adopted | Facilitates |
| Poland |  | B2 | HIV guidelines. Section A | Prevention, Testing | Gay men and other MSM, Sex workers, People who inject drugs, General population | Direct | Adopted | Facilitates |
| Poland |  | B6 | HIV guidelines. Section B, Chapter 5 | Prevention | Gay men and other MSM, Sex workers, People who inject drugs, Sexual partners of PLHIV and for people with STI | Direct | Adopted | Facilitates |
| Poland | Reduction of risk of health and social harms (*Ograniczanie ryzyka szkód zdrowotnych i społecznych*) https://kcpu.gov.pl/szkody-zdrowotne-i-uzaleznienia/leczenie-osob-uzaleznionych-od-substancji-psychoaktywnych-oraz-ich-bliskich/ograniczanie-ryzyka-szkod-zdrowotnych-i-spolecznych/ | B7.1 | National Health Center provides funding and substantive support for harm reduction programs. | Prevention | People who inject drugs | Direct | Adopted | Facilitates |
| Poland |  | C4 | National Health Center provides funding and substantive support for harm reduction programs. | Prevention | People who inject drugs, All drug users | Direct | Adopted | Facilitates |
| Poland |  | C5 | National Health Center provides funding and substantive support for harm reduction programs. | Prevention | People who inject drugs | Direct | Adopted | Facilitates |
| Poland |  | C6 | National Health Center provides funding and substantive support for harm reduction programs. | Prevention | People who inject drugs | Direct | Adopted | Facilitates |
| Poland | The Constitution of the Republic of Poland (*Konstytucja RP*) https://www.sejm.gov.pl/prawo/konst/angielski/kon1.htm | C16 | The Constitution. Article 18 | Prevention, Testing, Treatment, Care | Gay men and other MSM, Transgender people, Lesbians | Indirect | Not adopted | Hinders |
| Portugal | Annual Reports- HIV and AIDS Infection in Portugal (*Relatórios Anuais- Infeção VIH e SIDA em Portugal*) http://repositorio.insa.pt/simple-search?location=%2F&query=relat%C3%B3rio+vih&rpp=10&sort_by=score&order=desc | D9 | Annual Reports on HIV and AIDS Infection |  | PLHIV | Direct | Partially adopted | Facilitates |
| Portugal | Constitution of the Portuguese Republic - Article 64º Health (*Constituição da República Portuguesa - Artigo 64.º Saúde*) https://dre.pt/dre/legislacao-consolidada/decreto-aprovacao-constituicao/1976-34520775-49467775 | C11 | The Constitution. Article 64 | Prevention, Testing, Treatment, Care | General population | Indirect | Adopted | Facilitates |
| Portugal | Decree-Law No. 110/2018- Industrial Property Code (*Decreto-Lei n.º 110/2018- Código da Propriedade Industrial*) https://dre.pt/dre/legislacao-consolidada/decreto-lei/2018-117279941 | D7 | Industrial Property Code. Article 108 | Treatment | PLHIV, General Population | Indirect | Partially adopted | Facilitates |
| Portugal | Decree-Law No. 176/2012- Establishes measures that must be adopted within the scope of school pathways of students to prevent school failure and dropout (*Decreto-Lei n.º 176/2012- Estabelece medidas que devem ser adotadas no âmbito dos percursos escolares dos alunos para prevenir o insucesso e o abandono escolares*) https://dre.pt/dre/detalhe/decreto-lei/176-2012-179057 | C12 | Decree establishing school pathways of students to prevent school dropout. | Prevention | General population (aged between 6 and 18) | Indirect | Adopted | Facilitates |
| Portugal | Decree-Law No. 183/2001- Approves the general regime of prevention and risk reduction and damage minimization policies (*Decreto-Lei n.º 183/2001- Aprova o regime geral das políticas de prevenção e redução de riscos e minimização de danos*) https://dre.pt/dre/detalhe/decreto-lei/183-2001-362322 | B7 | Law on general regime of prevention and risk reduction and damage minimization policies | Prevention | People who inject drugs | Direct | Adopted | Facilitates |
| Portugal |  | C5 | Law on general regime of prevention and risk reduction and damage minimization policies. Article 3.g | Prevention | People who inject drugs | Direct | Adopted | Facilitates |
| Portugal |  | C6 | Law on general regime of prevention and risk reduction and damage minimization policies. Article 3.f | Prevention | People who inject drugs | Direct | Adopted | Facilitates |
| Portugal | Decree-Law No. 37/2022- Changes the system for charging user fees in the National Health Service (*Decreto-Lei n.º 37/2022- Altera o regime de cobrança de taxas moderadoras no Serviço Nacional de Saúde*) https://dre.pt/dre/detalhe/decreto-lei/37-2022-184054398?_ts=1653662334139 | D6 | Law on user fees. Article 8 | Prevention, Testing, Treatment, Care | PLHIV, General Population | Indirect | Adopted | Facilitates |
| Portugal | Decree-Law No. 79/2018- Allows the availability directly to the public of self-diagnosis devices for HIV, HCV and HBV infections (*Decreto-Lei n.º 79/2018- Permite a disponibilização diretamente ao público dos dispositivos de autodiagnóstico das infeções por VIH, VHC e VHB*) https://dre.pt/dre/detalhe/decreto-lei/79-2018-116673879?_ts=1680739200034 | B2 | Law on self-diagnosis devices for HIV, HCV and HBV infections. | Testing | General population | Direct | Adopted | Facilitates |
| Portugal | Dispatch No. 22144/2007- Approves the Regulation of the Specific Syringe Exchange Program (*Despacho No. 22144/2007- Aprova o Regulamento do Programa Específico de Troca de Seringas*) https://dre.pt/dre/detalhe/despacho/22144-2007-1894237 | B11.2 | Regulation of the Specific Syringe Exchange Program. Articles 5, 8 and 9 | Prevention | Incarcerated people | Direct | Adopted | Facilitates |
| Portugal | Law No. 115/2009- Code for the Execution of Sentences and Measures Depriving Liberty (*Lei n.º 115/2009- Código da Execução das Penas e Medidas Privativas da Liberdade*) https://dre.pt/dre/legislacao-consolidada/lei/2009-34515975 | A8 | Code of Execution of Sentences and Measures Depriving Liberty. Articles 7.i and 7.3 | Treatment, Prevention | Incarcerated people | Indirect | Adopted | Facilitates |
| Portugal | Law No. 12/2005- Personal genetic information and health information (*Lei n.º 12/2005- Informação genética pessoal e informação de saúde*) https://dre.pt/dre/detalhe/lei/12-2005-624463 | D8 | Personal health information. Article 4 | Testing, Treatment | General population | Indirect | Adopted | Facilitates |
| Portugal | Law No. 120/99- Reinforces the guarantees of the right to reproductive health (*Lei n.º 120/99- Reforça as garantias do direito à saúde reprodutiva*) https://dre.pt/dre/detalhe/lei/120-1999-423065 | B12 | Law on the right to reproductive health. Article 1 | Prevention | General population | Direct | Adopted | Facilitates |
| Portugal | Law No. 30/2000- Defines the legal regime applicable to the consumption of narcotic drugs and psychotropic substances, as well as the health and social protection of people who consume such substances without a medical prescription (*Lei n.º 30/2000- Define o regime jurídico aplicável ao consumo de estupefacientes e substâncias psicotrópicas, bem como a protecção sanitária e social das pessoas que consomem tais substâncias sem prescrição médica*) https://dre.pt/dre/detalhe/lei/30-2000-599720 | C3 | Legal regime applicable to the consumption of narcotic drugs and psychotropic substances. Articles 2 and 29 | Prevention, Testing, Treatment, Care | People who inject drugs | Indirect | Adopted | Facilitates |
| Portugal | Law No. 46/2006- Prohibits and punishes discrimination based on disability and the existence of an aggravated health risk (*Lei n.º 46/2006- Proíbe e pune a discriminação em razão da deficiência e da existência de risco agravado de saúde*) https://dre.pt/dre/detalhe/lei/46-2006-540797 | C8-A | Law prohibitig and punishes discrimination based on disability and the existence of an aggravated health risk. Article 1 | Prevention, Testing, Treatment, Care | PLHIV | Indirect | Adopted | Facilitates |
| Portugal | Law No. 60/2009- Establishes the system for applying sex education in schools (*Lei n.º 60/2009- Estabelece o regime de aplicação da educação sexual em meio escolar*) https://dre.pt/dre/detalhe/lei/60-2009-494016 | B8 | Law establishing the application of sex education in basic and secondary education establishments | Prevention | Students of Basic and Secondary schools | Indirect | Adopted | Facilitates |
| Portugal | Law No. 66/98- Approves the status of non-governmental development cooperation organizations (*Lei n.º 66/98- Aprova o estatuto das organizações não governamentais de cooperação para o desenvolvimento*) https://dre.pt/dre/detalhe/lei/66-1998-234902 | C14 | Law on the status of non-governmental development cooperation organizations. Articles 9 and 11 | Prevention, Testing | General population | Indirect | Adopted | Facilitates |
| Portugal | Law No. 7/2011- Creates the procedure for changing sex and first name in the civil registry and makes the seventeenth amendment to the Civil Registry Code (*Lei n.º 7/2011- Cria o procedimento de mudança de sexo e de nome próprio no registo civil e procede à décima sétima alteração ao Código do Registo Civil*) https://dre.pt/dre/detalhe/lei/7-2011-278187 | C9 | Law regulating the procedure for changing sex in the civil registry and the corresponding change of name. Articles 2 to 5 | Prevention, Testing, Treatment, Care | Transgender people | Indirect | Adopted | Facilitates |
| Portugal | Law No. 9/2010- Allows civil marriage between people of the same sex (*Lei n.º 9/2010- Permite o casamento civil entre pessoas do mesmo sexo*) https://dre.pt/dre/detalhe/lei/9-2010-332460 | C16 | Law allowing civil marriage between people of the same sex | Prevention, Testing, Treatment, Care | Gay men and other MSM, Transgender people, Lesbians | Indirect | Adopted | Facilitates |
| Portugal | Law No. 94/2017- Article 240º Discrimination and incitement to hatred and violence. (*Lei n.º 94/2017- Artigo 240º Discriminação e incitamento ao ódio e à violência*) https://dre.pt/dre/detalhe/lei/94-2017-108038373 | C8 | Anti-discrimination Law. Article 240 | Prevention, Testing, Treatment, Care | General population | Indirect | Adopted | Facilitates |
| Portugal | Law No. 94/2017- Article 240º Discrimination and incitement to hatred and violence. (*Lei n.º 94/2017- Artigo 240º Discriminação e incitamento ao ódio e à violência*) https://dre.pt/dre/detalhe/lei/94-2017-108038374 | C8-B | Anti-discrimination Law. Article 240 | Prevention, Testing, Treatment, Care | Gay men and other MSM | Indirect | Adopted | Facilitates |
| Portugal | Law No. 94/2017- Article 240º Discrimination and incitement to hatred and violence. (*Lei n.º 94/2017- Artigo 240º Discriminação e incitamento ao ódio e à violência*) https://dre.pt/dre/detalhe/lei/94-2017-108038375 | C8-D | Anti-discrimination Law. Article 240 | Prevention, Testing, Treatment, Care | Transgender people | Indirect | Adopted | Facilitates |
| Portugal | Law No. 94/2017- Article 240º Discrimination and incitement to hatred and violence. (*Lei n.º 94/2017- Artigo 240º Discriminação e incitamento ao ódio e à violência*) https://dre.pt/dre/detalhe/lei/94-2017-108038376 | C8-E | Anti-discrimination Law. Article 240 | Prevention, Testing, Treatment, Care | Migrants | Indirect | Adopted | Facilitates |
| Portugal | Law No. 95/2019 - Approves the Basic Health Law (*Lei n.º 95/2019- Aprova a Lei de Bases da Saúde*) https://dre.pt/dre/detalhe/lei/95-2019-124417108 | A7 | Basic Health Laws. Base 21 | Prevention, Testing, Treatment, Care | Migrants | Indirect | Adopted | Facilitates |
| Portugal | National Health Plan (*Plano Nacional de Saúde*) https://www.dgs.pt/documentos-em-discussao-publica/plano-nacional-de-saude-2021-2030-em-consulta-publica-ate-7-de-maio1 | B13 | National Health Plan. Objective 26 | Testing | General population | Indirect | Partially adopted | N/A |
| Portugal | National Health Program in the areas of HIV infection and AIDS (*Programa Nacional de Saúde nas áreas da infeção por VIH e SIDA*) https://www.pnvihsida.dgs.pt/nota-de-imprensa-.aspx | B11.1 | National Health Program. Note for Press 002/2021 | Prevention | Incarcerated people | Direct | Adopted | Facilitates |
| Portugal | National Health Program in the areas of HIV infection and AIDS (*Programa Nacional de Saúde nas áreas da infeção por VIH e SIDA*) https://www.pnvihsida.dgs.pt/programa-nacional111111111/programa-nacional-de-prevencao-e-controlo-da-infecao-vihsida-2012-201611/programa-nacional-de-prevencao-e-controlo-da-infecao-vihsida-2012-2016-pdf | D1 | National Program for HIV | Prevention, Testing, Treatment, Care | Gay men and other MSM, Sex workers, People who inject drugs, Incarcerated people, Migrants | Direct | Partially adopted | Facilitates |
| Portugal |  | D2 | National Program for HIV. Objective 1 |  | PLHIV, General Population | Direct | Partially adopted | Facilitates |
| Portugal | National plan to reduce addictive behaviors and addictions (*Plano Nacional para a redução dos comportamentos aditivos e das dependências*) https://www.sicad.pt/BK/Institucional/Coordenacao/Documents/Planos/SICAD_Plano_Nacional_Reducao_CAD_2013-2020.pdf | C4 | National plan to reduce addictive behaviors and addictions. Section "Objectives" | Prevention | People who inject drugs, All drug users | Direct | Adopted | Facilitates |
| Portugal | National Program for HIV/AIDS (*Programa Nacional para a Infeção VIH/SIDA*) https://www.sns24.gov.pt/tema/doencas-infecciosas/vih/prevencao-da-infecao-por-vih/#que-cuidados-devo-ter-na-utilizacao-do-preservativo | B14 | National Health Program in the areas of HIV infection and AIDS | Prevention | Gay men and other MSM, Sex workers, People who inject drugs, General population | Direct | Adopted | Facilitates |
| Portugal | Norm No. 12/DSCS/PNT. Rapid detection of Multidrug-resistant Tuberculosis (*Circular Normativa n.º 12/DSCS/PNT. Detecção rápida da Tuberculose Multirresistente*) https://www.dgs.pt/directrizes-da-dgs/normas-e-circulares-normativas/circular-normativa-n-12dscspnt-de-17072008-pdf | A9.2 | Guidelines on Rapid detection of Multidrug-resistant Tuberculosis. Section 2 | Care | People who inject drugs, Incarcerated people, Migrants, PLHIV | Direct | Adopted | Facilitates |
| Portugal | Order No. 13447-B/2015- Establishes provisions for dispensing antiretroviral therapy. (*Despacho n.º 13447-B/2015- Estabelece disposições para a dispensa da terapêutica antirretrovírica*) https://www.infarmed.pt/documents/15786/1072289/110-IB_Desp_13447-b_2015_VF.pdf | A4 | Order establishes provisions for dispensing antiretroviral therapy. Section 1 | Treatment, Prevention | PLHIV | Direct | Partially adopted | Facilitates |
| Portugal | Order No. 6716/2012- Establishes specific rules for dispensing therapies with antiretrovirals for people living with HIV/AIDS and adequate use of the SI.VIDA system (*Despacho n.º 6716/2012-Estabelece regras específicas para a dispensa das terapêuticas com antirretrovíricos às pessoas que vivem com VIH/sida e adequada utilização do sistema SI.VIDA*) https://files.dre.pt/2s/2015/11/228000001/0000200002.pdf | D5 | Order establishes provisions for dispensing antiretroviral therapy. Section 1 | Prevention, Treatment | PLHIV | Direct | Adopted | Facilitates |
| Portugal | Ordinance No. 38/2013- Establishes the conditions for installing and operating the home care service (*Portaria n.º 38/2013- Estabelece as condições de instalação e funcionamento do serviço de apoio domiciliário*) https://dre.pt/dre/detalhe/portaria/38-2013-258278 | A12 | Ordinance establishing the conditions for installing and operating the home care service. Article 2 |  | PLHIV | Indirect | Adopted | Facilitates |
| Portugal | Penal Code - Article 143º Offense to simple physical integrity (*Código Penal- Artigo 143º Ofensa à integridade física simples*) https://dre.pt/dre/legislacao-consolidada/decreto-lei/1995-34437675-59065135 | C7 | Penal Code. Article 143 | Prevention, Testing, Treatment, Care | PLHIV | Indirect | Partially adopted | Hinders |
| Portugal | Penal Code - Article 152º Domestic Violence (*Código Penal- Artigo 152º Violência Doméstica*) https://dre.pt/dre/legislacao-consolidada/decreto-lei/1995-34437675-70033887 | C13 | Penal Code. Article 152 | Prevention, Testing, Treatment, Care | General population | Indirect | Adopted | Facilitates |
| Portugal | Penal Code 1982- Article 215º Procuring/pimping (*Código Penal 1982- Artigo 215º Lenocínio*) https://dre.pt/dre/legislacao-consolidada/decreto-lei/1982-34473075 | C2 | Penal Code. Article 215 | Prevention, Testing, Treatment, Care | Sex workers | Indirect | Partially adopted | Facilitates |
| Portugal | Penal Code 2007 (*Código Penal 2007*) https://dre.pt/dre/detalhe/lei/59-2007-640142 | C1 | Penal Code. Article 207 | Prevention, Testing, Treatment, Care | Gay men and other MSM | Indirect | Adopted | Facilitates |
| Portugal | Penal Code- Article 156.º Arbitrary medical-surgical interventions and treatments (*Artigo 156.º- Intervenções e tratamentos médico-cirúrgicos arbitrários*) https://dre.pt/dre/legislacao-consolidada/decreto-lei/1995-34437675-70033910 Civil Code- Article 122º Minors (*Artigo 122.º- Menores*) and Article 123º Capabilities of Minors (*Artigo 123.º- Incapacidade dos menores*) https://dre.pt/dre/legislacao-consolidada/decreto-lei/1966-34509075 | B5 | Penal Code. Articles 122 and 123 | Testing, Treatment | People under 18 years old | Indirect | Not adopted | Hinders |
| Portugal | Resolution of the Council of Ministers No. 27/2010- Creates the National Commission for Human Rights (*Resolução do Conselho de Ministros n.º 27/2010- Cria a Comissão Nacional para os Direitos Humanos*) https://dre.pt/dre/detalhe/resolucao-conselho-ministros/27-2010-612391 | C10 | National Commission for Human Rights. Section 6 | Treatment, Care | General population | Indirect | Adopted | Facilitates |
| Portugal | Standard 025/2017, Updated at 16/05/2018- Pre-exposure prophylaxis for HIV infection in adults (*Norma 025/2017, Atualizada a 16/05/2018- Profilaxia de Pré-exposição da Infeção por VIH no Adulto*) https://www.dgs.pt/directrizes-da-dgs/normas-e-circulares-normativas/norma-n-0252017-de-28112017-pdf | B6 | Requirements for PrEP provision. Section 3 | Prevention | People who inject drugs, Partners of PLHIV, People who engage in chemsex | Direct | Adopted | Facilitates |
| Portugal | Standard 029/2017- Initial therapeutic approach to human immunodeficiency virus infection Type 1 (HIV-1) in Adolescents and Adults (*Norma 029/2017- Abordagem terapêutica inicial da infeção por vírus de imunodeficiência humana de tipo 1 (VIH-1) no Adolescente e no Adulto*) https://www.dgs.pt/directrizes-da-dgs/normas-e-circulares-normativas/norma-n-0292017-de-29122017-pdf | A1 | Standard that defines the initial therapeutic approach to HIV in Adolescents and Adults. Section 1 | Treatment, Prevention | PLHIV and their sexual partners | Direct | Adopted | Facilitates |
| Portugal |  | A2 | Standard that defines the initial therapeutic approach to HIV in Adolescents and Adults. Section 1 | Treatment, Prevention, Care | PLHIV and their sexual partners | Direct | Adopted | Facilitates |
| Portugal |  | A3 | Standard that defines the initial therapeutic approach to HIV in Adolescents and Adults. Section 4 | Treatment, Prevention | PLHIV and their sexual partners | Direct | Adopted | Facilitates |
| Portugal |  | A5 | Standard that defines the initial therapeutic approach to HIV in Adolescents and Adults. Section 15 | Treatment, Prevention | PLHIV | Direct | Adopted | Facilitates |
| Portugal |  | A9.1 | Standard that defines the initial therapeutic approach to HIV in Adolescents and Adults. Section 14 | Care | People who inject drugs, Incarcerated people, Migrants, PLHIV | Direct | Adopted | Facilitates |
| Portugal |  | A10 | Standard that defines the initial therapeutic approach to HIV in Adolescents and Adults. Sections 1 to 15 | Treatment, Prevention | PLHIV | Direct | Adopted | Facilitates |
| Portugal |  | D3 | Standard that defines the initial therapeutic approach to HIV in Adolescents and Adults. Section "Operational" | Treatment | PLHIV | Direct | Not adopted | Hinders |
| Portugal | Standard 058/2011- Diagnosis and Laboratory Screening of Human Immunodeficiency Virus Infection (*Norma 058/2011- Diagnóstico e Rastreio Laboratorial da Infeção pelo Vírus da Imunodeficiência Humana*) https://www.dgs.pt/directrizes-da-dgs/normas-e-circulares-normativas/norma-n-0582011-de-28122011-jpg.aspx | B1 | HIV testing standards. Sections 3 to 5 | Testing | Gay men and other MSM, Sex workers, People who inject drugs, Migrants, General population | Direct | Adopted | Facilitates |
| Portugal |  | B4 | HIV testing standards. Section 6 | Testing | General population | Direct | Adopted | Facilitates |
| Portugal | State Budget (*Orçamento de Estado*) https://dre.pt/dre/detalhe/lei/12-2022-185224662 | D4 | State Budget. Article 205 | Prevention, Testing, Treatment, Care | General population | Direct | Adopted | Facilitates |
| Romania | Civil Code of July 17, 2009 (Law No. 287/2009**) (*CODUL CIVIL din 17 iulie 2009 (Legea nr. 287/2009***)) https://legislatie.just.ro/Public/DetaliiDocument/109884 | C16 | Civil Code. Article 259 | Prevention, Testing, Treatment, Care | Gay men and other MSM, Transgender people, Lesbians | Indirect | Not adopted | Hinders |
| Romania | Constitution of November 21, 1991 (*CONSTITUȚIE din 21 noiembrie 1991*) https://legislatie.just.ro/Public/DetaliiDocument/1413 | C8 | The Constitution. Article 16 | Prevention, Testing, Treatment, Care | General population | Indirect | Adopted | Facilitates |
| Romania |  | C11 | The Constitution. Article 34 | Prevention, Testing, Treatment, Care | General population | Indirect | Adopted | Facilitates |
| Romania | Emergency ordinance no. 89/2001 for the amendment and completion of some provisions of the Criminal Code regarding crimes related to sexual life https://lege5.ro/Gratuit/gmzdanrr/ordonanta-de-urgenta-nr-89-2001-pentru-modificarea-si-completarea-unor-dispozitii-din-codul-penal-referitoare-la-infractiuni-privind-viata-sexuala | C1 | Criminal Code Ammendment. Article 200 repealed | Prevention, Testing, Treatment, Care | Gay men and other MSM | Indirect | Adopted | Facilitates |
| Romania | Guide of August 9, 2019 regarding HIV infection in pregnancy (*Ghid din 9 august 2019 privind infecția HIV în sarcină*) https://legislatie.just.ro/Public/DetaliiDocumentAfis/218537 | A11 | HIV infection in pregnancy guidelines | Testing, Treatment, Care, Prevention | WLHIV and newborns | Direct | Adopted | Facilitates |
| Romania | Law no. 119 of October 16, 1996 regarding civil status documents (*Lege nr. 119 din 16 octombrie 1996 cu privire la actele de stare civilă)* https://legislatie.just.ro/Public/DetaliiDocument/196564#A255 | C9 | Law regulating civil status documents in general. Article 43 | Prevention, Testing, Treatment, Care | Transgender people | Indirect | Adopted | Facilitates |
| Romania | Law no. 143 of July 26, 2000 on preventing and combating drug trafficking and illicit consumption (*Lege nr. 143 din 26 iulie 2000 privind prevenirea și combaterea traficului și consumului ilicit de droguri*) https://legislatie.just.ro/Public/DetaliiDocumentAfis/253066 | C3 | Law establishing the national legal framework to combat drug trafficking and ilicit consumption | Prevention, Testing, Treatment, Care | People who inject drugs | Indirect | Not adopted | Hinders |
| Romania | Law no. 217 of May 22, 2003 to prevent and combat domestic violence (*Lege nr. 217 din 22 mai 2003 pentru prevenirea și combaterea violenței domestice*) https://legislatie.just.ro/Public/DetaliiDocument/228251 | C13.2 | Law on preventing and fighting against family violence defines domestic violence | Prevention, Testing, Treatment, Care | General population | Indirect | Partially adopted | Hinders |
| Romania | Law no. 584 of October 29, 2002 regarding the measures to prevent the spread of the AIDS disease in Romania and protection of people infected with HIV or sick with AIDS (*Lege nr.584 din 29 octombrie 2002 privind măsurile de prevenire a răspândirii maladiei SIDA în România şi de protecţie a persoanelor infectate cu HIV sau bolnave de SIDA*) https://legislatie.just.ro/Public/DetaliiDocument/39744 | A12 | Law on preventive measures against HIV and establishes protections for PLHIV. Chapter III | Care | PLHIV | Direct | Adopted | Facilitates |
| Romania |  | C8-A.2 | Law on preventive measures against HIV and establishes protections for PLHIV. Article 3 | Prevention, Testing, Treatment, Care | PLHIV | Indirect | Adopted | Facilitates |
| Romania |  | C14 | Law on preventive measures against HIV and establishes protections for PLHIV. Chapter VI | Prevention, Testing, Treatment, Care | General population | Indirect | Adopted | Facilitates |
| Romania |  | D4.1 | Law on preventive measures against HIV and establishes protections for PLHIV. Chapter VI | Prevention, Testing, Treatment, Care | General population | Direct | Adopted | Facilitates |
| Romania | Law no. 64 of October 11, 1991 regarding patents (*Lege nr. 64 din 11 octombrie 1991 privind brevetele de invenţie*) https://legislatie.just.ro/Public/DetaliiDocumentAfis/38939 | D7 | Law regarding patents. Articles 48 and 49 | Treatment, Care | PLHIV | Indirect | Partially adopted | Facilitates |
| Romania | Law no. 9 of January 5, 2018 for the amendment and completion of Law no. 35/1997 regarding the organization and operation of the People's Advocate institution (*Lege nr. 9 din 5 ianuarie 2018 pentru modificarea și completarea Legii nr. 35/1997 privind organizarea și funcționarea instituției Avocatul Poporului*) https://legislatie.just.ro/Public/DetaliiDocument/196418 | C10 | Ombudsman law | Treatment, Care | Gay men and other MSM, Sex workers, Transgender people, Incarcerated people, General population | Indirect | Adopted | Facilitates |
| Romania | Law no. 95 of April 14, 2006 on health care reform (*Lege nr. 95 din 14 aprilie 2006 privind reforma în domeniul sănătății*) https://legislatie.just.ro/Public/DetaliiDocument/170974 | B5 | Law on health care reform. Title XVI, Chapter III | Testing, Treatment | Minors | Indirect | Not adopted | Hinders |
| Romania |  | D5 | Law on health care reform. Title VIII, Chapter II, Section 2, Article 232 | Prevention, Testing, Treatment, Care | PLHIV | Direct | Partially adopted | Facilitates |
| Romania |  | D6 | Law on health care reform. Title VIII | Treatment, Care | PLHIV | Direct | Adopted | Facilitates |
| Romania |  | D8 | Law on health care reform. Title VIII, Chapter II, Section 2, Article 230 | Treatment, Care | PLHIV | Indirect | Partially adopted | Facilitates |
| Romania | Law on national education no. 1 of January 5, 2011 (*Lege educației naționale nr. 1 din 5 ianuarie 2011*) https://legislatie.just.ro/Public/DetaliiDocument/249377#A2738 | C12 | Law on national education. Articles 3 and 16 | Prevention | Children and youth | Indirect | Adopted | Facilitates |
| Romania | National Guide dated January 4, 2018 on TB-HIV case management 2017 (*Ghid National din 4 ianuarie 2018 privind managementul cazurilor TB-HIV 2017*) https://legislatie.just.ro/Public/DetaliiDocument/206252 | A9 | TB-HIV case management guidelines. Chapter 5 | Testing, Treatment | PLHIV | Direct | Adopted | Facilitates |
| Romania | National Health Strategy 2023-2030 (*Strategia Națională De Sănătate 2023-2030 „Pentru sănătate, împreună”*) https://ms.ro/media/documents/Anexa_1_-_SNS.pdf | B13 | National Health Strategy | Prevention, Testing, Treatment, Care | General population | Direct | Adopted | Facilitates |
| Romania | National Program "Education for Health in the Romanian School" (*Program Naţional ”Educaţia Pentru Sănătate în Şcoala Românească”*) https://gov.ro/fisiere/programe_fisiere/program-educatie-sanatate.pdf | B8 | National Program on Education for Health. Section II. Principle 5 | Prevention | Children and youth | Indirect | Adopted | Facilitates |
| Romania | National Strategy from March 16, 2022 in the field of drugs 2022-2026 and the Action Plan for the implementation of the National Strategy in the field of drugs 2022-2026 (*Strategie Națională din 16 martie 2022 în domeniul drogurilor 2022-2026 și Planul de acțiune pentru implementarea Strategiei naționale în domeniul drogurilor 2022-2026*) https://legislatie.just.ro/Public/DetaliiDocument/253794 | B7 | National Antidrug Strategy | Prevention | People who inject drugs | Direct | Adopted | Facilitates |
| Romania |  | C4 | National Antidrug Strategy | Prevention, Testing, Care | People who inject drugs, All drug users | Direct | Adopted | Facilitates |
| Romania | National Strategy from March 30, 2022 on social inclusion and poverty reduction for the period 2022-2027 (*Strategie Națională din 30 martie 2022 privind incluziunea socială și reducerea sărăciei pentru perioada 2022-2027*) https://legislatie.just.ro/Public/DetaliiDocument/254234#A1 | B9 | National Strategy for Social Inclusion and Poverty Reduction. Specific Objective 3 | Prevention, Testing, Treatment, Care | Sex workers | Indirect | Adopted | Facilitates |
| Romania | National Strategy from November 29, 2022 for the surveillance, control and prevention of cases of HIV/AIDS infection in the period 2022-2030 (*Strategia națională din 29 noiembrie 2022 pentru supravegherea, controlul și prevenirea cazurilor de infecție cu HIV/SIDA în perioada 2022 –2030*) https://legislatie.just.ro/Public/DetaliiDocumentAfis/262842 | A1 | National Strategy for the surveillance, control and prevention of cases of HIV/AIDS infection. Section II.2 | Treatment, Prevention | PLHIV and their sexual partners | Direct | Adopted | Facilitates |
| Romania |  | A5 | National Strategy for the surveillance, control and prevention of cases of HIV/AIDS infection. Specific objective 4.3 | Treatment, Prevention | PLHIV and their sexual partners | Direct | Partially adopted | Hinders |
| Romania |  | B2 | National Strategy for the surveillance, control and prevention of cases of HIV/AIDS infection. Chapter II | Testing | General population | Direct | Not adopted | Hinders |
| Romania |  | B4 | National Strategy for the surveillance, control and prevention of cases of HIV/AIDS infection. Directive Principle 8 | Testing | General population | Direct | Adopted | Facilitates |
| Romania |  | B6 | National Strategy for the surveillance, control and prevention of cases of HIV/AIDS infection. Specific objective 4 | Prevention |  | Direct | Not adopted | Hinders |
| Romania |  | B10 | National Strategy for the surveillance, control and prevention of cases of HIV/AIDS infection. Specific objective 1 | Prevention, Testing, Care | Gay men and other MSM | Direct | Adopted | Facilitates |
| Romania |  | B11 | National Strategy for the surveillance, control and prevention of cases of HIV/AIDS infection. Specific objective 1 | Prevention | Incarcerated people | Direct | Adopted | Facilitates |
| Romania |  | D1 | National Strategy for the surveillance, control and prevention of cases of HIV/AIDS infection | Prevention, Testing, Treatment, Care | Gay men and other MSM, Sex workers, Transgender people, People who inject drugs, Incarcerated people, Several groups are identified at increased risk, as defined in the Strategy | Direct | Adopted | Facilitates |
| Romania |  | D2 | National Strategy for the surveillance, control and prevention of cases of HIV/AIDS infection. Chapter 7 | Prevention, Testing, Treatment, Care | Gay men and other MSM, Sex workers, Transgender people, People who inject drugs, Incarcerated people, Young people | Direct | Adopted | Facilitates |
| Romania |  | D4.2 | National Strategy for the surveillance, control and prevention of cases of HIV/AIDS infection | Prevention, Testing, Treatment, Care | Gay men and other MSM, Sex workers, Transgender people, People who inject drugs, Incarcerated people, General population | Direct | Adopted | Facilitates |
| Romania | Ordinance no. 137 of August 31, 2000 on the prevention and sanctioning of all forms of discrimination (*Ordonanță nr. 137 din 31 august 2000*) https://legislatie.just.ro/Public/DetaliiDocument/79377 | C8-A.1 | Anti-discrimination regulation | Prevention, Testing, Treatment, Care | PLHIV | Indirect | Adopted | Facilitates |
| Romania |  | C8-B | Anti-discrimination regulation | Prevention, Testing, Treatment, Care | Gay men and other MSM | Indirect | Adopted | Facilitates |
| Romania |  | C8-C | Anti-discrimination regulation | Prevention, Testing, Treatment, Care | People who inject drugs | Indirect | Adopted | Facilitates |
| Romania |  | C8-D | Anti-discrimination regulation | Prevention, Testing, Treatment, Care | Transgender people | Indirect | Adopted | Facilitates |
| Romania |  | C8-E | Anti-discrimination regulation | Prevention, Testing, Treatment, Care | Migrants | Indirect | Adopted | Facilitates |
| Romania |  | C8-F | Anti-discrimination regulation | Prevention, Testing, Treatment, Care | Incarcerated people | Indirect | Adopted | Facilitates |
| Romania | Penal Code of July 17, 2009 (Law Nr. 286/2009) (*Codul Penal din 17 iulie 2009 (Legea nr. 286/2009)*) https://legislatie.just.ro/Public/DetaliiDocument/109855 | C2 | Penal Code. Article 213 | Prevention, Testing, Treatment, Care | Sex workers | Indirect | Partially adopted | Facilitates |
| Romania |  | C13.1 | Penal Code. Article 199 | Prevention, Testing, Treatment, Care | General population | Indirect | Partially adopted | Facilitates |
| Romania |  | C7 | Penal Code. Article 354 | Prevention, Testing, Treatment, Care | PLHIV | Indirect | Partially adopted | Hinders |
| Romania | Regulation of March 10, 2016 on the application of Law no. 254/2013 on the execution of punishments and custodial measures ordered by judicial bodies during the criminal process (*Regulament din 10 martie 2016 de aplicare a Legii nr. 254/2013 privind executarea pedepselor și a măsurilor privative de libertate dispuse de organele judiciare în cursul procesului penal*) https://legislatie.just.ro/Public/DetaliiDocumentAfis/181314 | A8 | Regulation determining the conditions of detention for incarcerated people. Chapter V, Article 152 | Prevention, Testing, Treatment, Care | Incarcerated people | Indirect | Adopted | Facilitates |
| Spain | Consensus document for the follow-up of HIV infection in relationship with reproduction, pregnancy, birth, and prophylaxis of vertical transmission to the exposed child (*Documento de consenso para el seguimiento de la infección por VIH en relación con la reproducción, embarazo, parto y profilaxis de la transmisión vertical del niño expuesto*) https://gesida-seimc.org/wp-content/uploads/2018/05/gesida_VIH_embarazo.pdf | A11.2 | Consensus document for the follow-up of HIV infection in pregnancy | Testing, Treatment, Care, Prevention | Reproductive age women, Pregnant WLHIV, Newborns exposed to HIV | Direct | Adopted | Facilitates |
| Spain | Consensus document to improve adherence to pharmacotherapy in patients with human immunodeficiency virus infection in antiretroviral treatment (*Documento de consenso para mejorar la adherencia a la farmacoterapia en pacientes con infección por el virus de la inmunodeficiencia humana en tratamiento antirretroviral*) https://gesida-seimc.org/wp-content/uploads/2020/04/GUIA_GESIDA_febrero_2020_Adherencia.pdf | A3.2 | Consensus document to improve ART adherence | Treatment, Care, Prevention | PLHIV | Direct | Adopted | Facilitates |
| Spain | Criminal Code (*Código Criminal*) https://www.mjusticia.gob.es/es/AreaTematica/DocumentacionPublicaciones/Documents/Criminal_Code_2016.pdf | C7 | Criminal Code. Article 147 | Prevention, Testing, Treatment, Care | PLHIV | Indirect | Partially adopted | Hinders |
| Spain | Early HIV diagnosis: Pharmaceutical action guide in the dispensing of medical devices for HIV self-diagnosis (*Diagnóstico precoz del VIH: Guía de actuación farmacéutica en la dispensación de productos sanitarios para autodiagnóstico del VIH*) https://www.sanidad.gob.es/ciudadanos/enfLesiones/enfTransmisibles/sida/docs/Diagnostico_Precoz_VIH.pdf | B2.2 | Pharmaceutical action guide in the dispensing of medical devices for HIV self-diagnosis | Testing | Anyone suspecting risk of HIV exposure. | Direct | Adopted | Facilitates |
| Spain | Epidemiological surveillance of HIV and AIDS in Spain 2021 (Vigilancia epidemiológica del VIH y SIDA en España 2021) https://www.sanidad.gob.es/ciudadanos/enfLesiones/enfTransmisibles/sida/vigilancia/doc/Informe_VIH_SIDA_2022_CCAA.pdf | D9 | National Epidemiological Surveillance Network. Chapter IV | Prevention, Testing, Treatment, Care | PLHIV | Direct | Partially adopted | Facilitates |
| Spain | General State Budgets - Approved Budget: Section 26. Ministry of Health. Program budget (*Presupuestos Generales del Estado - Presupuesto aprobado: Sección 26. Ministerio de Sanidad. Presupuesto por programas)* https://www.sepg.pap.hacienda.gob.es/Presup/PGE2023Ley/MaestroDocumentos/PGE-ROM/N_23_E_R_31_126_1_1_2.htm | D4 | General State Budget does not include a line item for the HIV/AIDS program. | Prevention, Testing, Treatment, Care | General population | Indirect | Not adopted | Hinders |
| Spain | GeSIDA/National AIDS Plan consensus document regarding antiretroviral treatment in adults infected with the human immunodeficiency virus (Updated January 2022) *(Documento de consenso de GeSIDA/Plan Nacional sobre el Sida respecto al tratamiento antirretroviral en adultos infectados por el virus de la inmunodeficiencia humana (Actualización enero 2022)*) https://www.sanidad.gob.es/ciudadanos/enfLesiones/enfTransmisibles/sida/publicaciones/2022/Consenso_TAR_2022.pdf | A1.1 | HIV treatment guidelines. Section 3.1 | Treatment, Care, Prevention | PLHIV | Direct | Adopted | Facilitates |
| Spain |  | A2 | HIV treatment guidelines. Section 3.1 | Treatment, Care, Prevention | PLHIV | Direct | Adopted | Facilitates |
| Spain |  | A3.1 | HIV treatment guidelines. Section 3.2 | Treatment, Care, Prevention | PLHIV | Direct | Adopted | Facilitates |
| Spain |  | A5 | HIV treatment guidelines. Table 1 | Treatment, Care, Prevention | PLHIV | Direct | Adopted | Facilitates |
| Spain |  | A10 | HIV treatment guidelines | Treatment, Care, Prevention | PLHIV | Direct | Adopted | Facilitates |
| Spain |  | A11.1 | HIV treatment guidelines. Section 7.3 | Treatment, Care, Prevention | WLHIV who are pregnant or are planning to become pregnant. | Direct | Adopted | Facilitates |
| Spain | Guide for conducting rapid HIV tests in community settings (Guía para la realización de pruebas rápdas del VIH en entornos comunitarios) https://www.sanidad.gob.es/bibliotecaPub/repositorio/libros/28690_guia_para_la_realizacion_de_pruebas_rapidas_del_VIH_en_entornos_comunitarios.pdf | B1.3 | Rapid HIV tests guidelines | Testing | Gay men and other MSM, Sex workers, Transgender people, People who inject drugs, People over 16 that request a test, Sexual partners of PLHIV, Partners and clients of sex workers, Heterosexuals with more than one sexual partners and/or risk practices, People who have suffered sexual assault, People with accidental exposure to HIV | Direct | Adopted | Facilitates |
| Spain | Law 16/2003, of 28 May, on cohesion and quality of the National Health System (*Ley 16/2003, de 28 de mayo, de cohesión y calidad del Sistema Nacional de Salud.*) https://www.boe.es/eli/es/l/2003/05/28/16 | D5 | Law protecting universal access to healthcare | Treatment, Care, Prevention, Testing | General population, PLHIV | Indirect | Adopted | Facilitates |
| Spain | Law 24/2015, of 24 July, of Patents. Last update published 4 July 2018. *(Ley 24/2015, de 24 de julio, de Patentes*. Última actualización publicda 04/07/2018) https://www.boe.es/eli/es/l/2015/07/24/24/con | D7 | Law on patents and industrial property in general. Article 95 | Treatment | PLHIV | Indirect | Partially adopted | Facilitates |
| Spain | Law 4/2023, of 28 February , for the real and effective equality of trans people and for the guarantee of the rights of LGTBI people.(*Ley 4/2023, de 28 de febrero, para la igualdad real y efectiva de las personas trans y para la garantía de los derechos de las personas LGTBI.*) https://www.boe.es/eli/es/l/2023/02/28/4 | C9 | LGBTI rights law. Section 2 | Prevention, Testing, Treatment, Care | Transgender people, Gay men and other MSM | Indirect | Adopted | Facilitates |
| Spain | Law 77/1978, of December 26, modifying the Law on Dangerousness and Social Rehabilitation and its Regulations. (Ley 77/1978, de 26 de diciembre, de modificación de la Ley de Peligrosidad y Rehabilitación Social y de su Reglamento.) https://www.boe.es/eli/es/l/1978/12/26/77 | C1 | Law on Dangerousness. Article 2 repealed | Prevention, Testing, Treatment, Care | Gay men and other MSM | Indirect | Adopted | Facilitates |
| Spain | Manual for the implementation of an HIV Pre-exposure Prophylaxis Program in Spain. Update December 2021 (*Manual de implementación de un Programa de Profilaxis Preexposición al VIH en España. Actualización diciembre de 2021*) https://www.sanidad.gob.es/ciudadanos/enfLesiones/enfTransmisibles/sida/PrEP/Manual_PrEP_FINAL.pdf | B6.1 | PrEP implementation program | Prevention | Gay men and other MSM, Transgender people, people who use drugs during sex, People with over 10 sex partners in the last year, People who have practiced anal sex without a condom in the last year, People who have used post-exposure prophylaxis several times during the last year, People with at least one bacterial STI in the last year. | Direct | Adopted | Facilitates |
| Spain | National Strategy on Addictions 2017-2024 - Addictions Action Plan 2021-2024 *(Estrategia Nacional sobre Adicciones 2017-2024 - Plan de Acción sobre Adicciones 2021-2024*) https://pnsd.sanidad.gob.es/pnsd/planAccion/docs/AddictionsActionPlan_2021-2024.pdf | C4 | National Strategy on Addictions | Prevention | People who inject drugs, All drug users | Direct | Adopted | Facilitates |
| Spain |  | C6 | National Strategy on Addictions. Action Area 3, Goal 1 | Prevention | People who inject drugs, Incarcerated people, People who practice chemsex, Methamphetamine users | Direct | Adopted | Facilitates |
| Spain | Order of December 18, 2000 creating a file with personal data, managed by the Ministry of Health and Consumption, relating to the Information System on New Infections (SINIVIH) (*Orden de 18 de diciembre de 2000 por la que se crea un fichero con datos de carácter personal, gestionado por el Ministerio de Sanidad y Consumo, relativo al Sistema de Información sobre Nuevas Infecciones (SINIVIH)*) https://www.boe.es/buscar/doc.php?id=BOE-A-2001-927 | D8 | Information System on New Infections | Treatment | PLHIV | Direct | Partially adopted | Facilitates |
| Spain | Organic Law 1/1979, of 26 September, General Penitentiary. Latest update published 5 June 2021. (*Ley Orgánica 1/1979, de 26 de septiembre, General Penitenciaria. Última acctualización publicada el 05/06/2021*) https://www.boe.es/eli/es/lo/1979/09/26/1/con | A8 | Law regulating general aspects of penitentiary institutions. Title II, Chapter III | Treatment, Care | Incarcerated people | Indirect | Adopted | Facilitates |
| Spain | Organic Law 1/2004, of 28 December, on Comprehensive Protection Measures against Gender Violence. (*Ley Orgánica 1/2004, de 28 de diciembre, de Medidas de Protección Integral contra la Violencia de Género.*) https://www.boe.es/eli/es/lo/2004/12/28/1/con | C13 | Comprehensive Protection Measures against Gender Violence | Prevention, Testing, Treatment, Care | Sex workers, Transgender people, Women | Indirect | Adopted | Facilitates |
| Spain | Organic Law 1/2023, of 28 February, which modifies Organic Law 2/2010, of March 3, on sexual and reproductive health and the voluntary interruption of pregnancy. *(Ley Orgánica 1/2023, de 28 de febrero, por la que se modifica la Ley Orgánica 2/2010, de 3 de marzo, de salud sexual y reproductiva y de la interrupción voluntaria del embarazo*.) https://www.boe.es/eli/es/lo/2023/02/28/1/con | B8 | Law on sexual and reproductive health and the voluntary termination of pregnancy | Prevention | School-aged children and youth. | Indirect | Adopted | Facilitates |
| Spain |  | B12 | Law on sexual and reproductive health and the voluntary termination of pregnancy | Prevention, Treatment | General population; Young people. | Direct | Adopted | Facilitates |
| Spain | Organic Law 10/1995, of 23 November, of the Penal Code. Last update published 29 March 2023. (Ley Orgánica 10/1995, de 23 de noviembre, del Código Penal. Última actualización publicada el 29/03/2023. https://www.boe.es/eli/es/lo/1995/11/23/10/con | C2 | Penal Code. Title VIII and Chapter V | Prevention, Testing, Treatment, Care | Sex workers | Indirect | Partially adopted | Hinders |
| Spain | Organic Law 3/1981, of 6 April, on the Ombudsman. Last update published 4 November 2009 (*Ley Orgánica 3/1981, de 6 de abril, del Defensor del Pueblo. Última actualización publicada el 04/11/2009*) https://www.boe.es/eli/es/lo/1981/04/06/3/con | C10 | Law on the Ombudsman | Treatment, Care | General population | Indirect | Adopted | Facilitates |
| Spain | Organic Law 4/2015, of 30 March, for the protection of citizen security. Last update published 23 February 2021. (*Ley Orgánica 4/2015, de 30 de marzo, de protección de la seguridad ciudadana. Última actualización publicada 23/02/2021)* https://www.boe.es/eli/es/lo/2015/03/30/4/con | C3 | Law for the protection of citizen security. Article 36 | Prevention, Testing, Treatment, Care | People who inject drugs | Indirect | Not adopted | Hinders |
| Spain | Organic Law 6/2022, of 12 July, complementary to Law 15/2022, of 12 July, comprehensive for equal treatment and non-discrimination, amending Organic Law 10/1995, of 23 November, of the Penal Code. (*Ley Orgánica 6/2022, de 12 de julio, complementaria de la Ley 15/2022, de 12 de julio, integral para la igualdad de trato y la no discriminación, de modificación de la Ley Orgánica 10/1995, de 23 de noviembre, del Código Penal.*) https://www.boe.es/eli/es/lo/2022/07/12/6/con | C8-B | Anti-discrimination law. Articles 22 and 510 modified | Prevention, Testing, Treatment, Care | Gay men and other MSM | Indirect | Adopted | Facilitates |
| Spain |  | C8-C | Anti-discrimination law. Articles 22 and 510 modified | Prevention, Testing, Treatment, Care | People who inject drugs | Indirect | Adopted | Facilitates |
| Spain |  | C8-D | Anti-discrimination law. Articles 22 and 510 modified | Prevention, Testing, Treatment, Care | Transgender people | Indirect | Adopted | Facilitates |
| Spain |  | C8-E | Anti-discrimination law. Articles 22 and 510 modified | Prevention, Testing, Treatment, Care | Migrants (including unaccompanied minors), | Indirect | Adopted | Facilitates |
| Spain | Plan for the prevention and control of infection by HIV and STI 2021-2030 in Spain (Plan de prevención y control de la infección por el VIH y las ITS 2021-2030 en España) https://www.sanidad.gob.es/ciudadanos/enfLesiones/enfTransmisibles/sida/planNalSida/Plan_de_Prevencion_y_Control1.pdf | A1.2 | Plan for the prevention and control of infection by HIV and STI. Strategic objective 3 | Treatment, Care | PLHIV | Direct | Adopted | Facilitates |
| Spain |  | A4 | Plan for the prevention and control of infection by HIV and STI | Treatment, Care | PLHIV | Direct | Not adopted | Facilitates |
| Spain |  | B1.1 | Plan for the prevention and control of infection by HIV and STI. Strategic objective 2 | Testing, Prevention | Gay men and other MSM, Sex workers, Transgender people, People who inject drugs, Incarcerated people, migrants (including unaccompanied minors), Insert other national priority population 2: people who use drugs during sex, General population | Direct | Adopted | Facilitates |
| Spain |  | B2.1 | Plan for the prevention and control of infection by HIV and STI. Strategic objective 2 | Testing |  | Direct | Adopted | Facilitates |
| Spain |  | B7 | Plan for the prevention and control of infection by HIV and STI. Line of Action 1.6 | Prevention | People who inject drugs, Incarcerated people | Direct | Adopted | Facilitates |
| Spain |  | B9 | Plan for the prevention and control of infection by HIV and STI. Line of Action 1.1 | Prevention | Sex workers | Direct | Partially adopted | Facilitates |
| Spain |  | B10 | Plan for the prevention and control of infection by HIV and STI. Line of Action 1.1 | Prevention | Gay men and other MSM | Direct | Adopted | Facilitates |
| Spain |  | C5.1 | Plan for the prevention and control of infection by HIV and STI. Line of Action 1.6 | Prevention | People who inject drugs, Incarcerated people | Direct | Adopted | Facilitates |
| Spain |  | D1 | Plan for the prevention and control of infection by HIV and STI | Prevention, Testing, Treatment, Care | Gay men and other MSM, Sex workers, Transgender people, People who inject drugs, Incarcerated people, migrants (including unaccompanied minors), Insert other national priority population 2: people who use drugs during sex, General population. | Direct | Adopted | Facilitates |
| Spain |  | D2.2 | Plan for the prevention and control of infection by HIV and STI. Chapter 10 | Prevention, Testing, Treatment, Care | Gay men and other MSM, Sex workers, Transgender people, People who inject drugs, Incarcerated people, migrants (including unaccompanied minors), Insert other national priority population 2: people who use drugs during sex, General population. | Direct | Adopted | Facilitates |
| Spain | Public Health Strategy 2022 (Estrategia de Salud Pública 2022) https://www.sanidad.gob.es/ciudadanos/pdf/Estrategia_de_Salud_Publica_2022___Pendiente_de_NIPO.pdf | B13 | Public Health Strategy | Prevention | General population | Indirect | Adopted | Facilitates |
| Spain | Recommendations guide for early diagnosis of HIV in the health sector (*Guía de recomendaciones para el diagnóstico precoz del VIH en el ámbito sanitario*) https://www.sanidad.gob.es/ciudadanos/enfLesiones/enfTransmisibles/sida/docs/GuiaRecomendacionesDiagnosticoPrecozVIH.pdf | B1.2 | Recommendations guide for early diagnosis of HIV in the health sector | Testing | Gay men and other MSM, Sex workers, Transgender people, migrants (including unaccompanied minors), People who inject drugs, General population (sexually active adults), People who may have been subject to occupational exposure. | Direct | Adopted | Facilitates |
| Spain | Recommendations on Pre-Exposure Prophylaxis for the prevention of HIV infection in Spain - January 2023 update. (Recomendaciones sobre la profilaxis pre-exposición para la prevención de la infección por VIH en España - Actualización Enero 2023. https://gesida-seimc.org/wp-content/uploads/2023/03/Guia-profilaxis.pdf | B6.2 | PrEP Recommendations | Prevention | Gay men and other MSM, Transgender people, Sex workers, People who inject drugs, Adolescents from age 16, Cis men and women at risk of HIV. | Direct | Adopted | Facilitates |
| Spain | Royal Decree 1302/2018, of 22 October, which modifies Royal Decree 954/2015, of October 23, which regulates the indication, use and authorization of dispensing of medicines and medical devices for human use by part of the nurses. (*Real Decreto 1302/2018, de 22 de octubre, por el que se modifica el Real Decreto 954/2015, de 23 de octubre, por el que se regula la indicación, uso y autorización de dispensación de medicamentos y productos sanitarios de uso humano por parte de los enfermeros.)* https://www.boe.es/eli/es/rd/2018/10/22/1302 | D3 | Decree regulating the indication, use and authorization of dispensing of medicines and medical devices for human use by part of the nurses | Treatment, Care | PLHIV | Indirect | Not adopted | Hinders |
| Spain | Royal Decree 2210/1995, of 28 December, which creates the national epidemiological surveillance network. (Real Decreto 2210/1995, de 28 de diciembre, por el que se crea la red nacional de vigilancia epidemiológica.) https://www.boe.es/eli/es/rd/1995/12/28/2210/con | D2.1 | Decree creating the national epidemiological surveillance network. Chapter IV | Prevention, Testing, Treatment, Care | PLHIV | Direct | Adopted | Facilitates |
| Spain | Royal Decree of 24 July, 1889 by which the Civil Code is published. Last update published 1 March 2023. (Real Decreto de 24 de julio de 1889 por el que se publica el Código Civil. Última actualización publicada el 01/03/2023. https://www.boe.es/eli/es/rd/1889/07/24/(1)/con | C16 | Civil Code. Title IV, Chapter II, Article 44 | Prevention, Testing, Treatment, Care | Gay men and other MSM, Transgender people, Lesbians | Indirect | Adopted | Facilitates |
| Spain | Royal Decree-Law 16/2012, of April 20, on urgent measures to guarantee the sustainability of the National Health System and improve the quality and safety of its services. (*Real Decreto-ley 16/2012, de 20 de abril, de medidas urgentes para garantizar la sostenibilidad del Sistema Nacional de Salud y mejorar la calidad y seguridad de sus prestaciones.)* https://www.boe.es/eli/es/rdl/2012/04/20/16/con | D6 | Decree on urgent measures to guarantee the sustainability of the National Health System. Article 2 | Testing, Treatment, Care | General population | Indirect | Adopted | Facilitates |
| Spain | Royal Decree-Law 7/2018, of 27July, on universal access to the National Health System *(Real Decreto-ley 7/2018, de 27 de julio, sobre el acceso universal al Sistema Nacional de Salud*) https://www.boe.es/eli/es/rdl/2018/07/27/7 | A7 | Decree on universal access to the National Health System. Article 3 modified | Treatment, Care, Testing, Prevention | Migrants (including unaccompanied minors), | Indirect | Adopted | Facilitates |
| Spain | Social pact for non-discrimination and equality in treatment associated with HIV. (*Pacto social por la no discriminación y la igualdad de trato asociada al VIH)* https://www.sanidad.gob.es/ciudadanos/enfLesiones/enfTransmisibles/sida/doc/pactoSocial_27Feb19.pdf | C8-A | Social pact for non-discrimination and equality in treatment associated with HIV | Prevention, Testing, Treatment, Care | PLHIV | Indirect | Adopted | Facilitates |
| Spain | Spanish Constitution. Last update publishes 27 September 2011 (*Constitución Española. Última actualización publicada el 27/09/2011*) https://www.boe.es/eli/es/c/1978/12/27/(1)/con | C8 | The Constitution. Article 14 | Prevention, Testing, Treatment, Care | Gay men and other MSM, Sex workers, Transgender people, People who inject drugs, Incarcerated people, migrants (including unaccompanied minors), | Indirect | Adopted | Facilitates |
| Spain |  | C11 | The Constitution. Article 43 | Prevention, Testing, Treatment, Care | General population | Indirect | Adopted | Facilitates |
| Spain | Syringes exchange in the penitentiary environment. *(Intercambio de jeringuillas en el medio penitenciario) https://www.sanidad.gob.es/ciudadanos/enfLesiones/enfTransmisibles/sida/prevencion/actividadesEnPrisiones/docs/programaMarco.pdf* | B11 | Syringes exchange in the penitentiary environment program | Prevention | Incarcerated people | Direct | Partially adopted | Facilitates |
| Spain |  | C5.2 | Syringes exchange in the penitentiary environment program | Prevention | Incarcerated people | Direct | Adopted | Facilitates |
| Spain - Catalonia | Action plan for the fight against HIV and other STI 2021-2030 (*Pla d’acció enfront del VIH i altres ITS 2021-2030*) https://salutpublica.gencat.cat/web/.content/minisite/aspcat/vigilancia_salut_publica/vih-sida-its/Pla-dAccio-enfront-del-VIH-i-altres-ITS-2021-2030_ok.pdf | D1 | Action plan for the fight against HIV and other STI | Prevention, Testing, Treatment, Care | Gay men and other MSM, Sex workers, Transgender people, People who inject drugs, migrants (including unaccompanied minors), Incarcerated people, Young and adolescent people, women. | Direct | Adopted | Facilitates |
| Spain - Catalonia | Decree 142/2010, of 11 October, approving the 2010-2011 Social Services Portfolio. (*Decret 142/2010, d'11 d'octubre, pel qual s'aprova la Cartera de Serveis Socials 2010-2011.*) https://portaljuridic.gencat.cat/eli/es-ct/d/2010/10/11/142 | A12 | Decree approving the Social Services Portfolio. Section 1.2 | Care, Prevention | Socially vulnerable PLHIV and general population. | Direct | Adopted | Facilitates |
| Spain - Catalonia | Decree 21/2023, of February 7, on the organization of early childhood education (*Decret 21/2023, de 7 de febrer, d'ordenació dels ensenyaments de l'educació infantil*) https://cido.diba.cat/legislacio/14867688/decret-212023-de-7-de-febrer-dordenacio-dels-ensenyaments-de-leducacio-infantil-departament-deducacio | B8 | Decree establishing the arrangement of the teachings of the early childhood education stage | Prevention | Children | Indirect | Adopted | Facilitates |
| Spain - Catalonia | Elements for the implementation of pre-exposure prophylaxis to HIV (PrEP) in Catalonia (*Elements per a la implementació de la profilaxi pre-exposició al VIH (PrEP) a Catalunya*) https://salutpublica.gencat.cat/web/.content/minisite/aspcat/vigilancia_salut_publica/vih-sida-its/02_VIH-SIDA/guies/Elements-per-a-la-implementacio-de-la-PrEP-a-Catalunya.-Febrer_2022_accessible.pdf | B6 | Regional PrEP program | Prevention | Gay men and other MSM, Sex workers, Transgender people, People who inject drugs | Direct | Adopted | Facilitates |
| Spain - Catalonia | Functional plan of the syringe exchange program in community pharmacies. (*Pla funcional del programa d’intercanvi de xeringues a les farmàcies comunitàries*) https://scientiasalut.gencat.cat/bitstream/handle/11351/3575/pla_funcional_programa_intercanvi_xeringues_farmacies_comunitaries_2017.pdf?sequence=1&isAllowed=y | C5.2 | Syringe exchange program in Community Pharmacies | Prevention | People who inject drugs | Direct | Adopted | Facilitates |
| Spain - Catalonia | Functional Plan Syringe Exchange Program in Primary Care (*Pla Funcional Programa d’Intercanvi de Xeringues a l’Atenció Primària*) https://drogues.gencat.cat/web/.content/minisite/drogues/professionals/reduccio_danys/programes/pix/material/Pla-Funcional-PIX-Atencio-Primaria_ok_OKK.pdf | C5.1 | Syringe exchange program in Community Pharmacies | Prevention | People who inject drugs | Direct | Adopted | Facilitates |
| Spain - Catalonia | Health plan of Catalonia 2021-2025 (*Pla de salut de Catalunya 2021-2025*) https://scientiasalut.gencat.cat/bitstream/handle/11351/7948/pla_salut_catalunya_2021_2025_2021.pdf?sequence=1&isAllowed=y | B13 | Health Plan. Objective 14 | Prevention | General population | Direct | Adopted | Facilitates |
| Spain - Catalonia | Law 11/2014, of October 10, to guarantee the rights of lesbians, gays, bisexuals, transgender and intersex and to eradicate homophobia, biphobia and transphobia. (*Ley 11/2014, de 10 de octubre, para garantizar los derechos de lesbianas, gays, bisexuales, transgéneros e intersexuales y para erradicar la homofobia, la bifobia y la transfobia.*) https://www.boe.es/buscar/pdf/2014/BOE-A-2014-11990-consolidado.pdf | C8-B | Anti-discrimination law. Article 1 | Prevention, Testing, Treatment, Care | Gay men and other MSM, Transgender people | Indirect | Adopted | Facilitates |
| Spain - Catalonia |  | C8-D | Anti-discrimination law. Article 1 | Prevention, Testing, Treatment, Care | Transgender people | Indirect | Adopted | Facilitates |
| Spain - Catalonia | Law 17/2020, of December 22, amending Law 5/2008, on the right of women to eradicate male violence (Ley *17/2020, del 22 de desembre, de modificació de la Llei 5/2008, del dret de les dones a erradicar la violència masclista)* https://portaldogc.gencat.cat/utilsEADOP/PDF/8303/1828756.pdf | C13.2 | Law on the right of women to eradicate male violence. Articles 1, 3, 4, 19, 23, 27 | Prevention | Sex workers, Transgender people, Female migrants, women living with HIV, ethnic minority women. Women in general | Indirect | Adopted | Facilitates |
| Spain - Catalonia | Law 19/2020, of December 30, on equal treatment and non-discrimination. (*Ley 19/2020, de 30 de diciembre, de igualdad de trato y no discriminación.)* https://www.boe.es/buscar/act.php?id=BOE-A-2021-1663 | C8 | Anti-discrimination law. Article 1, Paragraph 3 | Prevention, Testing, Treatment, Care | Gay men and other MSM, Sex workers, Transgender people, Incarcerated people, migrants (including unaccompanied minors), PLHIV and population in general. | Indirect | Adopted | Facilitates |
| Spain - Catalonia |  | C8-E | Anti-discrimination law. Article 1, Paragraph 3 | Prevention, Testing, Treatment, Care | Migrants (including unaccompanied minors), | Indirect | Adopted | Facilitates |
| Spain - Catalonia |  | C8-F | Anti-discrimination law. Article 1, Paragraph 3 | Prevention, Testing, Treatment, Care | Incarcerated people | Indirect | Adopted | Facilitates |
| Spain - Catalonia |  | C8-A | Anti-discrimination law. Article 1, Paragraph 3 | Prevention, Testing, Treatment, Care | PLHIV | Indirect | Adopted | Facilitates |
| Spain - Catalonia | Law 5/2008, of April 24, on the right of women to eradicate male violence (*Llei 5/2008, del 24 d’abril, del dret de les dones a eradicar la violència masclista*) https://www.parlament.cat/document/nom/TL75.pdf | C13.1 | Law on the right of women to eradicate male violence. Chapter 5, Article 72 | Prevention | Sex workers, Transgender people, Female migrants, women living with HIV, ethnic minority women, etc. | Indirect | Adopted | Facilitates |
| Spain - Catalonia | Order of February 20, 1991, regulating the treatments contemplated by Royal Decree 75/1990, of January 19. (*Ordre de 20 de febrer de 1991, reguladora dels tractaments que contempla el Reial Decret 75/1990, de 19 de gener.*) https://portaljuridic.gencat.cat/ca/document-del-pjur/?documentId=66599 | B7 | Framework for treatment of opiate-dependent people. Articles 1 to 10 | Prevention | People who inject drugs, Other opiate-dependent persons | Direct | Adopted | Facilitates |
| Spain - Catalonia |  | C6 | Framework for treatment of opiate-dependent people. Articles 1 to 10 | Prevention | People who inject drugs | Indirect | Adopted | Facilitates |
| Spain - Catalonia | Recommendations for the prevention of vertical transmission of HIV (*Recomanacions per a la prevenció de la transmissió vertical del VIH*) https://salutpublica.gencat.cat/web/.content/minisite/aspcat/vigilancia_salut_publica/vih-sida-its/02_VIH-SIDA/protocols/llibret_optimitzat_vih.pdf | A11 | Guidelines on prevention of the vertical transmission of HIV. Sections 1 to 5 | Prevention, Testing, Treatment | Women living with HIV who are or want to become pregnant, and exposed newborns. | Direct | Adopted | Facilitates |
| Spain - Catalonia | Resolution SLT/3715/2022, of 18 November, by which the call for proposals for the year 2023 is opened, for the granting of subsidies for the financing of non-profit private entities, for the actions in the 'area of health for the prevention of HIV/AIDS infection (ref. BDNS 660473). *(Resolució SLT/3715/2022, de 18 de novembre, per la qual s'obre la convocatòria per a l'any 2023, per a la concessió de subvencions per al finançament a entitats privades sense afany de lucre, per a les actuacions en l'àmbit de la salut per a la prevenció de la infecció per VIH/sida (ref. BDNS 660473).*) https://dogc.gencat.cat/ca/document-del-dogc/?documentId=943500 | C14 | Resolution on the financing of non-profit private entities for the actions in the 'area of health for the prevention of HIV/AIDS infection | Prevention | Gay men and other MSM, Sex workers, Transgender people, People who inject drugs, Incarcerated people, migrants (including unaccompanied minors), Insert other national priority population 2: people who use drugs during sex, General population | Direct | Adopted | Facilitates |
| Sweden | Act (1972:119) on determination of gender in certain cases *(Lag (1972:119) om fastställande av könstillhörighet i vissa fall)* https://www.riksdagen.se/sv/dokument-lagar/dokument/svensk-forfattningssamling/lag-1972119-om-faststallande-av_sfs-1972-119#overgang | C9 | Act on determination of gender in certain cases | Prevention, Testing, Treatment, Care | Transgender people | Indirect | Adopted | Facilitates |
| Sweden | Act (2006:323) on the exchange of syringes and needles *(Lag (2006:323) om utbyte av sprutor och kanyler)* https://www.riksdagen.se/sv/dokument-lagar/dokument/svensk-forfattningssamling/lag-2006323-om-utbyte-av-sprutor-och-kanyler_sfs-2006-323 | B7 | Act on the exchange of syringes and needles | Prevention | People who inject drugs | Direct | Adopted | Facilitates |
| Sweden |  | C5 | Act on the exchange of syringes and needles | Prevention | People who inject drugs | Direct | Adopted | Facilitates |
| Sweden | Act (2009:253) to amend the marriage code (1987:230) *(Lag (2009:253) om ändring i äktenskapsbalken (1987:230))* https://www.icj.org/wp-content/uploads/2013/05/Sweden-Marriage-Code-1987-eng.pdf | C16 | Act to amend the marriage code | Prevention, Testing, Treatment, Care | Gay men and other MSM, Transgender people, Lesbians | Indirect | Adopted | Facilitates |
| Sweden | Act (2021:642) on the Institute for Human Rights *(Lag (2021:642) om Institutet för mänskliga rättigheter)* https://www.riksdagen.se/sv/dokument-lagar/dokument/svensk-forfattningssamling/lag-2021642-om-institutet-for-manskliga_sfs-2021-642 | C10 | Institute for Human Rights | Treatment, Care | General population | Indirect | Adopted | Facilitates |
| Sweden | Antiretroviral treatment of HIV infection - Recommendation 2021 *(Antiretroviral behandling av hivinfektion 2021 – Behandlingsrekommendation)* https://www.sls.se/globalassets/rav/rekommendationer/rav_hiv_2021_220318.pdf | A1 | HIV treatment guidelines. Section 3: Treatment, subsection 3.2 | Treatment, Prevention | PLHIV | Direct | Adopted | Facilitates |
| Sweden |  | A2 | HIV treatment guidelines. Section 3: Treatment, subsection 3.2 | Treatment, Prevention | General public | Direct | Adopted | Facilitates |
| Sweden |  | A3 | HIV treatment guidelines. Section 2 | Treatment, Prevention, Care | PLHIV | Direct | Adopted | Facilitates |
| Sweden |  | A5 | HIV treatment guidelines. Section 4: Monitoring, subsection 4.2 | Care, Prevention, Treatment | PLHIV | Direct | Adopted | Facilitates |
| Sweden |  | A6 | HIV treatment guidelines. Section 7 | Treatment | PLHIV | Direct | Adopted | Facilitates |
| Sweden |  | A10 | HIV treatment guidelines | Treatment, Prevention, Care | PLHIV | Direct | Adopted | Facilitates |
| Sweden |  | A11 | HIV treatment guidelines. Section 3: Treatment, subsection 3.3 | Treatment, Prevention, Care | PLHIV | Direct | Adopted | Facilitates |
| Sweden |  | D2.1 | HIV treatment guidelines. Section 4 | Prevention, Treatment, Care | PLHIV | Direct | Adopted | Facilitates |
| Sweden |  | D5 | HIV treatment guidelines. Section 2 | Prevention, Treatment | PLHIV | Direct | Adopted | Facilitates |
| Sweden | Budget proposal for 2023 (*Budgetpropositionen för 2023*) https://www.regeringen.se/contentassets/def2026cac0b4ef7acf4afeb988326ed/budgetpropositionen-for-2023-hela-dokumentet-prop.2022231-.pdf | D4 | Budget proposal. Expenditure area 9 | Prevention | General population, PLHIV | Direct | Adopted | Facilitates |
| Sweden | Criminal Code *(Brottsbalk)* (1962:700) https://www.riksdagen.se/sv/dokument-lagar/dokument/svensk-forfattningssamling/brottsbalk-1962700_sfs-1962-700#K6 | C1 | Criminal Code does not mention same-sex relations or sexual orientation as a criminal offense | Prevention, Testing, Treatment, Care | Gay men and other MSM | Indirect | Adopted | Facilitates |
| Sweden |  | C2 | Criminal Code. Chapter 6: Sex Crimes, Section 11 | Prevention, Testing, Treatment, Care | Sex workers | Indirect | Not adopted | Facilitates / Hinders |
| Sweden |  | C7.2 | Criminal Code. Chapter 3 | Prevention, Testing, Treatment, Care | PLHIV | Indirect | Partially adopted | Hinders |
| Sweden |  | C13 | Criminal Code. Chapter 4, Section 4 | Prevention, Testing, Treatment, Care | Sex workers, Transgender people, Women | Indirect | Adopted | Facilitates |
| Sweden | Discrimination Act *(Diskrimineringslag)* (2008:567)  https://www.riksdagen.se/sv/dokument-lagar/dokument/svensk-forfattningssamling/diskrimineringslag-2008567_sfs-2008-567 | C8.1 | Anti-discrimination Act. Chapter 1, Section 4 | Prevention, Testing, Treatment, Care | Gay men and other MSM, Transgender people, General population | Indirect | Adopted | Facilitates |
| Sweden |  | C8-B | Anti-discrimination Act. Chapter 1, Section 4 | Prevention, Testing, Treatment, Care | Gay men and other MSM | Indirect | Adopted | Facilitates |
| Sweden |  | C8-D | Anti-discrimination Act. Chapter 1, Section 4 | Prevention, Testing, Treatment, Care | Transgender people | Indirect | Adopted | Facilitates |
| Sweden |  | C8-E | Anti-discrimination Act. Chapter 1, Section 4 | Prevention, Testing, Treatment, Care | Migrants (People originating from high endemic areas), | Indirect | Adopted | Facilitates |
| Sweden | Follow-up time after HIV exposure: Guidance from the Public Health Authority and the Reference Group for Antiviral Therapy (RAV) *(Uppföljningstid efter hivexposition Vägledning från Folkhälsomyndigheten och Referensgruppen för antiviral terapi (RAV) https://www.sls.se/globalassets/rav/rekommendationer/uppfoljningstid-efter-hivexposition-150413.pdf* | B1 | Follow-up time and testing after HIV exposure guidelines | Prevention, Testing | General population | Direct | Adopted | Facilitates |
| Sweden |  | B2 | Follow-up time and testing after HIV exposure guidelines | Prevention, Testing | General population | Direct | Not adopted | Hinders |
| Sweden |  | B3.1 | Follow-up time and testing after HIV exposure guidelines | Prevention | PLHIV and their sexual partners | Direct | Not adopted | Hinders |
| Sweden | Good and equal health - a developed public health policy *(God och jämlik hälsa - en utvecklad folkhälsopolitik)* https://data.riksdagen.se/fil/7DFAC6CD-6BBC-476E-BB21-740C5A575867 | B13 | Public Health Policy. Sections 4, 6.1.1 and 6.1.2 | Prevention | Gay men and other MSM, Transgender people, Migrants (People originating from high endemic areas), General population | Direct | Adopted | Facilitates |
| Sweden | HIV pre-exposure prophylaxis (PrEP) – Recommendation on practical management 2023 *(Hiv preexpositionsprofylax (PrEP) – Rekommendation om praktisk handläggning 2023)* https://www.sls.se/globalassets/rav/rekommendationer/hiv-preexpositionsprofylax-prep-praktisk-handlaggning-2023-final.pdf | B6 | PrEP guidelines | Prevention | Gay men and other MSM, Transgender people | Direct | Adopted | Facilitates |
| Sweden | Infection Prevention Act *(Smittskyddslag)* (2004:168) https://www.riksdagen.se/sv/dokument-lagar/dokument/svensk-forfattningssamling/smittskyddslag-2004168_sfs-2004-168#K2 | B3.2 | Communicable Diseases Act. Chapters 2 and 3 | Prevention | PLHIV and their sexual partners | Direct | Partially adopted | Hinders |
| Sweden |  | C7.1 | Communicable Diseases Act. Chapters 2 and 4 | Prevention, Testing, Treatment, Care | PLHIV | Indirect | Partially adopted | Hinders |
| Sweden | Narcotics Penalty Act *(Narkotikastrafflag)* (1968:64) https://www.riksdagen.se/sv/dokument-lagar/dokument/svensk-forfattningssamling/narkotikastrafflag-196864_sfs-1968-64 | C3 | Narcotics Penalty Act. Section 1 | Prevention, Testing, Treatment, Care | People who inject drugs | Indirect | Not adopted | Hinders |
| Sweden | National guidelines for care and support in cases of addiction and addiction (*Nationella riktlinjer för vård och stöd vid missbruk och beroende*) https://www.socialstyrelsen.se/kunskapsstod-och-regler/regler-och-riktlinjer/nationella-riktlinjer/riktlinjer-och-utvarderingar/missbruk-och-beroende/ | C6 | Guidelines on treatment and support of individuals with substance abuse and addiction issues | Prevention | People who inject drugs | Direct | Adopted | Facilitates |
| Sweden | National strategy against HIV/AIDS and certain other infectious diseases *(Nationell strategi mot hiv/aids och vissa andra smittsamma sjukdomar*) https://www.regeringen.se/contentassets/31973c636b8d4caf966cc30d0273d367/nationell-strategi-mot-hiv-aids-och-vissa-andra-smittsamma-sjukdomar.pdf | B8 | National strategy against HIV/AIDS and certain other infectious diseases. Chapter 2, Section 2.3.5 | Prevention | Youth and young adults at risk, General population | Indirect | Adopted | Facilitates |
| Sweden |  | B9 | National strategy against HIV/AIDS and certain other infectious diseases. Chapter 2, Section 2.3.6 | Prevention | Sex workers | Direct | Adopted | Facilitates |
| Sweden |  | B10 | National strategy against HIV/AIDS and certain other infectious diseases. Chapter 2, Section 2.3.1 | Prevention | Gay men and other MSM | Direct | Adopted | Facilitates |
| Sweden |  | D1 | National strategy against HIV/AIDS and certain other infectious diseases | Prevention, Testing, Treatment, Care | Gay men and other MSM, Sex workers, Transgender people, People who inject drugs, Incarcerated people, Migrants (People originating from high endemic areas), Insert other national priority population 2: Youth and young adults at risk, | Direct | Adopted | Facilitates |
| Sweden |  | D2.2 | National strategy against HIV/AIDS and certain other infectious diseases | Prevention | Gay men and other MSM, Sex workers, Transgender people, People who inject drugs, Incarcerated people, Migrants (People originating from high endemic areas), Insert other national priority population 2: Youth and young adults at risk, | Direct | Adopted | Facilitates |
| Sweden |  | D6 | National strategy against HIV/AIDS and certain other infectious diseases. Section 2.5 | Prevention, Treatment, Testing, Care | PLHIV | Direct | Adopted | Facilitates |
| Sweden | National Strategy for Sexual and Reproductive Health and Rights (SRHR) *(Nationell strategi för sexuell och reproduktiv hälsa och rättigheter)* https://www.folkhalsomyndigheten.se/contentassets/0d489b0821164e949c03e6e2a3a7e6cc/nationell-strategi-sexuell-reproduktiv-halsa-rattigheter.pdf | B12 | National Strategy for Sexual and Reproductive Health and Rights | Prevention | Gay men and other MSM, Transgender people, Migrants (People originating from high endemic areas), Youth and young adults at risk, General population | Direct | Adopted | Facilitates |
| Sweden | Patents Act *(Patentlag)* (1967:837) https://www.riksdagen.se/sv/dokument-lagar/dokument/svensk-forfattningssamling/patentlag-1967837_sfs-1967-837#K6 | D7 | Patents Act. Chapter 6 |  | General population | Indirect | Partially adopted | Facilitates |
| Sweden | Recommendations for preventive measures against tuberculosis *(Rekommendationer för preventiva insatser mot tuberkulos)* https://www.folkhalsomyndigheten.se/contentassets/92e06754e3464636b1bdbb980378bcf3/rekommendationer-for-preventiva-insatser-mot-tuberkulos.pdf | A9 | Recommendations for preventive measures against tuberculosis. Section "People living with HIV" | Treatment | PLHIV | Direct | Adopted | Facilitates |
| Sweden | Schools Act *(Skollag)* (2010:800) https://www.riksdagen.se/sv/dokument-lagar/dokument/svensk-forfattningssamling/skollag-2010800_sfs-2010-800 | C12 | Schools Act. Chapter 1, Section 8 and Chapter 7 | Prevention | Youth and young adults at risk, General population | Indirect | Adopted | Facilitates |
| Sweden | The Instrument of Government *(Regeringsformen)* (1974:152) https://www.riksdagen.se/globalassets/07.-dokument--lagar/regeringsformen-eng-2021.pdf | C8 | Instrument of Government. Chapter 1, Article 2 and Chapter 2 | Prevention, Testing, Treatment, Care | Gay men and other MSM, Transgender people, General population | Indirect | Adopted | Facilitates |
| Sweden |  | C11 | Instrument of Government. Chapter 1, Article 2 | Prevention, Testing, Treatment, Care | General population | Indirect | Adopted | Facilitates |
| Switzerland | Federal Act on Controlling Communicable Human Diseases (*Bundesgesetz über die Bekämpfung übertragbarer Krankheiten des Menschen*) https://www.fedlex.admin.ch/eli/cc/2015/297/de | A8 | Epidemics Act | Prevention, Treatment | Incarcerated people | Indirect | Adopted | Facilitates |
| Switzerland |  | B11 | Epidemics Act | Prevention | Incarcerated people | Direct | Adopted | Facilitates |
| Switzerland | Federal Constitution of the Swiss Confederation (*Bundesverfassung der Schweizerischen Eidgenossenschaft*) https://www.fedlex.admin.ch/eli/cc/1999/404/de | C8 | The Constitution. Article 8 | Prevention, Testing, Treatment, Care | General population | Indirect | Adopted | Facilitates |
| Switzerland |  | C8-A | The Constitution. Article 8 | Prevention, Testing, Treatment, Care | PLHIV | Indirect | Adopted | Facilitates |
| Switzerland |  | C8-B | The Constitution. Article 8 | Prevention, Testing, Treatment, Care | Gay men and other MSM | Indirect | Adopted | Facilitates |
| Switzerland |  | C8-C | The Constitution. Article 8 | Prevention, Testing, Treatment, Care | People who inject drugs | Indirect | Adopted | Facilitates |
| Switzerland |  | C8-D | The Constitution. Article 8 | Prevention, Testing, Treatment, Care | Transgender people | Indirect | Adopted | Facilitates |
| Switzerland |  | C8-E | The Constitution. Article 8 | Prevention, Testing, Treatment, Care | Migrants | Indirect | Adopted | Facilitates |
| Switzerland |  | C8-F | The Constitution. Article 8 | Prevention, Testing, Treatment, Care | Incarcerated people | Indirect | Adopted | Facilitates |
| Switzerland |  | C11 | The Constitution. Article 41 | Prevention, Testing, Treatment, Care | General population, PLHIV | Indirect | Adopted | Facilitates |
| Switzerland | Federal law about narcotics and psychotropic substances (*Bundesgesetz über die Betäubungsmittel und die psychotropen Stoffe*) https://www.fedlex.admin.ch/eli/cc/1952/241_241_245/de | B7 | Law about narcotics and psychotropic substances. Chapter 1a, Article 3b | Prevention | People who inject drugs | Direct | Adopted | Facilitates |
| Switzerland |  | C3 | Law about narcotics and psychotropic substances. Chapter 2, Article 8 | Prevention, Testing, Treatment, Care | People who inject drugs | Indirect | Not adopted | Hinders |
| Switzerland | Federal law on health insurance (*Loi fédérale sur l’assurance-maladie*) https://www.fedlex.admin.ch/eli/cc/1995/1328_1328_1328/fr | A7 | Law on health insurance. Article 2 | Prevention, Testing, Treatment, Care | Migrants | Indirect | Partially adopted | Facilitates |
| Switzerland |  | D5 | Law on health insurance. Article 2 | Prevention, Testing, Treatment, Care | General population | Direct | Partially adopted | Facilitates / 2.Hinders |
| Switzerland |  | D6 | Law on health insurance. Article 2, Article 64 | Prevention, Testing, Treatment, Care | General population | Indirect | Not adopted | Hinders |
| Switzerland | Federal Law on Invention Patents (*Bundesgesetz über die Erfindungspatente)* https://www.fedlex.admin.ch/eli/cc/1955/871_893_899/de#tit_1/chap_5/lvl_H | D7 | Law on Invention Patents. Chapter III, Article 40d | Treatment |  | Indirect | Adopted | Facilitates |
| Switzerland | Federal Law on Measures for Civil Peacebuilding and the Strengthening of Human Rights (*Bundesgesetz über Massnahmen zur zivilen Friedensförderung und Stärkung der Menschenrechte*) https://www.fedlex.admin.ch/eli/cc/2004/253/de | C10 | Law on Measures for Civil Peacebuilding and the Strengthening of Human Rights. Section 3 | Treatment, Care | General population | Indirect | Adopted | Facilitates |
| Switzerland | Federal Narcotics Act Amendment of March 20, 1975 (*Bundesgesetz über die Betäubungsmittel Änderung vom 20. März 1975*) https://www.amtsdruckschriften.bar.admin.ch/viewOrigDoc/30001489.pdf?id=30001489 | C6 | Narcotics Act Amendment. Article 15a | Prevention | People who inject drugs | Direct | Adopted | Facilitates |
| Switzerland | Guidelines and recommendations on sexually transmitted infections: Recommendations of the Swiss Federal Commission for Sexual Health for Medical Care of HIV-Infected Women and their Offspring (*Empfehlungen der Eidgenössischen Kommission für sexuelle Gesundheit für medizinische Versorgung von HIV-infizierten Frauen und ihren Kindern*) https://www.bag.admin.ch/dam/bag/en/dokumente/mt/p-und-p/richtlinien-empfehlungen/fcsh-mtct-hiv.pdf.download.pdf/fcsh-mtct-hiv.pdf | A11 | Recommendations for Sexual Health for Medical Care of HIV-Infected Women and their Offspring. Sections 3.1, 3.2, 4.1 to 4.4 | Prevention, Treatment | Women living with HIV, Newborns exposed to/infected with HIV | Direct | Adopted | Facilitates |
| Switzerland | Guidelines for HIV testing as part of Voluntary Counselling and Testing (*Richtlinien zum HIV-Test im Rahmen von VCT*) https://www.bag.admin.ch/dam/bag/de/dokumente/mt/p-und-p/richtlinien-empfehlungen/vct-richtlinien.pdf.download.pdf/vct-richtlinien.pdf | B1 | HIV testing guidelines. Section 3.5 | Testing | Gay men and other MSM, Sex workers, People who inject drugs, Incarcerated people, General population | Direct | Adopted | Facilitates |
| Switzerland |  | B4 | HIV testing guidelines. Section 4.4 | Testing | General population | Direct | Adopted | Facilitates |
| Switzerland | National Programme on HIV and other STI (*Nationales Programm HIV und andere sexuell übertragbare Infektionen*) https://www.bag.admin.ch/bag/en/home/strategie-und-politik/nationale-gesundheitsstrategien/nationales-programm-hiv-und-andere-sexuell-uebertragbare-infektionen.html | D1 | National Programme on HIV and other STI | Prevention, Testing, Treatment, Care | Gay men and other MSM, People who inject drugs, Incarcerated people, General population, PLHIV and their partners, migrants | Direct | Adopted | Facilitates |
| Switzerland |  | D2 | National Programme on HIV and other STI. Section 14.3 |  | Gay men and other MSM, People who inject drugs, Incarcerated people, General population, PLHIV and their partners, migrants | Direct | Adopted | Facilitates |
| Switzerland | National Strategy on Addiction and Action Plan  https://www.bag.admin.ch/dam/bag/en/dokumente/npp/Sucht/Nationale%20Strategie%20und%20Massnahmenplan%20Sucht.pdf.download.pdf/Nationale%20Strategie%20und%20Massnahmenplan%20Sucht.pdf | C4 | National Strategy on Addiction | Prevention | People who inject drugs, All drug users | Indirect | Adopted | Facilitates |
| Switzerland | Ordinance on the electronic patient record (*Verordnung über das elektronische Patientendossier(VEP)) https://www.fedlex.admin.ch/eli/cc/2017/204/de* | D8 | Ordinance on the electronic patient record. Chapter 1 | Treatment | General population, PLHIV | Indirect | Partially adopted | Facilitates |
| Switzerland | Penal Code (*Strafgesetzbuch*) https://www.fedlex.admin.ch/eli/cc/54/757_781_799/de | C1 | The Penal Code does not state that same-sex sexual activities are forbidden or a crime | Prevention, Testing, Treatment, Care | Gay men and other MSM | Indirect | Adopted | Facilitates |
| Switzerland |  | C2 | The Penal Code does not state that same-sex sexual activities are forbidden or a crime. Articles 195 and 199 | Prevention, Testing, Treatment, Care | Sex workers | Indirect | Partially adopted | Facilitates |
| Switzerland |  | C7 | The Penal Code does not state that same-sex sexual activities are forbidden or a crime. Article 231 | Prevention, Testing, Treatment, Care | PLHIV | Indirect | Partially adopted | Hinders |
| Switzerland |  | C13 | The Penal Code does not state that same-sex sexual activities are forbidden or a crime. Article 123, 126, 180 | Prevention, Testing, Treatment, Care | General population | Indirect | Adopted | Facilitates |
| Switzerland | Recommendation of the Federal Commission for Sexual Health EKSG for monitoring the viral load in patients receiving HIV therapy (*Empfehlung der Eidgenössischen Kommission für sexuelle Gesundheit EKSG zum Monitoring der Viruslast bei Patientinnen und Patienten unter HIV Therapie*) https://www.bag.admin.ch/dam/bag/de/dokumente/mt/p-und-p/richtlinien-empfehlungen/empfehlung-eksg-monitoring-viruslast.pdf.download.pdf/empfehlung-eksg-monitoring-viruslast.pdf | A5 | Guidelines on monitoring the viral load in patients receiving HIV therapy. | Prevention, Treatment | PLHIV | Direct | Partially adopted | Facilitates |
| Switzerland | Recommendation of the Federal Commission for sexual health for the delivery of HIV tests for self-testing («HIV self-tests») to the public (*Empfehlung der Eidgenössischen Kommission für sexuelle Gesundheit zur Abgabe von HIV-Tests zur Eigenanwendung («HIV-Selbsttests») an das Publikum*) https://www.bag.admin.ch/dam/bag/de/dokumente/mt/p-und-p/richtlinien-empfehlungen/eksg-hiv-test-zur-eigenanwendung-2018.pdf.download.pdf/ | B2 | recommendations for the delivery of HIV tests for self-testing to the public. Section "Conclusions" | Testing | General population | Direct | Adopted | Facilitates |
| Switzerland | Recommendations of the specialist commission for clinics and therapy at the start of antiretroviral therapy in HIV-infected adults (*Empfehlungen der Fachkommission Klinik und Therapie zum Beginn der antiretroviralen Therapie bei HIV-infizierten Erwachsenen*) https://www.bag.admin.ch/dam/bag/de/dokumente/mt/p-und-p/archiv-richtlinien-empfehlungen/empfehlungen-der-fachkommission-klinik-und-therapie-zum-beginn-der-antiretroviralen-hiv-therapie.pdf.download.pdf/fkt-empfehlungen-start.pdf | A1 | Recommendation on starting antiretroviral therapy in HIV-infected adults. Section 2 | Prevention, Treatment | PLHIV | Direct | Not adopted | Hinders |
| Switzerland |  | A2 | Recommendation on starting antiretroviral therapy in HIV-infected adults. Section 3 | Prevention, Treatment | PLHIV and their sexual partners | Direct | Not adopted | Hinders |
| Switzerland | Recommendations of the Swiss Federal Commission for Sexual Health on pre-exposure prophylaxis (PrEP) for HIV prevention (*Empfehlungen der Eidgenössischen Kommission für sexuelle Gesundheit zur HIV PräExpositionsprophylaxe (PrEP) in der Schweiz*) https://www.bag.admin.ch/dam/bag/en/dokumente/mt/p-und-p/richtlinien-empfehlungen/prep-empfehlungen-der-eksg-januar-2016.pdf.download.pdf/fcsh-recommendations-prep-hiv.pdf | B6 | Recommendations on PrEP | Prevention | General population | Direct | Adopted | Facilitates |
| Switzerland | Swiss Civil Code - Marriage for all (*Schweizerisches Zivilgesetzbuch -Ehe für alle*) https://www.fedlex.admin.ch/eli/fga/2020/2687/de | C16 | Civil Code. Article 94 | Prevention | Gay men and other MSM, Transgender people, Lesbians | Indirect | Adopted | Facilitates |
| Switzerland | Swiss Civil Code (*Schweizerisches Zivilgesetzbuch*) https://www.fedlex.admin.ch/eli/cc/24/233_245_233/en | C14 | Civil Code. Articles 60 and 81 | Prevention, Testing, Care | General population | Indirect | Adopted | Facilitates |
| Switzerland | Swiss Civil Code- Change of gender in civil status register (*Schweizerisches Zivilgesetzbuch- Änderung des Geschlechts im Personenstandsregister*) https://www.fedlex.admin.ch/eli/fga/2020/2689/de | C9 | Civil Code. Article 30b | Prevention, Testing, Treatment, Care | Transgender people | Indirect | Adopted | Facilitates |
| Switzerland | The Federal Council’s health policy strategy 2020–2030 (*Gesundheitspolitische Strategie des Bundesrats 2020–2030*) https://www.bag.admin.ch/dam/bag/en/dokumente/nat-gesundheitsstrategien/gesundheit-2030/strategie-gesundheit2030.pdf.download.pdf/strategie-gesundheit2030.pdf | B13 | Health policy strategy |  | General population | Indirect | Partially adopted | N/A |
| Switzerland | Urgent Action Plan https://www.bag.admin.ch/bag/en/home/strategie-und-politik/nationale-gesundheitsstrategien/nationales-programm-hiv-und-andere-sexuell-uebertragbare-infektionen/zielgruppe-mit-erhoehtem-expositionsrisiko-achse2/schwule-gesundheit-msm.html | B10 | Urgent Action Plan. Fields of action I, II and III | Prevention, Testing, Treatment | Gay men and other MSM | Direct | Adopted | Facilitates |
| Switzerland (Vaud) | Global, coordinated and multidisciplinary action plan against street trafficking and drug addiction (*Plan d'action global, coordonné et pluridisciplinaire face au trafic de rue et à l'addiction aux stupéfiants*) https://www.vd.ch/fileadmin/user_upload/organisation/dsas/DGS/fichiers_pdf/EMPD_politique_cantonale_Deal_de_Rue.pdf | B7 | Action plan against street trafficking and drug addiction. Sections 8.2.1 and C-Pillar | Prevention | People who inject drugs | Direct | Adopted | Facilitates |
| Switzerland (Vaud) |  | C4 | Action plan against street trafficking and drug addiction. | Prevention | People who inject drugs | Direct | Adopted | Facilitates |
| Switzerland (Vaud) |  | C5 | Action plan against street trafficking and drug addiction. C-Pillar | Prevention | People who inject drugs | Direct | Adopted | Facilitates |
| Switzerland (Vaud) | HIV-STI prevention skills center (*Centre de compétences prévention VIH-IST*) https://www.vd.ch/toutes-les-actualites/news/1776i-profa-developpe-un-centre-de-competences-prevention-vih-ist | A12 | HIV-STI prevention skills center | Prevention, Testing | Gay men and other MSM, General population | Direct | Adopted | Facilitates |
| Switzerland (Vaud) | Law on the organization of the prevention and fight against domestic violence (*Loi d'organisation de la prévention et de la lutte contre la violence domestique*) https://www.vd.ch/fileadmin/user_upload/organisation/dec/befh/PUBLICATIONS_-_REFONTE/violence_domestique/LOVD_futur.pdf | C13 | Law on the prevention and fight against domestic violence | Prevention | General population | Indirect | Adopted | Facilitates |
| Switzerland (Vaud) | Narcotics Regulation (*Règlement sur les stupéfiants*) https://prestations.vd.ch/pub/blv-publicatioctes/consolide/812.11.1?id=f8a89938-e188-4b94-a58b-5ee6915debb0 | C6 | Narcotics Regulation. Article 7 | Prevention | People who inject drugs | Direct | Adopted | Facilitates |
| Switzerland (Vaud) | Prison Medicine and Psychiatry Service (*Service de médecine et psychiatrie pénitentiaires*) https://www.chuv.ch/fr/fiches-psy/service-de-medecine-et-psychiatrie-penitentiaires-smpp | A8 | Prison Medicine and Psychiatry Service | Prevention, Treatment | Incarcerated people | Indirect | Adopted | Facilitates |
| Switzerland (Vaud) | Regulations implementing the law of 30 March 2004 on the practice of prostitution (*Règlement d'application de la loi du 30 mars 2004 sur l'exercice de la prostitution*) Available from: https://www.vd.ch/themes/economie/police-cantonale-du-commerce/informations-relatives-a-lexercice-de-la-prostitution | C2 | Regulations on the practice of prostitution | Prevention | Sex workers | Indirect | Adopted | Facilitates |
| Switzerland (Vaud) | School-based sex education (*Education sexuelle en milieu scolaire*) https://www.vd.ch/themes/formation/sante-a-lecole/prestations/sante-sexuelle | B8 | School-based sex education | Prevention | Students between 13 and 15 years old | Indirect | Adopted | Facilitates |
| United Kingdom | A guide to – Partner Notification https://www.bashhguidelines.org/media/1035/pn_pil_digital_2016.pdf | B3.2 | Partner notification guidelines | Prevention, Testing | PLHIV and their sexual partners | Direct | Adopted | Facilitates |
| United Kingdom | Advancing our health: prevention in the 2020s – consultation document. https://www.gov.uk/government/consultations/advancing-our-health-prevention-in-the-2020s/advancing-our-health-prevention-in-the-2020s-consultation-document | B13 | Consultation document on improving public health and prevention strategies. Section "Sexual and reproductive Health" | Prevention, Testing | General population | Indirect | Adopted | Facilitates |
| United Kingdom | BHIVA guidelines for the routine investigation and monitoring of adult HIV-1-positive individuals. (2019 interim update) https://www.bhiva.org/file/DqZbRxfzlYtLg/Monitoring-Guidelines.pdf | A5 | Guidelines for the routine investigation and monitoring of adult HIV-1-positive individuals | Treatment | PLHIV | Direct | Adopted | Facilitates |
| United Kingdom | BHIVA guidelines on antiretroviral treatment for Adults living with HIV-1 2022 ttps://www.bhiva.org/file/63513a1745ea9/BHIVA-guidelines-on-antiretroviral-treatment-for-adults-living-with-HIV-1-2022.pdf | A2 | Guidelines on antiretroviral treatment for Adults living with HIV-1 | Treatment, Prevention | PLHIV and their sexual partners | Direct | Not adopted | Hinders |
| United Kingdom |  | A3 | Guidelines on antiretroviral treatment for Adults living with HIV-1. Part 5 | Treatment, Prevention, Care | PLHIV and their sexual partners | Direct | Adopted | Facilitates |
| United Kingdom |  | A10 | Guidelines on antiretroviral treatment for Adults living with HIV-1 | Treatment, Care | Transgender people, PLHIV | Direct | Adopted | Facilitates |
| United Kingdom |  | A1 | Guidelines on antiretroviral treatment for Adults living with HIV-1 | Prevention, Treatment | PLHIV and their sexual partners | Direct | Adopted | Facilitates |
| United Kingdom | BHIVA/BASHH guidelines on the use of HIV pre-exposure prophylaxis (PrEP) 2018 https://www.bhiva.org/file/5b729cd592060/2018-PrEP-Guidelines.pdf | B6.2 | PrEP guidelines | Prevention | Gay men and other MSM, Transgender people, Sex workers, all individuals at risk - heterosexual men and women. | Direct | Adopted | Facilitates |
| United Kingdom | British HIV Association guidelines for the management of HIV in pregnancy and postpartum 2018 (2020 third interim update) https://www.bhiva.org/file/5f1aab1ab9aba/BHIVA-Pregnancy-guidelines-2020-3rd-interim-update.pdf | A11 | Guidelines for the management of HIV in pregnancy and postpartum | Testing, Treatment, Care, Prevention | Pregnant WLHIV and newborns | Direct | Adopted | Facilitates |
| United Kingdom | British HIV Association guidelines for the management of tuberculosis in adults living with HIV 2018 (2023 interim update) https://www.bhiva.org/TB-guidelines | A9 | Guidelines for the management of tuberculosis in adults living with HIV | Treatment, Care | PLHIV and TB | Direct | Adopted | Facilitates |
| United Kingdom | British HIV Association/British Association for Sexual Health and HIV/British Infection Association adult HIV testing guidelines 2020 https://www.bashhguidelines.org/media/1250/hiv-testing-2020-wiley.pdf | B2 | HIV testing guidelines. Section 5 | Prevention, Testing | Gay men and other MSM, Sex workers, Transgender people, People who inject drugs, Incarcerated people, Migrants, Black Africans, At risk groups | Direct | Adopted | Facilitates |
| United Kingdom |  | B1.2 | HIV testing guidelines | Testing, Prevention | Gay men and other MSM, Sex workers, Transgender people, People who inject drugs, Incarcerated people, Migrants, Black Africans, Attendees at sexual health clinics, people from high prevalence countries (migrants) | Direct | Adopted | Facilitates |
| United Kingdom | Charities Act, 2011 https://www.legislation.gov.uk/ukpga/2011/25/contents | C14 | Charities Act. Part 4 | Prevention, Testing, Care | General population | Indirect | Adopted | Facilitates |
| United Kingdom | Data Protection Act https://www.legislation.gov.uk/ukpga/2018/12/contents/enacted | D8 | Data Protection Act. Part 1, Section 2 | Treatment | General population | Indirect | Adopted | Facilitates |
| United Kingdom | Disclosing information about serious communicable diseases. https://www.gmc-uk.org/ethical-guidance/ethical-guidance-for-doctors/confidentiality---disclosing-information-about-serious-communicable-diseases/disclosing-information-about-serious-communicable-diseases | B3.1 | Guidelines on disclosing information about serious communicable diseases | Prevention, Testing | PLHIV and their sexual partners | Direct | Adopted | Facilitates |
| United Kingdom | Drug misuse in over 16s: opioid detoxification Clinical guideline [CG52] https://www.nice.org.uk/guidance/cg52 | C6 | Guideline on helping people who are dependent on opioids to stop using drugs. | Prevention | People who inject drugs | Direct | Adopted | Facilitates |
| United Kingdom | Equality Act 2010 https://www.legislation.gov.uk/ukpga/2010/15/contents | C8 | Equality Act. Part 2, Chapter 1 and Part 2, Chapter 2, Sections 13 and 19 | Prevention, Testing, Treatment, Care | General Population with Protected Characteristics | Indirect | Adopted | Facilitates |
| United Kingdom |  | C8-A | Equality Act. Part 2, Chapter 1, Section 6 | Prevention, Testing, Treatment, Care | PLHIV | Indirect | Adopted | Facilitates |
| United Kingdom |  | C8-B | Equality Act. Part 2, Chapter 1, Section 12 | Prevention, Testing, Treatment, Care | Gay men and other MSM | Indirect | Adopted | Facilitates |
| United Kingdom |  | C8-D | Equality Act. Part 2, Chapter 1, Section 7 | Prevention, Testing, Treatment, Care | Transgender people | Indirect | Adopted | Facilitates |
| United Kingdom |  | C8-E | Equality Act. Part 2, Chapter 1, Section 9 | Prevention, Testing, Treatment, Care | Migrants | Indirect | Adopted | Facilitates |
| United Kingdom | Equality and Human Rights Commission https://www.equalityhumanrights.com/en | C10 | Equality and Human Rights Commission | Treatment, Care | General population | Indirect | Adopted | Facilitates |
| United Kingdom | From harm to hope: A 10-year drugs plan to cut crime and save lives https://www.gov.uk/government/publications/from-harm-to-hope-a-10-year-drugs-plan-to-cut-crime-and-save-lives/from-harm-to-hope-a-10-year-drugs-plan-to-cut-crime-and-save-lives#chapter-3--delivering-a-world-class-treatment-and-recovery-system | C4 | Drug Strategy to combat illegal drug use and deal with addictions. Chapter 3 | Prevention | People who inject drugs, All drug users | Indirect | Adopted | Facilitates |
| United Kingdom | Gender Recognition Act 2004 https://www.legislation.gov.uk/ukpga/2004/7 | C9 | Gender Recognition Act. Article 1 | Prevention, Testing, Treatment, Care | Transgender people | Indirect | Adopted | Facilitates |
| United Kingdom | Guidance. NHS entitlements: migrant health guide https://www.gov.uk/guidance/nhs-entitlements-migrant-health-guide | A7 | Migrant health guidelines | Testing, Prevention, Treatment, Care | Migrants, PLHIV | Direct | Adopted | Facilitates |
| United Kingdom | HIV testing: encouraging uptake https://www.nice.org.uk/guidance/qs157/resources/hiv-testing-encouraging-uptake-pdf-75545545013701 | B1.1 | HIV testing guidelines | Testing, Prevention | Migrants, Gay men and other MSM, Transgender people, People who inject drugs, People in high and very high prevalence areas, people who practice chemsex, female sexual partners of MSM, people who are diagnosed or tested for an STI | Direct | Adopted | Facilitates |
| United Kingdom | HIV: surveillance, data and management https://www.gov.uk/government/statistics/hiv-annual-data-tables | D9 | HIV surveillance, data and management |  | All PLHIV | Direct | Partially adopted | Facilitates |
| United Kingdom | Misuse of Drugs Act 1971 https://www.legislation.gov.uk/ukpga/1971/38/contents | C3 | Misuse of Drugs Act. | Prevention, Testing, Treatment, Care | People who inject drugs | Indirect | Not adopted | Hinders |
| United Kingdom | National Health Service Act, 1946 https://www.legislation.gov.uk/ukpga/1946/81/pdfs/ukpga_19460081_en.pdf | D5 | National Health Service Act | Prevention, Testing, Treatment, Care | General population | Indirect | Adopted | Facilitates |
| United Kingdom |  | D6 | National Health Service Act | Prevention, Testing, Treatment, Care | General population | Indirect | Adopted | Facilitates |
| United Kingdom | Needle and syringe programs Public health guideline [PH52] https://www.nice.org.uk/guidance/ph52 | B7 | Needle and syringe programs | Prevention | People who inject drugs | Direct | Adopted | Facilitates |
| United Kingdom |  | C5 | Needle and syringe programs | Prevention | People who inject drugs | Direct | Adopted | Facilitates |
| United Kingdom | Offences Against the Person Act 1861 https://www.legislation.gov.uk/ukpga/Vict/24-25/100/contents | C7 | Offences Against the Person Act. Sections 18 and 20 | Prevention, Testing, Treatment, Care | PLHIV | Indirect | Partially adopted | Hinders |
| United Kingdom | Patents Act 1977 https://www.legislation.gov.uk/ukpga/1977/37/contents | D7 | Patents Act. Sections 48 to 54 | Treatment, Prevention | General population | Indirect | Adopted | Facilitates |
| United Kingdom | Policing and Crime Act 2009 https://www.legislation.gov.uk/ukpga/2009/26/contents | C2.1 | Policing and Crime Act. Part 2 | Prevention, Testing, Treatment, Care | Sex workers | Indirect | Partially adopted | Facilitates |
| United Kingdom | Prison Life. Healthcare in Prison https://www.gov.uk/life-in-prison/healthcare-in-prison | A8 | Healthcare in Prison | Prevention, Testing, Treatment, Care | Incarcerated people | Indirect | Adopted | Facilitates |
| United Kingdom | Reducing sexually transmitted infections NICE guideline [NG221] https://www.nice.org.uk/guidance/NG221 | B3.3 | Reducing sexually transmitted infections guidelines. Section 3 | Prevention, Testing | PLHIV and their sexual partners | Direct | Adopted | Facilitates |
| United Kingdom |  | B6.1 | Reducing sexually transmitted infections guidelines. Section 1 | Prevention | Gay men and other MSM, Sex workers, Transgender people, People who inject drugs, Incarcerated people, Migrants, Black Africans, | Direct | Adopted | Facilitates |
| United Kingdom | Sexual Offences Act 1967 https://www.legislation.gov.uk/ukpga/1967/60/pdfs/ukpga_19670060_en.pdf | C1 | Sexual Offences Act. Article 1 | Prevention, Testing, Treatment, Care | Gay men and other MSM | Indirect | Adopted | Facilitates |
| United Kingdom | Sexual Offences Act 2003 https://www.legislation.gov.uk/ukpga/2003/42/contents | C2.2 | The Sexual Offences Act. Articles 51A to 56 | Prevention, Testing, Treatment, Care | Sex workers | Indirect | Partially adopted | Facilitates |
| United Kingdom (England) | Black and minority ethnic men who have sex with men (BME MSM project) https://assets.publishing.service.gov.uk/government/uploads/system/uploads/attachment_data/file/525843/BlackandminorityethnicmenwhohavesexwithmenProjectevaluationandsystematicreview.pdf | B10 | Black and minority ethnic men who have sex with men initiative | Prevention | Gay men and other MSM, Black Africans | Indirect | Adopted | Facilitates |
| United Kingdom (England) | Condom Distribution Schemes in England 2015/16 https://assets.publishing.service.gov.uk/media/5a82bc84e5274a2e8ab59163/Condom_Distribution_Schemes_in_England_2015.pdf | B14 | Condom Distribution Schemes | Prevention | Gay men and other MSM, Sex workers, People who inject drugs, Young people up to 24 | Direct | Adopted | Facilitates |
| United Kingdom (England) | Education Act 1996 https://www.legislation.gov.uk/ukpga/1996/56/part/I/chapter/I/crossheading/compulsory-education | C12 | Education Act. Article 8 | Prevention | Young people | Indirect | Adopted | Facilitates |
| United Kingdom (England) | HIV monitoring and evaluation framework https://www.gov.uk/government/publications/hiv-monitoring-and-evaluation-framework | D2 | HIV monitoring and evaluation framework | Prevention, Testing, Treatment, Care | General population; all PLHIV | Direct | Adopted | Facilitates |
| United Kingdom (England) | HIV Testing and Self-Testing Information update November 2015 https://assets.publishing.service.gov.uk/government/uploads/system/uploads/attachment_data/file/769460/HIV_Self-Testing_PHE_Position_v13_-_Nov_15_updated.pdf | B2 | HIV Testing and Self-Testing Information update | Prevention, Testing | Gay men and other MSM, Migrants, Black African men and women | Direct | Adopted | Facilitates |
| United Kingdom (England) | Marriage (Same Sex Couples) Act 2013 https://www.legislation.gov.uk/ukpga/2013/30/enacted | C16 | Marriage Act. Article 1 | Prevention | Gay men and other MSM | Indirect | Adopted | Facilitates |
| United Kingdom (England) | PHE HIV and STI data sharing policy https://assets.publishing.service.gov.uk/government/uploads/system/uploads/attachment_data/file/423828/20150424_PHE_HIV_STI_Data_Sharing_Policy_v4.1.pdf | D9 | HIV and STI data sharing policy | Testing, Treatment, Care | PLHIV and their sexual partners | Direct | Partially adopted | Facilitates |
| United Kingdom (England) | Statutory guidance. Relationships and sex education (RSE) and health education https://assets.publishing.service.gov.uk/government/uploads/system/uploads/attachment_data/file/1090195/Relationships_Education_RSE_and_Health_Education.pdf | B8 | Guidance on relationships and sex education and health education | Prevention | Primary and secondary school children | Indirect | Adopted | Facilitates |
| United Kingdom (England) | The Safe Project https://www.nhs.uk/services/service-directory/the-safe-project/N10509825 | B9 | SAFE project | Prevention, Testing | Sex workers | Direct | Adopted | Facilitates |
| United Kingdom (England) | Towards Zero - An action plan towards ending HIV transmission, AIDS and HIV-related deaths in England - 2022 to 2025 https://www.gov.uk/government/publications/towards-zero-the-hiv-action-plan-for-england-2022-to-2025/towards-zero-an-action-plan-towards-ending-hiv-transmission-aids-and-hiv-related-deaths-in-england-2022-to-2025 | A12 | Action plan towards ending HIV transmission, AIDS and HIV-related deaths | Care | PLHIV | Direct | Adopted | Facilitates |
| United Kingdom (England) |  | D1 | Action plan towards ending HIV transmission, AIDS and HIV-related deaths | Prevention, Testing, Treatment, Care | Black Africans, General population | Direct | Adopted | Facilitates |
| United Kingdom (England) |  | B11 | Action plan towards ending HIV transmission, AIDS and HIV-related deaths | Prevention, Testing | Incarcerated people | Direct | Adopted | Facilitates |
| United Kingdom (England) | Violence against Women and Girls Guidance https://www.cps.gov.uk/legal-guidance/violence-against-women-and-girls-guidance | C13 | Violence against Women and Girls Guidance | Prevention | General population | Indirect | Adopted | Facilitates |

# Appendix V. Results from peer-reviewed literature search

## Table S5. Studies included in the analysis

| **Study** | **Article** | **Overview of findings about implementation of policy and its barriers** |
| --- | --- | --- |
| Schweikardt and Coppieters, 2015 [12] | The development of a national HIV Plan in Belgium: Achieving consensus despite institutional complexity | National HIV Plan- Financial issues to address screening, treatment, and care |
| Mravčík et al., 2017 [13] | HIV epidemic among men who have sex with men in the Czech Republic, 2016: high time for targeted action | National program for tackling HIV/AIDS- Neither MSM group nor activities targeting sex between men are defined as priorities, with no single specific activity focusing on MSM in the program. Budget constraints. |
| Rüütel et al., 2018 [14] | Monitoring HIV-indicator condition guided HIV testing in Estonia | Testing guidelines- Guidelines are not followed. Need for educating healthcare providers |
| Kontautaite et al., 2018 [15] | Study of human rights violations faced by women who use drugs in Estonia | Criminalization of drugs- Random drug testing and disclosure of private medical information violates rights and becomes an obstacle to testing and treatment for HIV |
| Ousseine et al., 2022 [16] | Pre-exposure prophylaxis in France: How many MSM are eligible and how much will it cost? | PrEP availability- Low uptake among eligible individuals due to structural, cultural, and stigma-related factors |
| Jacomet et al., 2020 [17] | Pharmacist's role in HIV care in France. Implication for clinical improvement of people living with HIV worldwide | Dispensing ARV in Pharmacies- No remuneration in place for the pharmacist's services to PLHIV even though ARV regimen can be complex |
| Annequin et al., 2020 [18] | Are PrEP services in France reaching all those exposed to HIV who want to take PrEP? MSM respondents who are eligible but not using PrEP (EMIS 2017) | Availability and reimbursement of PrEP- Need for a targeted and adapted response as it does not reach certain MSM subpopulations |
| Houborg and Jauffret-Roustide, 2022 [19] | Drug Consumption Rooms: Welfare State and Diversity in Social Acceptance in Denmark and in France | Legal Drug Consumption Rooms- Centralized government was not convinced that existing scientific literature was sufficient to implement Drug Consumption Rooms as a permanent measure |
| Dichtl et al., 2023 [20] | [HIV/HCV Among Drug Users in Germany: Elimination Targets in the Mirror of Practice] | Strategy to stop HIV, hepatitis B and C and other STIs among PWID- Strategy hindered by high workload, low financial resources for drug help, and the strict separation of medicine and social work |
| Seals, 2015 [21] | Worker rights and health protection for prostitutes: a comparison of The Netherlands, Germany, and Nevada | Worker rights and health protection for prostitutes- Germany: different types of prostitution complicate policy goals as these are locally determined. The desire to remain anonymous shows that prostitutes still face stigma and discrimination even after the legal change in their status. Netherlands: The desire to remain anonymous shows that prostitutes still face stigma and discrimination even after the legal change in their status. Prostitutes’ wish for anonymity and refusal to assert worker rights |
| Scholten et al., 2018 [22] | To prescribe, or not to prescribe: decision making in HIV-1 post-exposure prophylaxis | PEP guidelines- PEP prescription being influenced by emotional aspects, and not according to risk assessment. |
| Werner et al., 2018 [23] | Knowledge and use of HIV pre-exposure prophylaxis among men who have sex with men in Berlin - A multicentre, cross-sectional survey | PrEP availability- Low level of information. Too narrow guidelines. |
| Galli et al., 2020 [24] | HIV policy in Italy and recommendations across the HIV care continuum | National HIV Plan- Lack of dedicated funding to enable its implementation, lack of continuity, insufficient sex education, variations regarding clinical treatment |
| Andreoni et al., 2020 [25] | Beyond Italian guidelines in the management of HIV-positive patient | ART guidelines- Attitude and interpretation of recommendations by individual health professionals may differ across the country according to professional backgrounds and cultural attitudes. |
| Sander et al., 2016 [26] | Overview of harm reduction in prisons in seven European countries | Harm reduction in prisons- Spain: Due to serious opposition from the prison workers’ union for many years, NSPs did not become available in Catalan prisons until much later. Italy: Regionalization of the health service has led to discrepancies in service provision and quality. Latvia: The provision of free condoms is considered insufficient, there is no NSP provision, no medical professionals qualified to provide OST (methadone) within prisons. Poland: For prisoners, the treatment is not holistic. There is an apparent lack of HIV and HCV prevention, treatment and care and no real provision of social support. No evaluation on the effectiveness of OST in prisons. Portugal: Prisoners’ feared being discriminated against by, and suffering reprisals at the hands of, prison authorities for their participation in the NSP. Long waiting times and other difficulties in accessing OST while in prisons |
| Tibaldi et al., 2019 [27] | Vaginal delivery in women with HIV in Italy: results of 5 years of implementation of the national SIGO-HIV protocol | Vaginal delivery in HIV- Rate of vaginal delivery remains relatively low compared to other countries |
| Ferrara and Gianfredi, 2022 [28] | The Italian PrEPventHIV challenge: a scoping systematic review on HIV pre-exposure prophylaxis monitoring in Italy | PrEP implementation and monitoring- The need for informative and educative approaches that would specifically target potential user populations and healthcare professionals. Concern over the PrEP accessibility and affordability. |
| Goletti et al., 2020 [29] | Latent tuberculosis infection screening in persons newly-diagnosed with HIV infection in Italy: A multicentre study promoted by the Italian Society of Infectious and Tropical Diseases | Guidelines for latent tuberculosis infection among PLHIV- Low implementation of LTBI screening due to the low perception of TB risk in PLWH, and to the awareness of the inaccuracy of LTBI tests |
| Toxopeus et al., 2019 [30] | Compliance with laboratory monitoring guidelines in outpatient HIV care: a qualitative study in the Netherlands | HIV treatment guidelines- Unclear visual representation of the guidelines on the website, lack of supportive clinical management software, compartmentalization of the healthcare system, and assessment of risk behavior differing per patient. |
| Evenblij et al., 2016 [31] | Intention of physicians to implement guidelines for screening and treatment of latent tuberculosis infection in HIV-infected patients in The Netherlands: a mixed-method design | HIV-TB guideline- Disconnect between clinical practice and evidence-based recommendations in the guideline. Absence of direct experience with TB by physicians |
| Tokar et al., 2020 [32] | I don't want anyone to know': Experiences of obtaining access to HIV testing by Eastern European, non-European Union sex workers in Amsterdam, the Netherlands | Non-criminalization of sex work- Fragmented knowledge about whether and how ART treatment would be covered for non-EU female sex workers (non-EU female sex workers are not eligible to apply for a work permit to be employed as sex workers). |
| Malinowska-Sempruch et al., 2016 [33] | Shaping drug policy in Poland | Criminalization of drugs- No efforts to track the prevalence of HIV infection. Lack of individual and institutional leadership in the areas of HIV and drugs. |
| Balayan et al., 2021 [34] | Where are we with pre-exposure prophylaxis use in Central and Eastern Europe? Data from the Euroguidelines in Central and Eastern Europe (ECEE) Network Group | PrEP availability: Romania, Poland, and Estonia had PrEP available, but costs were to be fully covered by patients. |
| Meireles et al., 2020 [35] | Different guidelines for pre-exposure prophylaxis (PrEP) eligibility estimate HIV risk differently: an incidence study in a cohort of HIV-negative men who have sex with men, Portugal, 2014-2018 | PrEP availability- Different guidelines hold the potential of missing people in need of PrEP. |
| Sacramento, 2016 [36] | [Individuals, structures, and risks: an overview of primary HIV prevention in Portugal] | HIV program- Ideological blockages and/or the lack of political will. |
| Castellá et al., 2022 [37] | Approaches to STI Prevention and Control in a Highly Decentralized State: An Integrative Review | Plan for Prevention and Control of HIV- Major differences depending on the region. Poor alignment with the National Strategic Plan in some areas due to structural factors, associated with health care planning in each region, underinvestment in health care resources. |
| Gogishvili et al., 2021 [38] | Policy implementation analysis on access to healthcare among undocumented immigrants in seven autonomous communities of Spain, 2012-2018 | Universal healthcare system- Level of restriction varied across ACs. Varying emphasis of health as a human right versus disease containment perspective. |
| García et al., 2019 [39] | HIV pre-exposure prophylaxis (PrEP) in Spain: political and administrative situation | PrEP availability- Little political activity related to the promotion of PrEP availability. |
| Gogishvili et al., 2021 [40] | A qualitative study on mixed experiences of discrimination and healthcare access among HIV-positive immigrants in Spain | Universal healthcare system- NGOs as important safety net system against restrictions imposed by law. |
| Iniesta et al., 2022 [41] | Would eligible gay, bisexual and other men who have sex with men use PrEP? Awareness, knowledge, eligibility and intention to use PrEP among EMIS-2017 participants in Spain | PrEP availability- Limited awareness and low knowledge of PrEP. |
| Suárez-García et al., 2019 [42] | Reasons for noncompliance with the national guidelines for initial antiretroviral therapy of HIV-infected patients in Spain, 2010-2015 | HIV treatment guidelines- Inequalities in the access to ART, dependent on the patient’s place of residence and reference center. |
| Hoyos et al., 2021 [43] | Opinions Towards Key Operational Aspects for the Implementation of HIV Self-Testing in Spain: A Comparison between Stakeholders and Potential Users | HIV self-testing- Stakeholders being less supportive of self-testing. |
| Koerting et al., 2019 [44] | [The Social Pact for the Non-discrimination and Equal Treatment Associated with HIV development] | Non-Discrimination associated with HIV- Alliances and synergies with different sectors are needed for implementation. |
| Viciana-Fernández et al., 2015 [45] | [Barriers to ART initiation in HIV infected subjects and with treatment indication in Spain. Why don't they start their treatment? Bridgap Study] | ART guidelines- Even with free access, barriers related to the availability of a universal and affordable healthcare system. Physicians do not consider the indicator to start ART as absolute. Patients want to avoid toxicities, consider that ART is not useful, or cultural beliefs that prevent from starting it. |
| Herder and Agardh, 2019 [46] | Navigating between rules and reality: a qualitative study of HIV positive MSM's experiences of communication at HIV clinics in Sweden about the rules of conduct and infectiousness | HIV infection criminalization- Rules were not always clearly communicated to patients. |
| Amroussia et al., 2022 [47] | Migrants in Swedish sexual and reproductive health and rights related policies: a critical discourse analysis | National Sexual and Reproductive Health and Rights strategy- Changing focus from risk identification to prioritizing prevention and reducing vulnerability. |
| Karlsson et al., 2021 [48] | Could 30 years of political controversy on needle exchange programmes in Sweden contribute to scaling-up harm reduction services in the world? | Needle Exchange Program- NEP law passed, with municipality veto in order for more NEP to start. |
| Barrett et al., 2022 [49] | Best interests and low thresholds: legal and ethical issues relating to needle and syringe services for under 18s in Sweden | Needle and Syringe Program- Challenge in reaching the best solution regarding reduction of the age restriction. |
| Safreed-Harmon et al., 2018 [50] | Policy responses to hepatitis C in the Nordic countries: Gaps and discrepant reporting in the Hep-Nordic study | Needle and syringe programs- There is a minimum age to access needle and syringe programs, clean needles/syringes cannot be purchased legally at pharmacies by people who inject drugs, the system does not allow anonymous participation in needle and syringe programs. |
| Leung et al., 2019 [51] | Development of Contextually-relevant Sexuality Education: Lessons from a Comprehensive Review of Adolescent Sexuality Education Across Cultures | Sex Education- No standardized Sex and Reproduction Education curriculum for schools which implies that schools have the autonomy to develop their own programs. Youths are deprived of certain knowledge about sex and sexuality |
| Rickman et al., 2018 [52] | Missed opportunities for tuberculosis prevention among patients accessing a UK HIV service | Guidelines for screening and treatment of latent tuberculosis infection in HIV-infected persons- Low rate of screening. Guidelines are complex, requiring integration of information about CD4, ART history and region of origin |
| Pezeril, 2021 [53] | We are people with no rights. HIV Necropolitics for Undocumented Migrants in Belgium | Residence permit for migrants based on HIV status- Criticism that administration is more like a “permit to die” on Belgian territory. |
| Michel et al., 2015 [54] | Insufficient access to harm reduction measures in prisons in 5 countries (PRIDE Europe): a shared European public health concern | Harm reduction in prisons- Condoms were not available in any Italian prison. ARV were accessible in all prisons in all countries, except Italy. NEP are not authorized in any prison in these countries. |
| Koppe et al., 2019 [55] | Factors associated with the informal use of HIV pre-exposure prophylaxis in Germany: a cross-sectional study | Availability of PrEP- The initially high out‐of‐pocket costs for obtaining prescription PrEP brought many users to start obtaining PrEP through informal sources, who might lack financial resources or even the knowledge where to obtain appropriate medical supervision, more likely to forgo testing before or during PrEP use |
| Müllerschön et al., 2019 [56] | Impact of health insurance status among migrants from sub-Saharan Africa on access to health care and HIV testing in Germany: a participatory cross-sectional survey | HIV treatment for migrants- Stigma, discrimination, residence issues and traumatic experiences associated with the migration process present barriers in accessing healthcare services and preventive measures. Absence of regular health insurance increased the odds of no contact with the healthcare system. |
| Faust, 2020 [57] | The prisoner citizen: juridification and the AIDS activist struggle for harm reduction in German prisons | Drug criminalization- In practice, prison conditions, administrative procedures, political constraints, and social stigmatization severely limit the prisoners’ options for access to healthcare and disease prevention. |
| Stöver et al., 2020 [58] | Agonist opioid treatment for opioid dependents in Germany 2019 | Opioid treatment- A considerable number of opioid dependents have experienced prison sentences. Implementation is the responsibility of each federal states and varies substantially from prison to prison. |
| Moazen et al., 2020 [59] | Availability, Accessibility, and Coverage of Needle and Syringe Programs in Prisons in the European Union | Harm reduction in prisons- Lack of anonymity hinders participation in needle and syringe programs |
| Santilli, 2021 [60] | Ethnography of the socio-sanitary reception in Rome. How are HIV/AIDS and hepatitis b involved in creating the construction of legal categories assigned to migrants? | Free access to health for migrants- Increasing gap between the official policies and the real provision of non-urgent medical care. The inconsistencies of Italian migration policy have been counterbalanced by the action of third sector actors and organized at the local level. |
| Di Biagio et al., 2017 [61] | PrEP in Italy: The time may be ripe but who's paying the bill? A nationwide survey on physicians' attitudes towards using antiretrovirals to prevent HIV infection | PrEP Availability- PrEP acceptance is limited by the perception that it may work only for a restricted group of individuals at very high risk. Personal knowledge of physicians may be crucial for understanding their willingness to prescribe it in the next future. |
| Voli et al., 2018 [62] | (Trans)gender citizenship in Italy: a contradiction in terms? From the parliamentary debate about Law 164/1982 to the present | Sex change- Pathologization of sex change. Gender transition does not rely on subjects’ self-definition, but rather continues to be constrained within precise clinical and legal procedures |
| Winkler, 2019 [63] | Same-Sex Marriage and Italian Exceptionalism | Registered partnerships between persons of the same sex- Conservatism of the political establishment, the long influence of the Catholic Church and the unpredictable changes in governments and parliament majorities played an important role in the endurance of heteronormativity of marriage. |
| Bil et al., 2018 [64] | The Acceptability of Pre-Exposure Prophylaxis: Beliefs of Health-Care Professionals Working in Sexually Transmitted Infections Clinics and HIV Treatment Centers | PrEP availability- Perceived potential decrease in condom use and the increase in STI prevalence, the high costs of PrEP, and the belief that it is unethical to prescribe ART to healthy individuals. |
| Struzik et al., 2021 [65] | Citizenship and pleasure: a study on harm reduction assemblages in Poland | Drug policy- Harm reduction is hampered not only by the criminalization of drug possession, but also by social constructions of drug use itself |
| Grabowska-Moroz et al., 2021 [66] | Reframing LGBT rights advocacy in the context of the rule of law backsliding. The case of Poland | Discrimination policy on sex orientation- Low level of legal protection to LGBT rights, with no specific protection from homophobic hate crime and hate speech, no legal recognition of same-sex relationships in form of civil union or marriage, no right of same-sex couples to be recognized jointly as parents of a child. Homophobia is widespread in Poland. |
| Augusto et al., 2020 [67] | Preventing HIV under financial constraints: The 2011-12 reform of the Portuguese HIV/AIDS Programme | HIV Program- Cuts in public expenditure that affected the health sector and public health programs. Delay in integrating the HIV/AIDS Program’s budget in the Directorate-General of Health, which resulted in successive delays in paying suppliers and acquiring preventive materials. |
| Guerras et al., 2022 [68] | Knowledge and Prior Use of HIV Self-Testing in Madrid and Barcelona among Men Who Have Sex with Men More than One Year after Its Legal Authorization in Spain | HIV self-testing- After the authorization of HIV self-testing, knowledge and use remain low. No specific campaign was conducted of its authorization by the Ministry of Health. |
| Kalk et al., 2018 [69] | Treatment and Intervention for Opiate Dependence in the United Kingdom: Lessons from Triumph and Failure | Harm reduction services- Actual implementation of the policy has varied, with recovery often been equated to abstinence. |
| Bikava and Kreituse, 2019 [70] | Cooperation and Collaboration Impact on Policy Development: HIV/AIDS Policy in Latvia | Plan for HIV/AIDS- Results and implemented political initiatives in HIV related topics were driven mainly by activities carried out by Non-Governmental Organizations. Delay in offering ART despite CD4 levels due to scarce funding resources. Lack of specialists, with only one hospital where HIV/AIDS patients can receive consultations and prescriptions for medicine. |
| Meireles et al., 2020 [71] | Eligibility for pre-exposure prophylaxis according to different guidelines in a cohort of HIV-negative men who have sex with men in Lisbon, Portugal | PrEP Availability- Guideline definitions are not concordant even though all guidelines are intended to identify people at higher risk of HIV. Number of PrEP users was far less than estimated to be the number of potential beneficiaries. |
| Saigal et al. 2022 [72] | BHIVA position statement on HIV, the law and the work of the clinical team | HIV Transmission Criminalization- The burden of reducing HIV transmission on the person living with HIV, inconsistent with public health initiatives which promote and enable individual responsibility within the HIV-negative population. Application of the criminal law to cases other than intentional transmission may be counterproductive to public health aims. |
| Phillips and Schembri, 2016 [73] | Narratives of HIV: measuring understanding of HIV and the law in HIV-positive patients | HIV Transmission Criminalization- PLHIV faces feelings of stigmatization and marginalization. Morals and stigma remain important to people’s thinking, with criminalization of HIV infection addressing the complexities behind sexual behavior. |
| Dodds et al., 2015 [74] | Keeping confidence: HIV and the criminal law from HIV service providers' perspectives | HIV Transmission Criminalization- Physicians feel empowerment or relief about being able to use the law as an externalized tool to help dispel their moral unease. |
| Nagington and Sandset, 2020 [75] | Putting the NHS England on trial: uncertainty-as-power, evidence and the controversy of PrEP in England | PrEP availability- NHS raised a legal uncertainty about commissioning PrEP, which can be read as a form of healthcare rationing. |
| Hillis et al. 2021 [76] | "Belt and braces approach; added benefit and … extra reassurance": a multi-stakeholder examination of the challenges to effective provision of pre-exposure prophylaxis (PrEP) for HIV prevention among men who have sex with men (MSM) in Northern and Central England | PrEP availability- Some sites appeared under strain with increased staff workload and inadequate resources. Lack of privacy in sexual health clinic waiting rooms and stigma from service providers when discussing sexuality, sexual health, or PrEP |
| Young et al., 2021 [77] | Configuring the PrEP user: framing pre-exposure prophylaxis in UK newsprint 2012-2016 | PrEP availability- PrEP users and their sexual practices were presented as a source of unknown or increased risk. PrEP users are painted as irresponsible citizens included ‘unsafe sex’ when describing condomless sex, calling PrEP a ‘party drug’, and describing ‘increased risk taking’, ‘promiscuity’ and/or a ‘reduction of condoms’. |
| White et al., 2017 [78] | Latent tuberculosis infection screening and treatment in HIV: insights from evaluation of UK practice | Latent TB infection (LTBI) screening and treatment in HIV-positive individuals- Lack of confidence in the published guidance and a view that the current guidelines are too complex. |
| Bil et al., 2015 [79] | What do Dutch MSM think of preexposure prophylaxis to prevent HIV-infection? A cross-sectional study | PrEP availability- Psychosocial determinants (e.g. feelings of shame) and PrEP characteristics as barriers for the intention to use PrEP. |
| Molina and Ortega, 2015 [80] | ¿Cómo está afectando la aplicación del nuevo marco legal sanitario a la asistencia de los inmigrantes infectados por el VIH en situación irregular en España? | Universal healthcare service- Lack of information for health-care professionals and NGOs involved in the care of immigrants hinders access of this population to health care. Only a minority knew if their Autonomous Community had specific regulations on this matter. |

# References

1.	European Centre for Disease Prevention and Control *HIV/AIDS Surveillance in Europe 2022 (2021 Data)*; 2022;

2.	The World Bank GDP per Capita (Current US$) Available online: https://data.worldbank.org/indicator/NY.GDP.PCAP.CD (accessed on 11 October 2022).

3.	Equaldex Progress of LGBTQ+ Rights across the World Available online: https://www.equaldex.com/equality-index (accessed on 28 October 2022).

4.	Di Nicola, A. *The Differing EU Member States’ Regulations on Prostitution and Their Cross-Border Implications on Women’s Rights*; Brussels, 2021;

5.	Ferreira, P.L.; Tavares, A.I.; Quintal, C.; Santana, P. EU Health Systems Classification: A New Proposal from EURO-HEALTHY. *BMC Health Serv Res* **2018**, *18*, 1–11, doi:10.1186/s12913-018-3323-3.

6.	Gaeta, M.; Campanella, F.; Capasso, L.; Schifino, G.M.; Gentile, L.; Banfi, G.; Pelissero, G.; Ricci, C. An Overview of Different Health Indicators Used in the European Health Systems. *J Prev Med Hyg* **2017**, *58*, E114–E120.

7.	World Health Organization Coverage of Essential Health Services (SDG 3.8.1) Available online: https://www.who.int/data/gho/data/themes/topics/service-coverage (accessed on 11 October 2022).

8.	Marsh, K.; Eaton, J.W.; Mahy, M.; Sabin, K.; Autenrieth, C.S.; Wanyeki, I.; Daher, J.; Ghys, P.D. Global, Regional and Country-Level 90-90-90 Estimates for 2018: Assessing Progress towards the 2020 Target. *AIDS* **2019**, *33*, S213–S226, doi:10.1097/QAD.0000000000002355.

9.	UNAIDS *UNAIDS Data 2021*; Geneva, 2021;

10.	Institute for Health Metrics and Evaluation Global Burden of Disease Available online: https://vizhub.healthdata.org/gbd-results/ (accessed on 25 April 2025).

11.	Kavanagh, M.M.; Graeden, E.; Pillinger, M.; Singh, R.; Eaneff, S.; Bendaud, V.; Gustav, R.; Erkkola, T. Understanding and Comparing HIV-Related Law and Policy Environments: Cross-National Data and Accountability for the Global AIDS Response. *BMJ Glob Health* **2020**, *5*, e003695, doi:10.1136/bmjgh-2020-003695.

12.	Schweikardt, C.; Coppieters, Y. The Development of a National HIV Plan in Belgium: Achieving Consensus despite Institutional Complexity. *Health Policy (New York)* **2015**, *119*, 1390–1398, doi:10.1016/j.healthpol.2015.05.006.

13.	Mravčík, V.; Pitoňák, M.; Hejzák, R.; Janíková, B.; Procházka, I. HIV Epidemic among Men Who Have Sex with Men in the Czech Republic, 2016: High Time for Targeted Action. *Eurosurveillance* **2017**, *22*, doi:10.2807/1560-7917.ES.2017.22.48.17-00079.

14.	Rüütel, K.; Lemsalu, L.; Lätt, S. Monitoring HIV‐indicator Condition Guided HIV Testing in Estonia. *HIV Med* **2018**, *19*, 47–51, doi:10.1111/hiv.12586.

15.	Kontautaite, A.; Matyushina-Ocheret, D.; Plotko, M.; Golichenko, M.; Kalvet, M.; Antonova, L. Study of Human Rights Violations Faced by Women Who Use Drugs in Estonia. *Harm Reduct J* **2018**, *15*, 54, doi:10.1186/s12954-018-0259-1.

16.	Ousseine, Y.M.; Lydié, N.; Velter, A. Pre-Exposure Prophylaxis in France: How Many MSM Are Eligible and How Much Will It Cost? *PLoS One* **2022**, *17*, e0278016, doi:10.1371/journal.pone.0278016.

17.	Jacomet, C.; Langlois, J.; Secher, S.; Coban, D.; Lambert, C.; Zucman, D.; Trout, H.; Maarek, R.; Billaud, E.; Certain, A. Pharmacist’s Role in HIV Care in France. Implication for Clinical Improvement of People Living with HIV Worldwide. *Pharmacol Res Perspect* **2020**, *8*, doi:10.1002/prp2.629.

18.	Annequin, M.; Villes, V.; Delabre, R.M.; Alain, T.; Morel, S.; Michels, D.; Schmidt, A.J.; Velter, A.; Rojas Castro, D. Are PrEP Services in France Reaching All Those Exposed to HIV Who Want to Take PrEP? MSM Respondents Who Are Eligible but Not Using PrEP (EMIS 2017). *AIDS Care* **2020**, *32*, 47–56, doi:10.1080/09540121.2020.1739219.

19.	Houborg, E.; Jauffret-Roustide, M. Drug Consumption Rooms: Welfare State and Diversity in Social Acceptance in Denmark and in France. *Am J Public Health* **2022**, *112*, S159–S165, doi:10.2105/AJPH.2022.306808.

20.	Dichtl, A.; Stöver, H.; Schäffer, D. HIV/HCV Unter Drogengebrauchenden in Deutschland: Die Eliminierungsziele Im Spiegel Der Praxis. *Das Gesundheitswesen* **2023**, *85*, 712–717, doi:10.1055/a-1819-6116.

21.	Seals, M. Worker Rights and Health Protection for Prostitutes: A Comparison of The Netherlands, Germany, and Nevada. *Health Care Women Int* **2015**, *36*, 784–796, doi:10.1080/07399332.2013.850085.

22.	Scholten, M.; Suárez, I.; Platten, M.; Kümmerle, T.; Jung, N.; Wyen, C.; Ernst, A.; Horn, C.; Burst, V.; Suárez, V.; et al. To Prescribe, or Not to Prescribe: Decision Making in HIV‐1 Post‐exposure Prophylaxis. *HIV Med* **2018**, *19*, 645–653, doi:10.1111/hiv.12645.

23.	Werner, R.N.; Gaskins, M.; Ahrens, J.; Jessen, H.; Kutscha, F.; Mosdzen, R.; Osswald, W.; Sander, D.; Schellberg, S.; Schwabe, K.; et al. Knowledge and Use of HIV Pre-Exposure Prophylaxis among Men Who Have Sex with Men in Berlin – A Multicentre, Cross-Sectional Survey. *PLoS One* **2018**, *13*, e0204067, doi:10.1371/journal.pone.0204067.

24.	Galli, M.; Borderi, M.; Viale, P. HIV Policy in Italy and Recommendations across the HIV Care Continuum. *Infez Med* **2020**, *28*, 17–28.

25.	Andreoni, M.; Antinori, A.; Castagna, A.; D’Arminio Monforte, A.; Di Perri, G.; Galli, M.; Gori, A.; Mussini, C.; Perno, C.F. Beyond Italian Guidelines in the Management of HIV-Positive Patient. *Infez Med* **2020**, *28*, 587–596.

26.	Sander, G.; Scandurra, A.; Kamenska, A.; MacNamara, C.; Kalpaki, C.; Bessa, C.F.; Laso, G.N.; Parisi, G.; Varley, L.; Wolny, M.; et al. Overview of Harm Reduction in Prisons in Seven European Countries. *Harm Reduct J* **2016**, *13*, 28, doi:10.1186/s12954-016-0118-x.

27.	Tibaldi, C.; Masuelli, G.; Sansone, M.; Tassis, B.; Cetin, I.; Franceschetti, L.; Spinillo, A.; Simonazzi, G.; Vimercati, A.; Dalzero, S.; et al. Vaginal Delivery in Women with HIV in Italy: Results of 5 Years of Implementation of the National SIGO-HIV Protocol. *Infection* **2019**, *47*, 981–990, doi:10.1007/s15010-019-01336-z.

28.	Ferrara, P.; Gianfredi, V. The Italian PrEPventHIV Challenge: A Scoping Systematic Review on HIV Pre-Exposure Prophylaxis Monitoring in Italy. *Acta Biomed* **2022**, *93*, e2022164, doi:10.23750/abm.v93i3.12766.

29.	Goletti, D.; Navarra, A.; Petruccioli, E.; Cimaglia, C.; Compagno, M.; Cuzzi, G.; De Carli, G.; Fondaco, L.; Franzetti, F.; Giannetti, A.; et al. Latent Tuberculosis Infection Screening in Persons Newly-Diagnosed with HIV Infection in Italy: A Multicentre Study Promoted by the Italian Society of Infectious and Tropical Diseases. *International Journal of Infectious Diseases* **2020**, *92*, 62–68, doi:10.1016/j.ijid.2019.12.031.

30.	Toxopeus, D.C.M.; Pell, C.L.; Westrhenen, N.B.; Smit, C.; Wit, F.W.N.M.; Ondoa, P.; Reiss, P.; Boender, T.S. Compliance with Laboratory Monitoring Guidelines in Outpatient HIV Care: A Qualitative Study in the Netherlands. *AIDS Care* **2019**, *31*, 840–847, doi:10.1080/09540121.2018.1563280.

31.	Evenblij, K.; Verbon, A.; van Leth, F. Intention of Physicians to Implement Guidelines for Screening and Treatment of Latent Tuberculosis Infection in HIV-Infected Patients in The Netherlands: A Mixed-Method Design. *BMC Public Health* **2016**, *16*, 915, doi:10.1186/s12889-016-3539-2.

32.	Tokar, A.; Osborne, J.; Hengeveld, R.; Lazarus, J. V.; Broerse, J.E.W. ‘I Don’t Want Anyone to Know’: Experiences of Obtaining Access to HIV Testing by Eastern European, Non-European Union Sex Workers in Amsterdam, the Netherlands. *PLoS One* **2020**, *15*, e0234551, doi:10.1371/journal.pone.0234551.

33.	Malinowska-Sempruch, K. Shaping Drug Policy in Poland. *International Journal of Drug Policy* **2016**, *31*, 32–38, doi:10.1016/j.drugpo.2016.02.018.

34.	Balayan, T.; Begovac, J.; Skrzat‐Klapaczyńska, A.; Aho, I.; Alexiev, I.; Bukovinova, P.; Salemovic, D.; Gokengin, D.; Harxhi, A.; Holban, T.; et al. Where Are We with Pre‐exposure Prophylaxis Use in Central and Eastern Europe? Data from the Euroguidelines in Central and Eastern Europe (ECEE) Network Group. *HIV Med* **2021**, *22*, 67–72, doi:10.1111/hiv.12960.

35.	Meireles, P.; Plankey, M.; Rocha, M.; Brito, J.; Mendão, L.; Barros, H. Different Guidelines for Pre-Exposure Prophylaxis (PrEP) Eligibility Estimate HIV Risk Differently: An Incidence Study in a Cohort of HIV-Negative Men Who Have Sex with Men, Portugal, 2014–2018. *Eurosurveillance* **2020**, *25*, doi:10.2807/1560-7917.ES.2020.25.28.1900636.

36.	Sacramento, O. Individuals, Structures, and Risks: An Overview of Primary HIV Prevention in Portugal. *Cad Saude Publica* **2016**, *32*, doi:10.1590/0102-311X00129715.

37.	Gómez Castellá, J.; Díaz Franco, A.; Polo Rodríguez, R.; del Amo Valero, J. Approaches to STI Prevention and Control in a Highly Decentralized State: An Integrative Review. *Frontiers in Reproductive Health* **2022**, *4*, doi:10.3389/frph.2022.725646.

38.	Gogishvili, M.; Costa, S.A.; Flórez, K.; Huang, T.T. Policy Implementation Analysis on Access to Healthcare among Undocumented Immigrants in Seven Autonomous Communities of Spain, 2012–2018. *BMJ Open* **2021**, *11*, e045626, doi:10.1136/bmjopen-2020-045626.

39.	García, L.M.; Iniesta, C.; Garrido, J.; Fuster, M.J.; Pujol, F.; Meulbroek, M.; Poveda, T.; Riera, M.; Antela, A.; Moreno, S.; et al. Profilaxis Preexposición al VIH En España: Situación Política y Administrativa. *Enferm Infecc Microbiol Clin* **2019**, *37*, 656–660, doi:10.1016/j.eimc.2018.05.012.

40.	Gogishvili, M.; Flórez, K.R.; Costa, S.A.; Huang, T.T.-K. A Qualitative Study on Mixed Experiences of Discrimination and Healthcare Access among HIV-Positive Immigrants in Spain. *BMC Public Health* **2021**, *21*, 385, doi:10.1186/s12889-021-10388-6.

41.	Iniesta, C.; Folch, C.; Meyer, S.; Vázquez, M.; Casabona, J.; Díaz, A. Would Eligible Gay, Bisexual and Other Men Who Have Sex with Men Use PrEP? Awareness, Knowledge, Eligibility and Intention to Use PrEP among EMIS-2017 Participants in Spain. *Prev Med (Baltim)* **2022**, *156*, 106962, doi:10.1016/j.ypmed.2022.106962.

42.	Suárez-García, I.; González, J.; Berenguer, J.; García, F.; Portilla, J.; Muga, R.; Moreno, S.; Jarrín, I. Reasons for Noncompliance with the National Guidelines for Initial Antiretroviral Therapy of HIV-Infected Patients in Spain, 2010–2015. *Enferm Infecc Microbiol Clin* **2019**, *37*, 580–587, doi:10.1016/j.eimc.2019.02.007.

43.	Hoyos, J.; Guerras, J.-M.; Maté, T.; Agustí, C.; Fernández-López, L.; Fuente, L. de la; Belza, M.-J. Opinions towards Key Operational Aspects for the Implementation of HIV Self-Testing in Spain: A Comparison between Stakeholders and Potential Users. *Int J Environ Res Public Health* **2021**, *18*, 1428, doi:10.3390/ijerph18041428.

44.	Koerting, A.; Polo, R.; Vázquez, M.C.; Del Amo, J. [The Social Pact for the Non-Discrimination and Equal Treatment Associated with HIV Development]. *Rev Esp Salud Publica* **2019**, *93*.

45.	Viciana-Fernández, P.; Falcó, V.; Castaño, M.; de los Santos-Gil, I.; Olalla-Sierra, J.; Hernando, A.; Deig, E.; Clotet, B.; Knobel, H.; Podzamczer, D.; et al. Barreras Para El Inicio Del Tratamiento Antirretroviral En Pacientes Con Virus de La Inmunodeficiencia Humana e Indicación de Tratamiento En España. ¿Por Qué No Inician Tratamiento Quienes Lo Tienen Indicado? Estudio Bridgap. *Enferm Infecc Microbiol Clin* **2015**, *33*, 397–403, doi:10.1016/j.eimc.2014.06.012.

46.	Herder, T.; Agardh, A. Navigating between Rules and Reality: A Qualitative Study of HIV Positive MSM’s Experiences of Communication at HIV Clinics in Sweden about the Rules of Conduct and Infectiousness. *AIDS Care* **2019**, *31*, 1304–1310, doi:10.1080/09540121.2019.1566590.

47.	Amroussia, N.; Holmström, C.; Ouis, P. Migrants in Swedish Sexual and Reproductive Health and Rights Related Policies: A Critical Discourse Analysis. *Int J Equity Health* **2022**, *21*, 125, doi:10.1186/s12939-022-01727-z.

48.	Karlsson, N.; Berglund, T.; Ekström, A.M.; Hammarberg, A.; Tammi, T. Could 30 Years of Political Controversy on Needle Exchange Programmes in Sweden Contribute to Scaling-up Harm Reduction Services in the World? *Nordic Studies on Alcohol and Drugs* **2021**, *38*, 66–88, doi:10.1177/1455072520965013.

49.	Barrett, D.; Petersson, F.; Turner, R. Best Interests and Low Thresholds: Legal and Ethical Issues Relating to Needle and Syringe Services for under 18s in Sweden. *Harm Reduct J* **2022**, *19*, 15, doi:10.1186/s12954-022-00597-6.

50.	Safreed-Harmon, K.; Hetherington, K.L.; Aleman, S.; Alho, H.; Dalgard, O.; Frisch, T.; Gottfredsson, M.; Weis, N.; Lazarus, J. V. Policy Responses to Hepatitis C in the Nordic Countries: Gaps and Discrepant Reporting in the Hep-Nordic Study. *PLoS One* **2018**, *13*, e0190146, doi:10.1371/journal.pone.0190146.

51.	Leung, H.; Shek, D.; Leung, E.; Shek, E. Development of Contextually-Relevant Sexuality Education: Lessons from a Comprehensive Review of Adolescent Sexuality Education Across Cultures. *Int J Environ Res Public Health* **2019**, *16*, 621, doi:10.3390/ijerph16040621.

52.	Rickman, H.M.; Miller, R.F.; Morris-Jones, S.; Kellgren, L.; Edwards, S.G.; Grant, A.D. Missed Opportunities for Tuberculosis Prevention among Patients Accessing a UK HIV Service. *Int J STD AIDS* **2018**, *29*, 1234–1237, doi:10.1177/0956462418773010.

53.	Pezeril, C. We Are People with No Rights. HIV Necropolitics for Undocumented Migrants in Belgium. *L’Année du Maghreb* **2021**, 113–130, doi:10.4000/anneemaghreb.7979.

54.	Michel, L.; Lions, C.; Van Malderen, S.; Schiltz, J.; Vanderplasschen, W.; Holm, K.; Kolind, T.; Nava, F.; Weltzien, N.; Moser, A.; et al. Insufficient Access to Harm Reduction Measures in Prisons in 5 Countries (PRIDE Europe): A Shared European Public Health Concern. *BMC Public Health* **2015**, *15*, 1093, doi:10.1186/s12889-015-2421-y.

55.	Koppe, U.; Marcus, U.; Albrecht, S.; Jansen, K.; Jessen, H.; Gunsenheimer‐Bartmeyer, B.; Bremer, V. Factors Associated with the Informal Use of HIV Pre‐exposure Prophylaxis in Germany: A Cross‐sectional Study. *J Int AIDS Soc* **2019**, *22*, doi:10.1002/jia2.25395.

56.	Müllerschön, J.; Koschollek, C.; Santos-Hövener, C.; Kuehne, A.; Müller-Nordhorn, J.; Bremer, V. Impact of Health Insurance Status among Migrants from Sub-Saharan Africa on Access to Health Care and HIV Testing in Germany: A Participatory Cross-Sectional Survey. *BMC Int Health Hum Rights* **2019**, *19*, 10, doi:10.1186/s12914-019-0189-3.

57.	Faust, F. The Prisoner Citizen: Juridification and the AIDS Activist Struggle for Harm Reduction in German Prisons. *Crit Public Health* **2021**, *31*, 17–29, doi:10.1080/09581596.2020.1841113.

58.	Stöver, H.; Michels, I.I.; Gerlach, R. Agonist Opioid Treatment for Opioid Dependents in Germany 2019. *Heroin Addiction Related Clinical Problems* **2020**.

59.	Moazen, B.; Dolan, K.; Saeedi Moghaddam, S.; Lotfizadeh, M.; Duke, K.; Neuhann, F.; Stöver, H.; Jahn, A. Availability, Accessibility, and Coverage of Needle and Syringe Programs in Prisons in the European Union. *Epidemiol Rev* **2020**, *42*, 19–26, doi:10.1093/epirev/mxaa003.

60.	Santilli, C. Ethnography of the Socio-Sanitary Reception in Rome. How Are HIV/AIDS and Hepatitis b Involved in Creating the Construction of Legal Categories Assigned to Migrants? *Int J Migr Health Soc Care* **2021**, *17*, 142–154, doi:10.1108/IJMHSC-07-2019-0061.

61.	Di Biagio, A.; Riccardi, N.; Signori, A.; Maserati, R.; Nozza, S.; Gori, A.; Bonora, S.; Borderi, M.; Ripamonti, D.; Rossi, M.C.; et al. PrEP in Italy: The Time May Be Ripe but Who’s Paying the Bill? A Nationwide Survey on Physicians’ Attitudes towards Using Antiretrovirals to Prevent HIV Infection. *PLoS One* **2017**, *12*, e0181433, doi:10.1371/journal.pone.0181433.

62.	Voli, S. (Trans)Gender Citizenship in Italy: A Contradiction in Terms? From the Parliamentary Debate about Law 164/1982 to the Present. *Modern Italy* **2018**, *23*, 201–214, doi:10.1017/mit.2018.8.

63.	Winkler, M.M. Same-Sex Marriage and Italian Exceptionalism. *ICL Journal* **2019**, *12*, 431–456, doi:10.1515/icl-2018-0037.

64.	Bil, J.P.; Hoornenborg, E.; Prins, M.; Hogewoning, A.; Dias Goncalves Lima, F.; de Vries, H.J.C.; Davidovich, U. The Acceptability of Pre-Exposure Prophylaxis: Beliefs of Health-Care Professionals Working in Sexually Transmitted Infections Clinics and HIV Treatment Centers. *Front Public Health* **2018**, *6*, doi:10.3389/fpubh.2018.00005.

65.	Struzik, J. Citizenship and Pleasure: A Study on Harm Reduction Assemblages in Poland. *Crit Public Health* **2021**, *31*, 30–42, doi:10.1080/09581596.2020.1849562.

66.	Grabowska-Moroz, B.; Wójcik, A. Reframing LGBT Rights Advocacy in the Context of the Rule of Law Backsliding. *Intersections* **2021**, *7*, 85–103, doi:10.17356/ieejsp.v7i4.852.

67.	Augusto, G.F.; Aldir, I.; Bettencourt, J.; Melo, T.; Dias, S.F.; Abrantes, A.; Martins, M.O. Preventing HIV under Financial Constraints: The 2011–12 Reform of the Portuguese HIV/AIDS Programme. *Health Policy (New York)* **2020**, *124*, 339–344, doi:10.1016/j.healthpol.2020.02.010.

68.	Guerras, J.-M.; Belza, M.-J.; Fuster, M.-J.; Fuente, L. de la; García de Olalla, P.; Palma, D.; García-Pérez, J.-N.; Hoyos, J. Knowledge and Prior Use of HIV Self-Testing in Madrid and Barcelona among Men Who Have Sex with Men More than One Year after Its Legal Authorization in Spain. *Int J Environ Res Public Health* **2022**, *19*, 1096, doi:10.3390/ijerph19031096.

69.	Kalk, N.J.; Robertson, J.R.; Kidd, B.; Day, E.; Kelleher, M.J.; Gilvarry, E.; Strang, J. Treatment and Intervention for Opiate Dependence in the United Kingdom: Lessons from Triumph and Failure. *Eur J Crim Pol Res* **2018**, *24*, 183–200, doi:10.1007/s10610-017-9364-z.

70.	Bikava, I.; Kreituse, I. Cooperation and Collaboration Impact on Policy Development: HIV/AIDS Policy in Latvia. *Sociology Study* **2019**, *9*, doi:10.17265/2159-5526/2019.03.001.

71.	Meireles, P.; Plankey, M.; Rocha, M.; Rojas, J.; Brito, J.; Barros, H. Eligibility for Pre-Exposure Prophylaxis According to Different Guidelines in a Cohort of HIV-Negative Men Who Have Sex with Men in Lisbon, Portugal. *Sexuality Research and Social Policy* **2020**, *17*, 688–699, doi:10.1007/s13178-019-00426-9.

72.	Saigal, P.; Phillips, M.; Bernard, E.; Douglas, C.; Poulton, M.; Smithson, K.; Sparrowhawk, A.; Ward, C.; Weait, M. BHIVA Position Statement on HIV, the Law and the Work of the Clinical Team. *Int J STD AIDS* **2022**, *33*, 1223–1228, doi:10.1177/09564624221132407.

73.	Phillips, M.D.; Schembri, G. Narratives of HIV: Measuring Understanding of HIV and the Law in HIV-Positive Patients. *Journal of Family Planning and Reproductive Health Care* **2016**, *42*, 30–35, doi:10.1136/jfprhc-2013-100789.

74.	Dodds, C.; Weait, M.; Bourne, A.; Egede, S. Keeping Confidence: HIV and the Criminal Law from HIV Service Providers’ Perspectives. *Crit Public Health* **2015**, *25*, 410–426, doi:10.1080/09581596.2015.1019835.

75.	Nagington, M.; Sandset, T. Putting the NHS England on Trial: Uncertainty-as-Power, Evidence and the Controversy of PrEP in England. *Med Humanit* **2020**, *46*, 176–179, doi:10.1136/medhum-2019-011780.

76.	Hillis, A.; Germain, J.; Hibbert, M.P.; Hope, V.; Van Hout, M.C. “Belt and Braces Approach; Added Benefit and … Extra Reassurance”: A Multi-Stakeholder Examination of the Challenges to Effective Provision of Pre-Exposure Prophylaxis (PrEP) for HIV Prevention among Men Who Have Sex with Men (MSM) in Northern and Central England. *AIDS Care* **2021**, *33*, 736–745, doi:10.1080/09540121.2021.1871721.

77.	Young, I.; Boydell, N.; Patterson, C.; Hilton, S.; McDaid, L. Configuring the PrEP User: Framing Pre-Exposure Prophylaxis in UK Newsprint 2012–2016. *Cult Health Sex* **2021**, *23*, 772–787, doi:10.1080/13691058.2020.1729420.

78.	White, H.A.; Miller, R.F.; Pozniak, A.L.; Lipman, M.C.I.; Stephenson, I.; Wiselka, M.J.; Pareek, M. Latent Tuberculosis Infection Screening and Treatment in HIV: Insights from Evaluation of UK Practice. *Thorax* **2017**, *72*, 180–182, doi:10.1136/thoraxjnl-2016-209063.

79.	Bil, J.P.; Davidovich, U.; van der Veldt, W.M.; Prins, M.; de Vries, H.J.C.; Sonder, G.J.B.; Stolte, I.G. What Do Dutch MSM Think of Preexposure Prophylaxis to Prevent HIV-Infection? A Cross-Sectional Study. *AIDS* **2015**, *29*, 955–964, doi:10.1097/QAD.0000000000000639.

80.	Pérez-Molina, J.A.; Pulido, F. How Is the Implementation of the New Legal Framework for Health Care Affecting HIV-Infected Immigrants in an Irregular Situation in Spain? *Enferm Infecc Microbiol Clin* **2015**, *33*, 437–445, doi:10.1016/j.eimc.2014.10.017.
